# Supplementary material for: Myelination and excitation-inhibition balance synergistically shape structure-function coupling across the human cortex
Source: Nat Commun. 2023 Sep 30;14:6115. doi: 10.1038/s41467-023-41686-9 (PMC10542365; doi:10.1038/s41467-023-41686-9)
Supplement: Supplementary file 1 — Supplementary Information [file 41467_2023_41686_MOESM1_ESM.pdf]

Myelination and excitation-inhibition balance synergistically shape structure-function coupling across the human cortex

Panagiotis Fotiadis,[1,2,†] Matthew Cieslak,[3] Xiaosong He,[4] Lorenzo Caciagli,[2] Mathieu Ouellet,[2] Theodore D. Satterthwaite,[3] Russell T. Shinohara,[5,6] Dani S. Bassett [2,3,7-10,†]

[1] Department of Neuroscience, Perelman School of Medicine, University of Pennsylvania, Philadelphia, PA 19104, USA
[2] Department of Bioengineering, University of Pennsylvania, Philadelphia, PA 19104, USA
[3] Department of Psychiatry, Perelman School of Medicine, University of Pennsylvania, Philadelphia, PA 19104, USA
[4] Department of Psychology, University of Science and Technology of China, Hefei, Anhui, 230026, China
[5] Penn Statistics in Imaging and Visualization Center, Department of Biostatistics, Epidemiology, and Informatics, University of Pennsylvania, Philadelphia, PA 19104, USA
[6] Center for Biomedical Image Computing & Analytics, University of Pennsylvania, Philadelphia, PA 19104, USA
[7] Department of Electrical & Systems Engineering, University of Pennsylvania, Philadelphia, PA 19104, USA
[8] Department of Physics & Astronomy, University of Pennsylvania, Philadelphia, PA 19104, USA
[9] Department of Neurology, Perelman School of Medicine, University of Pennsylvania, Philadelphia, PA 19104, USA
[10] Santa Fe Institute, Santa Fe, NM 87501, USA

[†] Corresponding authors:
Panagiotis Fotiadis (panosf@pennmedicine.upenn.edu), Dani S. Bassett (dsb@seas.upenn.edu)

SUPPLEMENTAL MATERIAL

To examine the reproducibility of our findings, we repeated the following three additional analyses.

SUPPLEMENTAL ANALYSIS 1:

In this first supplemental analysis, we repeated our atlas-based methodology reported in the main text and analyzed the Human Connectome Project (HCP) sample (n=100) using an additional commonly-used atlas: the HCP multi-modal cortical parcellation (360 brain regions).

Structure-Function Coupling Variations along the Cortical Hierarchy

In the 7 resting-state systems, structure-function coupling (SFC) was highest in the primary visual and somatomotor cortices, intermediate in the default mode, dorsal attention, fronto-parietal, and ventral attention association systems, and lowest in the limbic system (**Supplemental Figure 1A**; **Supplemental Table 5**). A decrease in SFC along the unimodal-transmodal hierarchy was also evident along the principal functional gradient, in the form of a significant negative correlation between a brain region's SFC and its assigned principal gradient scalar (**Supplemental Figure 1C**; r=-0.41; p_{spin}=5x10^{-4}); lower assignments within this gradient capture primary sensory and motor regions, whereas higher assignments capture regions within the default mode network. Across the 5 cyto-architectonic classes, SFC gradually decreased from granular (typically capturing sensory regions) to agranular (typically capturing motor and association regions) types and displayed its lowest value in the polar cortical type (**Supplemental Figure 1B**; **Supplemental Table 6**). Similarly, we observed a significant negative correlation between a brain region's SFC and its assigned location along the BigBrain gradient of microstructure profile covariance (**Supplemental Figure 1D**; r=-0.33; p_{spin}=0.032); primary sensory regions occupy the lower end of this gradient while limbic regions represent its apex.

We next computed each brain region's average temporal SFC variance across subjects and examined its heterogeneous expression along the unimodal (sensory)-transmodal (association) hierarchy. In contrast to SFC, temporal SFC variance was highest in the limbic system, intermediate in the default mode and fronto-parietal systems, and lowest in the primary visual, somatomotor, dorsal attention, and ventral attention systems (**Supplemental Figure 2A**; **Supplemental Table 7**); a pronounced increase in temporal SFC variance was observed along the unimodal-transmodal hierarchy, as captured by the principal gradient (**Supplemental Figure 2C**; r=0.42; p_{spin}=0.007). Using cyto-architectonic annotations, temporal SFC variance (unlike SFC itself) was highest in the polar cortical type; the remaining 4 cortical types displayed similar degrees of temporal SFC variance (**Supplemental Figure 2B**; **Supplemental Table 8**). Under the more continuous BigBrain gradient, we observed a significant positive correlation between a brain region's temporal SFC variance and its assigned location along the gradient (**Supplemental Figure 2D**; r=0.48; p_{spin}=0.011). As with the Schaefer atlas, in order to ensure that the correlations observed between a brain region's temporal SFC variance and its location in the sensory-association hierarchy (as shown in **Supplementary Figures 2C** and **2D**) were not confounded by

the presence of any outlier regions, we repeated the aforementioned analyses after excluding the outlier brain regions. An outlier brain region was defined as one that exhibited a temporal SFC variance at least three standard deviations away from the mean (*n*=7). Both correlations remained significant (temporal SFC variance vs. principal functional gradient: r=0.40; p_{spin}=0.007, and temporal SFC variance vs. BigBrain gradient: r=0.46; p_{spin}=0.015)—same as when including the outliers in the analysis.

Biological Correlates of Structure-Function Coupling: Whole-brain perspective

Across the 360 brain regions defined by the HCP multi-modal cortical parcellation, we observed a significant positive correlation between SFC and intracortical myelin content (**Supplemental Figure 3A**; r=0.53; $p_{spin}<10^{-4}$), and a negative correlation between temporal SFC variance and intracortical myelin content (**Supplemental Figure 3B**; r=-0.31; p_{spin}=0.034). Higher SFC values corresponded to larger Hurst exponents and thus a decreased excitation-inhibition (EI) ratio (**Supplemental Figure 3C**; r=0.37; p_{spin}=0.008), whereas higher temporal variance in SFC corresponded to lower Hurst exponents and thus a heightened EI-ratio (**Supplemental Figure 3D**; r=-0.56; $p_{spin}<10^{-4}$). There was no significant association, however, between SFC and the Hurst exponent across temporal windows (average Spearman's ρ across brain regions: -0.02; $p_{fisher\ (FDR\text{-}corrected)}$=1), indicating that SFC and EI-ratio do not co-fluctuate over short periods of time (i.e., the duration of the fMRI scan), when examined on the macroscale level.

To ensure that the association between a region's SFC and either biological marker was independent of the other marker and also independent from that region's position along the cortical hierarchy, we re-examined the above relationships using multiple linear regression models. We found that SFC (dependent variable) was independently and positively correlated with intracortical myelin content (β_{stand}=0.375; 95% non-parametric bootstrap confidence interval [*BCI*]=[0.379, 0.381]; $p<10^{-4}$; variance inflation factor [*VIF*]=1.45) and with the Hurst exponent (β_{stand}=0.368; 95% *BCI*=[0.368, 0.370]; $p<10^{-4}$; *VIF*=1.30), after adjusting for the other biological marker, the interaction effect between intracortical myelination and the Hurst exponent, as well as the principal gradient and BigBrain scalar assignments. Further, the correspondence between temporal SFC variance (dependent variable) and the Hurst exponent (β_{stand}=-0.534; 95% *BCI*=[-0.536, -0.534]; $p<10^{-4}$; *VIF*=1.30), but not intracortical myelin content (β_{stand}=-0.008; 95% *BCI* = [-0.005, -0.003]; p=0.90; *VIF*=1.45), remained significant after adjusting for the other marker, the interaction effect between intracortical myelination and the Hurst exponent, and the principal gradient and BigBrain scalar assignments. Similarly to the atlas-based analysis reported in the manuscript, the Hurst exponent was found to significantly mediate the correlation between intracortical myelination and temporal SFC variance (total effect=-0.0045; $p<10^{-4}$, indirect effect=-0.0014; *BCI*=[-0.0025, -0.0004]). In other words, the Hurst exponent (i.e., EI-ratio) accounted for 31.1% of the correlation between intracortical myelination and temporal SFC variance.

Biological Correlates of Structure-Function Coupling: Regional perspective

As with the atlas-based analysis reported in the manuscript, we begin with the cyto-architectonic class that displayed the overall highest SFC: the granular type. We observed a significant positive association between SFC (dependent variable) and the Hurst exponent but not with intracortical myelin content, after adjusting for the effects of the other biological marker (**Supplemental Table**

9A). In the parietal and frontal types, we observed a significant positive association between SFC and the Hurst exponent as well as the intracortical myelin content (**Supplemental Table 9A**). Within the agranular cyto-architectonic class, we observed that SFC was positively correlated only with intracortical myelin content but not with the Hurst exponent, within the same regression model (**Supplemental Table 9A**). Taking these results together, we once again notice a distinct pattern as we transition from granular to agranular cortical regions: a gradual shift from the Hurst exponent to intracortical myelin content as being the principal predictor of SFC (as supported by the numerical changes in the standardized β and false discovery rate-adjusted p-values: **Supplemental Table 9A**; **Figure 7**). As with the atlas-based analysis using the Schaefer 400 parcellation reported in the manuscript, the polar cortical regions were once again an exception to this rule (**Supplemental Table 9A**).

Interestingly—and in agreement with the atlas-based results reported in the manuscript—dynamic regulation of temporal SFC variance (as opposed to SFC itself) was more persistently dependent upon the Hurst exponent, across all cyto-architectonic classes, after adjusting for the effects of intracortical myelin content (**Supplemental Table 9B**).

SUPPLEMENTAL ANALYSIS 2:

Next, we repeated the same atlas-based methodology to analyze the subjects within the HCP sample (n=100) reported in the main text, with one difference: instead of averaging the demeaned and normalized pre-processed time series corresponding to the four runs into one average run (1200 volumes), we concatenated all four runs across time (1200 volumes x 4 runs) for each subject. The results of this analyses were qualitatively identical to the ones reported in the main text for both cortical parcellations (Schaefer cortical parcellation: 400 brain regions; HCP multi-modal cortical parcellation: 360 brain regions); we separately report the results corresponding to each cortical parcellation below.

Supplemental Analysis 2A: Schaefer cortical parcellation (400 brain regions)

Structure-Function Coupling Variations along the Cortical Hierarchy

In the 7 resting-state systems, SFC was highest in the primary visual and somatomotor cortices, intermediate in the default mode, dorsal attention, fronto-parietal, and ventral attention association systems, and lowest in the limbic system (**Supplemental Figure 4A**; **Supplemental Table 10**). A decrease in SFC along the unimodal-transmodal hierarchy was also evident along the principal functional gradient, in the form of a significant negative correlation between a brain region's SFC and its assigned principal gradient scalar (**Supplemental Figure 4C**; r=-0.34; p_{spin}=0.009); lower assignments within this gradient capture primary sensory and motor regions, whereas higher assignments capture regions within the default mode network. Across the 5 cyto-architectonic classes, SFC gradually decreased from granular (typically capturing sensory regions) to agranular (typically capturing motor and association regions) types and displayed its lowest value in the polar cortical type (**Supplemental Figure 4B**; **Supplemental Table 11**). Similarly, we observed a significant negative correlation between a brain region's SFC and its assigned location along the BigBrain gradient of microstructure profile covariance (**Supplemental Figure 4D**; r=-0.39;

p_{spin}=0.025); primary sensory regions occupy the lower end of this gradient while limbic regions represent its apex.

Next, in order to examine how much SFC deviated from its mean value over time, we assessed its moment-to-moment variance throughout the duration of the resting-state fMRI scan. Specifically, we computed each brain region's average temporal SFC variance across subjects and examined its heterogeneous expression along the unimodal (sensory)-transmodal (association) hierarchy. In contrast to SFC, temporal SFC variance was highest in the limbic system (**Supplemental Figure 5A**; **Supplemental Table 12**); an increase in temporal SFC variance was observed along the unimodal-transmodal hierarchy, as captured by the principal gradient (**Supplemental Figure 5C**; r=0.20; p_{spin}=0.096). Using cyto-architectonic annotations, temporal SFC variance (unlike SFC itself) was highest in the polar cortical type; the remaining 4 cortical types displayed—for the most part—similar degrees of temporal SFC variance (**Supplemental Figure 5B**; **Supplemental Table 13**). Under the more continuous BigBrain gradient, we observed a significant positive correlation between a brain region's temporal SFC variance and its assigned location along the gradient (**Supplemental Figure 5D**; r=0.32; p_{spin}=0.035). To ensure that the correlations observed between a brain region's temporal SFC variance and its location in the sensory-association hierarchy (as shown in **Supplemental Figures 5C** and **5D**) were not confounded by the presence of any outlier regions, we repeated the aforementioned analyses after excluding the outlier brain regions. As before, an outlier brain region was defined as one that exhibited a temporal SFC variance at least three standard deviations away from the mean (n=8). Both correlations remained qualitatively the same: the association between temporal SFC variance and principal functional gradient remained positive, albeit non-significant (r=0.17; p_{spin}=0.108), whereas the association between temporal SFC variance and the BigBrain gradient remained significant (r=0.29; p_{spin}=0.040)—same as when the outliers were included.

Biological Correlates of Structure-Function Coupling: Whole-brain perspective

Across the 400 brain regions defined by the Schaefer parcellation, we observed a significant positive correlation between SFC and intracortical myelin content (**Supplemental Figure 6A**; r=0.49; p_{spin}=10^{-4}), and a negative, albeit non-significant, correlation between temporal SFC variance and intracortical myelin content (**Supplemental Figure 6B**; r=-0.08; p_{spin}=0.311). Higher SFC values corresponded to larger Hurst exponents and thus a decreased EI-ratio (**Supplemental Figure 6C**; r=0.41; p_{spin}=9x10^{-4}), whereas higher temporal variance in SFC corresponded to lower Hurst exponents and thus a heightened EI-ratio (**Supplemental Figure 6D**; r=-0.44; p_{spin}<10^{-4}).

To ensure that the association between a region's SFC and either biological marker was independent of the other marker and also independent from that region's position along the cortical hierarchy, we re-examined the above relationships using multiple linear regression models. We found that SFC (dependent variable) was independently and positively correlated with intracortical myelin content (β_{stand}=0.382; 95% non-parametric bootstrap confidence interval [BCI]=[0.381, 0.382]; p<10^{-4}; variance inflation factor [VIF]=1.85) and with the Hurst exponent (β_{stand}=0.366; 95% BCI=[0.367, 0.369]; p<10^{-4}; VIF=1.24), after adjusting for the other biological marker, the interaction effect between intracortical myelination and the Hurst exponent, as well as the principal gradient and BigBrain scalar assignments. Further, the correspondence between temporal SFC variance (dependent variable) and both intracortical myelin content (β_{stand}=0.145; 95%

BCI=[0.143, 0.144]; p=4x10^{-4}; *VIF*=1.85) and the Hurst exponent (β_{stand}=-0.406; 95% *BCI*=[-0.405, -0.403]; p<10^{-4}; *VIF*=1.24), remained significant after adjusting for the other marker, the interaction effect between intracortical myelination and the Hurst exponent, and the principal gradient and BigBrain scalar assignments. Notably, the interaction effect between intracortical myelination and the Hurst exponent was significant within this model (β_{stand}=0.345; 95% *BCI*=[0.342, 0.345]; p<10^{-4}; *VIF*=1.08); a potential causal relationship between temporal SFC variance, intracortical myelination, and the Hurst exponent was further explored via a mediation model. Notably, the Hurst exponent was found to significantly mediate the correlation between intracortical myelination and temporal SFC variance (total effect=-0.005; p<10^{-4}, indirect effect=-0.002; *BCI*=[-0.0037 -0.0007]). In other words, the Hurst exponent (i.e., EI-ratio) accounted for 40% of the correlation between intracortical myelination and temporal SFC variance.

Biological Correlates of Structure-Function Coupling: Regional perspective

We begin with the cyto-architectonic class that displayed the highest SFC: the granular type. We observed a significant positive association between SFC (dependent variable) and the Hurst exponent but not with intracortical myelin content, after adjusting for the effects of the other biological marker (**Supplemental Table 14A**). In the parietal and frontal types, we observed a significant positive association between SFC and the Hurst exponent as well as the intracortical myelin content (**Supplemental Table 14A**). Within the agranular cyto-architectonic class, we observed that SFC was positively correlated only with intracortical myelin content but not with the Hurst exponent, within the same regression model (**Supplemental Table 14A**). Taking these results together, we notice once again a distinct pattern as we transition from granular to agranular cortical regions: a gradual shift from the Hurst exponent to intracortical myelin content as being the principal predictor of SFC (as supported by the numerical changes in the standardized β and false discovery rate-adjusted p-values: **Supplemental Table 14A**; **Figure 7**). Importantly, this pattern was also reproduced with the HCP multi-modal (360 regions) cortical parcellation (**Supplemental Analysis 2B** below; **Supplemental Table 19**). Notably, the cortical type with the lowest SFC and relatively high levels of granularization—the polar type—was an exception to this rule, with SFC not being significantly correlated with either intracortical myelin content or the Hurst exponent (**Supplemental Table 14A**; **Supplemental Material: Methodological Considerations and Study Limitations**).

Interestingly, dynamic regulation of temporal SFC variance (as opposed to SFC itself) was more persistently dependent upon the Hurst exponent, across the cyto-architectonic classes. Specifically, temporal SFC variance independently and significantly correlated only with the Hurst exponent across all cortical types, after adjusting for the effects of intracortical myelin content (**Supplemental Table 14B**).

Supplemental Analysis 2B: HCP multi-modal cortical parcellation (360 brain regions)

Structure-Function Coupling Variations along the Cortical Hierarchy

In the 7 resting-state systems, SFC was highest in the primary visual and somatomotor cortices, intermediate in the default mode, dorsal attention, fronto-parietal, and ventral attention association

systems, and lowest in the limbic system (**Supplemental Figure 7A**; **Supplemental Table 15**). A decrease in SFC along the unimodal-transmodal hierarchy was also evident along the principal functional gradient, in the form of a significant negative correlation between a brain region's SFC and its assigned principal gradient scalar (**Supplemental Figure 7C**; r=-0.40; p_{spin}=4.5x10^{-4}); lower assignments within this gradient capture primary sensory and motor regions, whereas higher assignments capture regions within the default mode network. Across the 5 cyto-architectonic classes, SFC gradually decreased from granular (typically capturing sensory regions) to agranular (typically capturing motor and association regions) types and displayed its lowest value in the polar cortical type (**Supplemental Figure 7B**; **Supplemental Table 16**). Similarly, we observed a significant negative correlation between a brain region's SFC and its assigned location along the BigBrain gradient of microstructure profile covariance (**Supplemental Figure 7D**; r=-0.31; p_{spin}=0.039); primary sensory regions occupy the lower end of this gradient while limbic regions represent its apex.

We next computed each brain region's average temporal SFC variance across subjects and examined its heterogeneous expression along the unimodal (sensory)-transmodal (association) hierarchy. In contrast to SFC, temporal SFC variance was highest in the limbic system (**Supplemental Figure 8A**; **Supplemental Table 17**); a pronounced increase in temporal SFC variance was observed along the unimodal-transmodal hierarchy, as captured by the principal gradient (**Supplemental Figure 8C**; r=0.31; p_{spin}=0.047). Using cyto-architectonic annotations, temporal SFC variance (unlike SFC itself) was highest in the polar cortical type; the remaining 4 cortical types displayed similar degrees of temporal SFC variance (**Supplemental Figure 8B**; **Supplemental Table 18**). Under the more continuous BigBrain gradient, we observed a significant positive correlation between a brain region's temporal SFC variance and its assigned location along the gradient (**Supplemental Figure 8D**; r=0.46; p_{spin}=0.024). As with the Schaefer atlas, in order to ensure that the correlations observed between a brain region's temporal SFC variance and its location in the sensory-association hierarchy (as shown in **Supplementary Figures 8C** and **8D**) were not confounded by the presence of any outlier regions, we repeated the aforementioned analyses. After identifying the outlier regions (n=9), we excluded them and repeated the analyses reported in these figures. Both correlations remained significant (temporal SFC variance vs. principal functional gradient: r=0.29; p_{spin}=0.049, and temporal SFC variance vs. BigBrain gradient: r=0.44; p_{spin}=0.027)—same as when including the outliers in the analysis.

Biological Correlates of Structure-Function Coupling: Whole-brain perspective

Across the 360 brain regions defined by the HCP multi-modal cortical parcellation, we observed a significant positive correlation between SFC and intracortical myelin content (**Supplemental Figure 9A**; r=0.53; p_{spin}<10^{-4}), and a negative, albeit non-significant, correlation between temporal SFC variance and intracortical myelin content (**Supplemental Figure 9B**; r=-0.19; p_{spin}=0.169). Higher SFC values corresponded to larger Hurst exponents and thus a decreased excitation-inhibition (EI) ratio (**Supplemental Figure 9C**; r=0.33; p_{spin}=0.018), whereas higher temporal variance in SFC corresponded to lower Hurst exponents and thus a heightened EI-ratio (**Supplemental Figure 9D**; r=-0.63; p_{spin}<10^{-4}).

To ensure that the association between a region's SFC and either biological marker was independent of the other marker and also independent from that region's position along the cortical

hierarchy, we re-examined the above relationships using multiple linear regression models. We found that SFC (dependent variable) was independently and positively correlated with intracortical myelin content (β_{stand}=0.402; 95% non-parametric bootstrap confidence interval [*BCI*]=[0.404, 0.406]; $p<10^{-4}$; variance inflation factor [*VIF*]=1.46) and with the Hurst exponent (β_{stand}=0.323; 95% *BCI*=[0.323, 0.325]; $p<10^{-4}$; *VIF*=1.30), after adjusting for the other biological marker, the interaction effect between intracortical myelination and the Hurst exponent, as well as the principal gradient and BigBrain scalar assignments. Further, the correspondence between temporal SFC variance (dependent variable) and both intracortical myelin content (β_{stand}=0.110; 95% *BCI*=[0.111, 0.113]; p=0.002; *VIF*=1.46) and the Hurst exponent (β_{stand}=-0.610; 95% *BCI*=[-0.611, -0.609]; $p<10^{-4}$; *VIF*=1.30) remained significant after adjusting for the other marker, the interaction effect between intracortical myelination and the Hurst exponent, and the principal gradient and BigBrain scalar assignments. Notably, the interaction effect between intracortical myelination and the Hurst exponent was significant within this model (β_{stand}=0.171; 95% *BCI*=[0.169, 0.170]; $p=2\text{x}10^{-4}$; *VIF*=1.09); a potential causal relationship between temporal SFC variance, intracortical myelination, and the Hurst exponent was further explored via a mediation model. Similarly to the atlas-based analysis reported in the manuscript, the Hurst exponent was found to significantly mediate the correlation between intracortical myelination and temporal SFC variance (total effect=-0.003; $p=10^{-4}$, indirect effect=-0.0015; *BCI*=[-0.0027, -0.0004]). In other words, the Hurst exponent (i.e., EI-ratio) accounted for 50% of the correlation between intracortical myelination and temporal SFC variance.

Biological Correlates of Structure-Function Coupling: Regional perspective

As with the atlas-based analysis reported in the manuscript, we begin with the cyto-architectonic class that displayed the overall highest SFC: the granular type. We observed a significant positive association between SFC (dependent variable) and the Hurst exponent but not with intracortical myelin content, after adjusting for the effects of the other biological marker (**Supplemental Table 19A**). In the parietal and frontal types, we observed a significant positive association between SFC and the Hurst exponent as well as the intracortical myelin content (**Supplemental Table 19A**). Within the agranular cyto-architectonic class, we observed that SFC was positively correlated only with intracortical myelin content but not with the Hurst exponent, within the same regression model (**Supplemental Table 19A**). Taking these results together, we once again notice a distinct pattern as we transition from granular to agranular cortical regions: a gradual shift from the Hurst exponent to intracortical myelin content as being the principal predictor of SFC (as supported by the numerical changes in the standardized β and false discovery rate-adjusted p-values: **Supplemental Table 19A**; **Figure 7**). As with the atlas-based analysis using the Schaefer 400 parcellation reported in the manuscript and in Supplemental Analyses 1 and 2A above, the polar cortical regions were once again an exception to this rule (**Supplemental Table 19A**).

Interestingly—and in agreement with the atlas-based results reported in the manuscript as well as Supplemental Analyses 1 and 2A—dynamic regulation of temporal SFC variance (as opposed to SFC itself) was more persistently dependent upon the Hurst exponent, across the cyto-architectonic classes. Specifically, temporal SFC variance independently correlated only with the Hurst exponent across all cortical types, after adjusting for the effects of intracortical myelin content (**Supplemental Table 19B**).

SUPPLEMENTAL ANALYSIS 3:

Lastly, in addition to processing the subjects from our Penn sample (*n*=14) using our voxel-based methodology reported in the manuscript, we also analyzed them using the coarser atlas-based Schaefer cortical parcellation (400 brain regions). We report the results below.

Structure-Function Coupling Variations along the Cortical Hierarchy

In the 7 resting-state systems, SFC was highest in the primary visual and somatomotor cortices, intermediate in the ventral attention and limbic systems, and lowest in the dorsal attention, fronto-parietal, and default mode systems (**Supplemental Figure 10A**; **Supplemental Table 20**). A decrease in SFC along the unimodal-transmodal hierarchy was also evident along the principal functional gradient, in the form of a significant negative correlation between a brain region's SFC and its assigned principal gradient scalar (**Supplemental Figure 10C**; r=-0.19; p_{spin}=0.014); lower assignments within this gradient capture primary sensory and motor regions, whereas higher assignments capture regions within the default mode network. Across the 5 cyto-architectonic classes, SFC gradually decreased from granular (typically capturing sensory regions) to agranular (typically capturing motor and association regions) types (**Supplemental Figure 10B**; **Supplemental Table 21**). Similarly, we observed a negative, albeit non-significant, correlation between a brain region's SFC and its assigned location along the BigBrain gradient of microstructure profile covariance (**Supplemental Figure 10D**; r=-0.04; p_{spin}=0.362); primary sensory regions occupy the lower end of this gradient while limbic regions represent its apex.

We next computed each brain region's average temporal SFC variance across subjects and examined its heterogeneous expression along the unimodal (sensory)-transmodal (association) hierarchy. In contrast to SFC, temporal SFC variance was highest in the fronto-parietal and limbic systems, and lowest in the primary visual cortex (**Supplemental Figure 11A**; **Supplemental Table 22**); a pronounced increase in temporal SFC variance was observed along the unimodal-transmodal hierarchy, as captured by the principal gradient (**Supplemental Figure 11C**; r=0.22; p_{spin}=0.030). Using cyto-architectonic annotations, temporal SFC variance was highest in the agranular cortex and lowest in the parietal and granular cortices (**Supplemental Figure 11B**; **Supplemental Table 23**). Under the more continuous BigBrain gradient, we observed a positive correlation between a brain region's temporal SFC variance and its assigned location along the gradient (**Supplemental Figure 11D**; r=0.21; p_{spin}=0.082). Similar to Supplemental Analyses 1 and 2, we wanted to ensure that the correlations observed between a brain region's temporal SFC variance and its location in the sensory-association hierarchy (as shown in **Supplemental Figures 11C** and **11D**) were not confounded by the presence of any outlier regions. After identifying the outlier regions (n=10), we excluded them and repeated the analyses reported in these figures. Both correlations remained qualitatively the same as when the outliers were included: the association between temporal SFC variance and principal functional gradient remained significant (r=0.22; p_{spin}=0.047), whereas the association between temporal SFC variance and the BigBrain gradient remained insignificant (r=0.21; p_{spin}=0.084).

Biological Correlates of Structure-Function Coupling: Whole-brain perspective

Across the 400 brain regions defined by the Schaefer cortical parcellation, we observed a significant positive correlation between SFC and intracortical myelin content (**Supplemental Figure 12A**; r=0.21; p_{spin}=0.003), and a significant negative correlation between temporal SFC variance and intracortical myelin content (**Supplemental Figure 12B**; r=-0.44; $p_{spin}<10^{-4}$). Higher SFC values corresponded to larger Hurst exponents and thus a decreased excitation-inhibition (EI) ratio (**Supplemental Figure 12C**; r=0.11; p_{spin}=0.090), whereas higher temporal variance in SFC corresponded to lower Hurst exponents and thus a heightened EI-ratio (**Supplemental Figure 12D**; r=-0.22; p_{spin}=0.037).

To ensure that the association between a region's SFC and either biological marker was independent of the other marker and also independent from that region's position along the cortical hierarchy, we re-examined the above relationships using multiple linear regression models. We found that SFC (dependent variable) was independently and positively correlated with intracortical myelin content (β_{stand}=0.230; 95% non-parametric bootstrap confidence interval [*BCI*]=[0.230, 0.233]; $p<10^{-4}$; variance inflation factor [*VIF*]=1.35) and the Hurst exponent (β_{stand}=0.110; 95% *BCI*=[0.110, 0.111]; p=0.018; *VIF*=1.29), after adjusting for the other biological marker, the interaction effect between intracortical myelination and the Hurst exponent, as well as the principal gradient and BigBrain scalar assignments. Further, the correspondence between temporal SFC variance (dependent variable) and both the intracortical myelin content (β_{stand}=-0.341; 95% *BCI*=[-0.343, -0.341]; $p<10^{-4}$; *VIF*=1.35) and the Hurst exponent (β_{stand}=-0.175; 95% *BCI*=[-0.176, -0.174]; $p=2\text{x}10^{-4}$; *VIF*=1.29) remained significant after adjusting for the other marker, the interaction effect between intracortical myelination and the Hurst exponent, and the principal gradient and BigBrain scalar assignments. In contrast to the atlas-based analysis reported in the manuscript, the interaction effect between intracortical myelin content and the Hurst exponent was not significant in this model (p=0.49), and the Hurst exponent was not found to mediate the correlation between intracortical myelination and temporal SFC variance (total effect=-0.161; indirect effect=0.0015; *BCI*=[-0.006, 0.011]); these findings could be attributed to the Penn sample's significantly smaller sample size.

<u>Biological Correlates of Structure-Function Coupling: Regional perspective</u>

We begin with the cyto-architectonic class that displayed the highest SFC: the granular type. We observed a trend towards a positive association between SFC (dependent variable) and the Hurst exponent but not with intracortical myelin content, after adjusting for the effects of the other biological marker (**Supplemental Table 24A**). In the parietal type, we observed a significant positive association between SFC and the Hurst exponent as well as the intracortical myelin content (**Supplemental Table 24A**). Within the agranular cyto-architectonic class, we observed that SFC was positively correlated only with intracortical myelin content but not with the Hurst exponent, within the same regression model (**Supplemental Table 24A**). Generally, we notice once again this trend where intracortical myelin content—rather than the Hurst exponent—correlates with SFC in the less granular layers; as we traverse from agranular to granular cortical layers, however, the effect size of the Hurst exponent in predicting SFC increases (**Supplemental Table 24A**).

Lastly, the dynamic regulation of temporal SFC variance appeared to be dependent upon both intracortical myelin content and the Hurst exponent, across most cyto-architectonic classes

(**Supplemental Table 24B**); this finding could point towards the concerted interaction between the two variables in determining the extent of SFC fluctuations across time.

METHODOLOGICAL CONSIDERATIONS AND STUDY LIMITATIONS

Several methodological considerations and limitations are pertinent to our work. First, in order to quantify the overall degree to which SFC fluctuates over time (i.e., throughout the duration of the functional scan), we used the statistical metric of *variance* (see **Methods: Structure-Function Coupling**). Although other metrics for variability such as standard deviation or coefficient of variation could have been used to assess temporal SFC fluctuations over time, we chose variance because it is mathematically related to the standard deviation (i.e., its squared value), does not get adjusted by the mean of the data (like the coefficient of variation), and accounts for the total number of data points considered (**Equation 1**). Moreover, our choice to measure the temporal SFC variance by splitting the duration of the functional scan into 20 continuous temporal windows served the purpose of generating time windows that captured a few minutes of functional activity. Given the differences in functional scan duration and repetition time across the two participant groups (Human Connectome Project [HCP] and Penn samples) and across our processing pipelines, each of the 20 time windows corresponded to ~40 seconds of functional activity in the atlas-based analyses (HCP sample) described in the main manuscript and Supplemental Analysis 1, ~3 minutes of functional activity in the atlas-based analyses (HCP sample) described in Supplemental Analyses 2A and 2B, and 1 minute of functional activity in the atlas- and voxel-based analyses derived from analyzing the Penn sample (Supplemental Analysis 3 and main manuscript). Even though the duration of each time window could be expected to influence the corresponding temporal SFC variance, our results remained largely consistent across all analyses utilizing varying window durations. Future studies, however, should further vary the number of time windows (with or without temporal overlap) and examine whether—and to what extent—this choice impacts the resulting SFC variability over time.

Another important methodological consideration was the choice of metrics used to non-invasively assess the biological substrates of intracortical myelination and EI-ratio. For the former, we used the previously validated T1-weighted/T2-weighted signal intensity ratio approach (see **Methods: Intracortical Myelination**), as it accurately detects myelo-architectonic boundaries, is a good proxy of myelin concentration, yields a high signal-to-noise ratio, and has high test-retest reliability.[1–7] Recent studies, however, have suggested that the T1-weighted/T2-weighted ratio captures not only myelination but also inflammation and iron accumulation—an aspect that becomes particularly relevant when examining myelination levels within the subcortical gray matter regions.[2,6,8,9] Here, however, we only analyzed cortical regions; moreover, both participant groups consisted of healthy young adults where pathological or aberrant levels of microstructural markers (e.g., iron levels, inflammation, edemas, atrophy) would not be expected. Nonetheless, future studies could apply other proposed methods to non-invasively quantify myelin content (e.g., magnetization transfer, simultaneous tissue relaxometry, etc.)[8–10] and examine how the relation between the estimated myelin content and SFC varies.

Furthermore, we used a recently published approach to non-invasively quantify the balance between synaptic excitation and inhibition across the human cortex, using the Hurst exponent of

the resting-state blood oxygen level-dependent (BOLD) signal time-series.[11] Building upon prior work,[12] this recent study demonstrated *in silico* and *in vivo* that changes in the Hurst exponent of the functional BOLD signal time-series reflect shifts in the synaptic EI-ratio.[11] We do acknowledge, however, the inherent limitations of these measurements: namely, changes in synaptic excitation and inhibition occur in timescales significantly smaller than the ones captured by the resting-state functional scans acquired. In addition, using the Hurst exponent of the signal time-series is an indirect assessment of the macroscale EI-ratio; a more direct—while still non-invasive—approach, for instance, could involve tracking the dynamical release and re-uptake of glutamatergic and GABA(Gamma-aminobutyric acid)-ergic neurotransmitters using pharmacological (functional) magnetic resonance imaging.[13]

Regarding our results, it should be noted that there was one main distinction between our atlas- and voxel-based results discussed in section: 'Biological Correlates of Structure-Function Coupling: Regional perspective,' pertaining to the relationship between SFC and the biological substrates of interest within the polar cyto-architectonic class. More specifically, within the atlas-based analyses, there was no significant association between SFC and intracortical myelination when using the Schaefer 400 atlas, and a significant association between SFC and intracortical myelination but of the opposite directionality (i.e., a negative standardized β coefficient) when using the HCP multi-modal atlas. The association between SFC and the Hurst exponent was non-significant in either atlas-based analysis, within the polar cortical class. In contrast, within the voxel-based analyses, there was a significant association between SFC and both independent variables across the polar cortical regions; further, that correlation fit the broader pattern discussed in our Results and Discussion sections pointing to a gradual transition from the Hurst exponent to the intracortical myelination as the principal predictor of SFC, as we traverse from granular to progressively less granular cortical regions. This discrepancy between the atlas- and voxel-based analyses could be attributed to the relatively small number of cortical regions classified as 'polar' in the atlas-based analyses (23/400 cortical regions in the Schaefer 400 atlas and 23/360 cortical regions in the HCP multi-modal atlas). This difference could result in limited statistical power—especially given that multiple linear regression models were invoked—to capture the underlying dynamics. The potential issue of statistical power was overcome, however, by our voxel-based analyses wherein thousands of brain regions per subject (i.e., cortical voxels) were classified as 'polar.'

Lastly, even though the goal of this study was to identify the biological substrates that mediate how strongly coupled the functional connectivity is to the structural connectivity, we do acknowledge that—in addition to intracortical myelination and EI-ratio—there could be a number of other biological markers that could contribute towards this coupling. Specific examples could include (i) cyto-architectonic properties, such as the underlying neuronal density, neuronal size, and firing behavior (e.g., tonic versus burst firing) patterns found in different brain regions, and (ii) other neuromodulatory properties, such as the contribution of various neurotransmitters and neuropeptides, and their heterogeneous effects on different brain regions.

Equation 1

$$Temporal\ SFC\ Variance = \frac{1}{N-1}\sum_{i=1}^{N}|x_i - \mu|^2 \quad (1)$$

where N is the total number of temporal windows used in the analysis (here, $N = 20$), x_i is the structure-function coupling at temporal window i, and μ the mean structure-function coupling across the N temporal windows.

TABLES

Supplemental Table 1

Multiple Comparisons

Dependent Variable: Structure-Function Coupling

Test: Tamhane

(I) Resting-state functional networks	(J) Resting-state functional networks	Mean Difference (I-J)	Std. Error	Sig.	95% Confidence Interval	
					Lower Bound	Upper Bound
Visual	Somatomotor	.013437154257	.010935000532	.995	-.02034806770	.04722237621
	Dorsal Attention	.076266028035*	.013452090181	<.001	.03425349044	.11827856563
	Ventral Attention	.113438364384*	.011973840778	<.001	.07618490991	.15069181886
	Limbic	.144780830272*	.014886094533	<.001	.09697103687	.19259062368
	Fronto-parietal	.088606331042*	.012236231460	<.001	.05058990642	.12662275566
	Default Mode	.064906919745*	.009751748353	<.001	.03471470277	.09509913672
Somatomotor	Visual	-.013437154257	.010935000532	.995	-.04722237621	.02034806770
	Dorsal Attention	.062828873778*	.013352469165	<.001	.02114846810	.10450927946
	Ventral Attention	.100001210126*	.011861811226	<.001	.06314274281	.13685967745
	Limbic	.131343676015*	.014796131692	<.001	.08379937256	.17888797947
	Fronto-parietal	.075169176784*	.012126626197	<.001	.03753700762	.11280134595
	Default Mode	.051469765488*	.009613859710	<.001	.02182279699	.08111673398
Dorsal Attention	Visual	-.076266028035*	.013452090181	<.001	-.11827856563	-.03425349044
	Somatomotor	-.062828873778*	.013352469165	<.001	-.10450927946	-.02114846810
	Ventral Attention	.037172336349	.014215732804	.199	-.00719833189	.08154300458
	Limbic	.068514802237*	.016742461188	.003	.01546224091	.12156736356
	Fronto-parietal	.012340303007	.014437435940	1.000	-.03266045089	.05734105691
	Default Mode	-.011359108290	.012402047900	1.000	-.05033464293	.02761642635
Ventral Attention	Visual	-.113438364384*	.011973840778	<.001	-.15069181886	-.07618490991
	Somatomotor	-.100001210126*	.011861811226	<.001	-.13685967745	-.06314274281
	Dorsal Attention	-.037172336349	.014215732804	.199	-.08154300458	.00719833189
	Limbic	.031342465889	.015579606522	.657	-.01839391985	.08107885163

	Fronto-parietal	-.024832033342	.013071101303	.730	-.06551284090	.01584877422
	Default Mode	-.048531444639*	.010780766432	<.001	-.08221779017	-.01484509911
Limbic	Visual	-.144780830272*	.014886094533	<.001	-.19259062368	-.09697103687
	Somatomotor	-.131343676015*	.014796131692	<.001	-.17888797947	-.08379937256
	Dorsal Attention	-.068514802237*	.016742461188	.003	-.12156736356	-.01546224091
	Ventral Attention	-.031342465889	.015579606522	.657	-.08107885163	.01839391985
	Fronto-parietal	-.056174499231*	.015782161980	.017	-.10643794502	-.00591105344
	Default Mode	-.079873910527*	.013944456689	<.001	-.12530544522	-.03444237584
Fronto-parietal	Visual	-.088606331042*	.012236231460	<.001	-.12662275566	-.05058990642
	Somatomotor	-.075169176784*	.012126626197	<.001	-.11280134595	-.03753700762
	Dorsal Attention	-.012340303007	.014437435940	1.000	-.05734105691	.03266045089
	Ventral Attention	.024832033342	.013071101303	.730	-.01584877422	.06551284090
	Limbic	.056174499231*	.015782161980	.017	.00591105344	.10643794502
	Default Mode	-.023699411297	.011071468838	.527	-.05822753236	.01082870976
Default Mode	Visual	-.064906919745*	.009751748353	<.001	-.09509913672	-.03471470277
	Somatomotor	-.051469765488*	.009613859710	<.001	-.08111673398	-.02182279699
	Dorsal Attention	.011359108290	.012402047900	1.000	-.02761642635	.05033464293
	Ventral Attention	.048531444639*	.010780766432	<.001	.01484509911	.08221779017
	Limbic	.079873910527*	.013944456689	<.001	.03444237584	.12530544522
	Fronto-parietal	.023699411297	.011071468838	.527	-.01082870976	.05822753236

*. The mean difference is significant at the 0.05 level.

Supplemental Table 1: Mean differences in structure-function coupling across the 7 resting-state functional networks. One-way ANOVA table with post-hoc correction for multiple comparisons (Tamhane's T2 – equal variances not assumed): Dependent variable: Structure-function coupling; factor: Resting-state functional networks. Data derived from the 100 unrelated HCP subjects, analyzed using the Schaefer 400 atlas.

Supplemental Table 2

Multiple Comparisons

Dependent Variable: Structure-Function Coupling

Test: Tamhane

(I) Cyto-architectonic classes	(J) Cyto-architectonic classes	Mean Difference (I-J)	Std. Error	Sig.	95% Confidence Interval	
					Lower Bound	Upper Bound
Agranular	Frontal	-.040037415719*	.009367735623	<.001	-.06665855260	-.01341627884
	Parietal	-.069324213535*	.010774866835	<.001	-.09990211603	-.03874631104
	Polar	.022819416864	.018708818333	.928	-.03344397781	.07908281153
	Granular	-.113045932479*	.014725716997	<.001	-.15700703941	-.06908482555

Frontal	Agranular	.040037415719*	.009367735623	<.001	.01341627884	.06665855260
	Parietal	-.029286797816*	.009078052342	.015	-.05502268557	-.00355091006
	Polar	.062856832583*	.017785757291	.015	.00852894352	.11718472165
	Granular	-.073008516760*	.013533662479	<.001	-.11437762486	-.03163940866
Parietal	Agranular	.069324213535*	.010774866835	<.001	.03874631104	.09990211603
	Frontal	.029286797816*	.009078052342	.015	.00355091006	.05502268557
	Polar	.092143630399*	.018565463825	<.001	.03619740163	.14808985917
	Granular	-.043721718944*	.014543153190	.048	-.08725419599	-.00018924190
Polar	Agranular	-.022819416864	.018708818333	.928	-.07908281153	.03344397781
	Frontal	-.062856832583*	.017785757291	.015	-.11718472165	-.00852894352
	Parietal	-.092143630399*	.018565463825	<.001	-.14808985917	-.03619740163
	Granular	-.135865349343*	.021105104425	<.001	-.19848265117	-.07324804752
Granular	Agranular	.113045932479*	.014725716997	<.001	.06908482555	.15700703941
	Frontal	.073008516760*	.013533662479	<.001	.03163940866	.11437762486
	Parietal	.043721718944*	.014543153190	.048	.00018924190	.08725419599
	Polar	.135865349343*	.021105104425	<.001	.07324804752	.19848265117

*. The mean difference is significant at the 0.05 level.

Supplemental Table 2: Mean differences in structure-function coupling across the 5 von-Economo/Koskinas-inspired cyto-architectonic classes. One-way ANOVA table with post-hoc correction for multiple comparisons (Tamhane's T2 – equal variances not assumed): Dependent variable: Structure-function coupling; factor: Cyto-architectonic classes. Data derived from the 100 unrelated HCP subjects, analyzed using the Schaefer 400 atlas.

Supplemental Table 3

Multiple Comparisons

Dependent Variable: Temporal Structure-Function Coupling Variance

Test: Tamhane

(I) Resting-state functional networks	(J) Resting-state functional networks	Mean Difference (I-J)	Std. Error	Sig.	95% Confidence Interval	
					Lower Bound	Upper Bound
Visual	Somatomotor	-.000346001683	.000182343952	.730	-.00091203094	.00022002758
	Dorsal Attention	-.000460281011	.000215938467	.531	-.00113133779	.00021077577
	Ventral Attention	-.000611574354	.000234505602	.199	-.00134127354	.00011812483
	Limbic	-.003954179137*	.000570500319	<.001	-.00584861356	-.00205974471
	Fronto-parietal	-.001053082598*	.000210402807	<.001	-.00170571752	-.00040044768
	Default Mode	-.001337766725*	.000213111053	<.001	-.00199536470	-.00068016875
Somatomotor	Visual	.000346001683	.000182343952	.730	-.00022002758	.00091203094
	Dorsal Attention	-.000114279329	.000183039479	1.000	-.00068655954	.00045800088
	Ventral Attention	-.000265572671	.000204614044	.990	-.00090738489	.00037623955

	Limbic	-.003608177454*	.000558878022	<.001	-.00547879930	-.00173755561
	Fronto-parietal	-.000707080916*	.000176474844	.003	-.00125638535	-.00015777648
	Default Mode	-.000991765042*	.000179695159	<.001	-.00154558841	-.00043794167
Dorsal Attention	Visual	.000460281011	.000215938467	.531	-.00021077577	.00113133779
	Somatomotor	.000114279329	.000183039479	1.000	-.00045800088	.00068655954
	Ventral Attention	-.000151293342	.000235046828	1.000	-.00088452883	.00058194214
	Limbic	-.003493898125*	.000570723004	<.001	-.00538895108	-.00159884517
	Fronto-parietal	-.000592801587	.000211005865	.119	-.00124992760	.00006432442
	Default Mode	-.000877485713*	.000213706468	.002	-.00153945316	-.00021551827
Ventral Attention	Visual	.000611574354	.000234505602	.199	-.00011812483	.00134127354
	Somatomotor	.000265572671	.000204614044	.990	-.00037623955	.00090738489
	Dorsal Attention	.000151293342	.000235046828	1.000	-.00058194214	.00088452883
	Limbic	-.003342604783*	.000578003637	<.001	-.00525334945	-.00143186011
	Fronto-parietal	-.000441508245	.000229971588	.715	-.00115848538	.00027546889
	Default Mode	-.000726192371*	.000232451954	.047	-.00144795656	-.00000442818
Limbic	Visual	.003954179137*	.000570500319	<.001	.00205974471	.00584861356
	Somatomotor	.003608177454*	.000558878022	<.001	.00173755561	.00547879930
	Dorsal Attention	.003493898125*	.000570723004	<.001	.00159884517	.00538895108
	Ventral Attention	.003342604783*	.000578003637	<.001	.00143186011	.00525334945
	Fronto-parietal	.002901096538*	.000568651622	<.001	.00101047472	.00479171835
	Default Mode	.002616412412*	.000569659238	.002	.00072388126	.00450894356
Fronto-parietal	Visual	.001053082598*	.000210402807	<.001	.00040044768	.00170571752
	Somatomotor	.000707080916*	.000176474844	.003	.00015777648	.00125638535
	Dorsal Attention	.000592801587	.000211005865	.119	-.00006432442	.00124992760
	Ventral Attention	.000441508245	.000229971588	.715	-.00027546889	.00115848538
	Limbic	-.002901096538*	.000568651622	<.001	-.00479171835	-.00101047472
	Default Mode	-.000284684126	.000208111447	.982	-.00092779613	.00035842788
Default Mode	Visual	.001337766725*	.000213111053	<.001	.00068016875	.00199536470
	Somatomotor	.000991765042*	.000179695159	<.001	.00043794167	.00154558841
	Dorsal Attention	.000877485713*	.000213706468	.002	.00021551827	.00153945316
	Ventral Attention	.000726192371*	.000232451954	.047	.00000442818	.00144795656
	Limbic	-.002616412412*	.000569659238	.002	-.00450894356	-.00072388126
	Fronto-parietal	.000284684126	.000208111447	.982	-.00035842788	.00092779613

*. The mean difference is significant at the 0.05 level.

Supplemental Table 3: Mean differences in temporal structure-function coupling variance across the 7 resting-state functional networks. One-way ANOVA table with post-hoc correction for multiple comparisons (Tamhane's T2 – equal variances not assumed): Dependent variable: Temporal structure-function coupling variance; factor:

Resting-state functional networks. Data derived from the 100 unrelated HCP subjects, analyzed using the Schaefer 400 atlas.

Supplemental Table 4

Multiple Comparisons

Dependent Variable: Temporal Structure-Function Coupling Variance

Test: Tamhane

(I) Cyto-architectonic classes	(J) Cyto-architectonic classes	Mean Difference (I-J)	Std. Error	Sig.	95% Confidence Interval Lower Bound	95% Confidence Interval Upper Bound
Agranular	Frontal	-.000352641780	.000167603665	.316	-.00082980057	.00012451701
	Parietal	.000267611133	.000192783665	.839	-.00027967034	.00081489261
	Polar	-.003862034080*	.000611605373	<.001	-.00574137685	-.00198269131
	Granular	-.000051966217	.000284881892	1.000	-.00090557720	.00080164476
Frontal	Agranular	.000352641780	.000167603665	.316	-.00012451701	.00082980057
	Parietal	.000620252914*	.000153213021	<.001	.00018565300	.00105485283
	Polar	-.003509392299*	.000600308105	<.001	-.00536772851	-.00165105609
	Granular	.000300675564	.000259742912	.950	-.00050032165	.00110167278
Parietal	Agranular	-.000267611133	.000192783665	.839	-.00081489261	.00027967034
	Frontal	-.000620252914*	.000153213021	<.001	-.00105485283	-.00018565300
	Polar	-.004129645213*	.000607819360	<.001	-.00600181470	-.00225747572
	Granular	-.000319577350	.000276660322	.949	-.00115489925	.00051574455
Polar	Agranular	.003862034080*	.000611605373	<.001	.00198269131	.00574137685
	Frontal	.003509392299*	.000600308105	<.001	.00165105609	.00536772851
	Parietal	.004129645213*	.000607819360	<.001	.00225747572	.00600181470
	Granular	.003810067863*	.000642990299	<.001	.00186342655	.00575670917
Granular	Agranular	.000051966217	.000284881892	1.000	-.00080164476	.00090557720
	Frontal	-.000300675564	.000259742912	.950	-.00110167278	.00050032165
	Parietal	.000319577350	.000276660322	.949	-.00051574455	.00115489925
	Polar	-.003810067863*	.000642990299	<.001	-.00575670917	-.00186342655

*. The mean difference is significant at the 0.05 level.

Supplemental Table 4: Mean differences in temporal structure-function coupling variance across the 5 von-Economo/Koskinas-inspired cyto-architectonic classes. One-way ANOVA table with post-hoc correction for multiple comparisons (Tamhane's T2 – equal variances not assumed): Dependent variable: Temporal structure-function coupling variance; factor: Cyto-architectonic classes. Data derived from the 100 unrelated HCP subjects, analyzed using the Schaefer 400 atlas.

Supplemental Table 5

Multiple Comparisons

Dependent Variable: Structure-Function Coupling

Test: Tamhane

(I) Resting-state functional networks	(J) Resting-state functional networks	Mean Difference (I-J)	Std. Error	Sig.	95% Confidence Interval Lower Bound	95% Confidence Interval Upper Bound
Visual	Somatomotor	.009107576726	.011230708177	1.000	-.02573808010	.04395323355
	Dorsal Attention	.086044397800*	.013397380051	<.001	.04424797363	.12784082197
	Ventral Attention	.111751436338*	.010984567983	.000	.07763090343	.14587196924
	Limbic	.180775407291*	.016196657279	<.001	.12881725874	.23273355584
	Fronto-parietal	.095090988743*	.013071994472	<.001	.05435825706	.13582372043
	Default Mode	.082620982778*	.010883044118	<.001	.04894521263	.11629675293
Somatomotor	Visual	-.009107576726	.011230708177	1.000	-.04395323355	.02573808010
	Dorsal Attention	.076936821074*	.012828046023	<.001	.03679531009	.11707833206
	Ventral Attention	.102643859612*	.010282495141	<.001	.07065946383	.13462825539
	Limbic	.171667830565*	.015728975795	<.001	.12090140788	.22243425326
	Fronto-parietal	.085983412017*	.012487834561	<.001	.04696119716	.12500562687
	Default Mode	.073513406052*	.010173967850	<.001	.04202814245	.10499866965
Dorsal Attention	Visual	-.086044397800*	.013397380051	<.001	-.12784082197	-.04424797363
	Somatomotor	-.076936821074*	.012828046023	<.001	-.11707833206	-.03679531009
	Ventral Attention	.025707038538	.012613115887	.620	-.01382703794	.06524111502
	Limbic	.094731009492*	.017342481530	<.001	.03955536726	.14990665172
	Fronto-parietal	.009046590943	.014467446150	1.000	-.03612277475	.05421595664
	Default Mode	-.003423415022	.012524799714	1.000	-.04261113705	.03576430701
Ventral Attention	Visual	-.111751436338*	.010984567983	.000	-.14587196924	-.07763090343
	Somatomotor	-.102643859612*	.010282495141	<.001	-.13462825539	-.07065946383
	Dorsal Attention	-.025707038538	.012613115887	.620	-.06524111502	.01382703794
	Limbic	.069023970954*	.015554182948	.001	.01868704144	.11936090047
	Fronto-parietal	-.016660447594	.012266945001	.984	-.05505389272	.02173299753
	Default Mode	-.029130453560	.009901593278	.079	-.05979745603	.00153654891
Limbic	Visual	-.180775407291*	.016196657279	<.001	-.23273355584	-.12881725874
	Somatomotor	-.171667830565*	.015728975795	<.001	-.22243425326	-.12090140788
	Dorsal Attention	-.094731009492*	.017342481530	<.001	-.14990665172	-.03955536726
	Ventral Attention	-.069023970954*	.015554182948	.001	-.11936090047	-.01868704144
	Fronto-parietal	-.085684418548*	.017092364169	<.001	-.14014578493	-.03122305217
	Default Mode	-.098154424513*	.015482652314	<.001	-.14826223139	-.04804661764
Fronto-parietal	Visual	-.095090988743*	.013071994472	<.001	-.13582372043	-.05435825706
	Somatomotor	-.085983412017*	.012487834561	<.001	-.12500562687	-.04696119716

	Dorsal Attention	-.009046590943	.014467446150	1.000	-.05421595664	.03612277475
	Ventral Attention	.016660447594	.012266945001	.984	-.02173299753	.05505389272
	Limbic	.085684418548*	.017092364169	<.001	.03122305217	.14014578493
	Default Mode	-.012470005965	.012176118230	1.000	-.05049979794	.02555978601
Default Mode	Visual	-.082620982778*	.010883044118	<.001	-.11629675293	-.04894521263
	Somatomotor	-.073513406052*	.010173967850	<.001	-.10499866965	-.04202814245
	Dorsal Attention	.003423415022	.012524799714	1.000	-.03576430701	.04261113705
	Ventral Attention	.029130453560	.009901593278	.079	-.00153654891	.05979745603
	Limbic	.098154424513*	.015482652314	<.001	.04804661764	.14826223139
	Fronto-parietal	.012470005965	.012176118230	1.000	-.02555978601	.05049979794

*. The mean difference is significant at the 0.05 level.

Supplemental Table 5: Mean differences in structure-function coupling across the 7 resting-state functional networks. One-way ANOVA table with post-hoc correction for multiple comparisons (Tamhane's T2 – equal variances not assumed): Dependent variable: Structure-function coupling; factor: Resting-state functional networks. Data derived from the 100 unrelated HCP subjects, analyzed using the HCP multi-modal atlas.

Supplemental Table 6

Multiple Comparisons

Dependent Variable: Structure-Function Coupling

Test: Tamhane

(I) Cyto-architectonic classes	(J) Cyto-architectonic classes	Mean Difference (I-J)	Std. Error	Sig.	95% Confidence Interval Lower Bound	95% Confidence Interval Upper Bound
Agranular	Frontal	-.030215803408*	.010382118814	.042	-.05984410357	-.00058750325
	Parietal	-.076467057724*	.012147499886	<.001	-.11099675238	-.04193736307
	Polar	.047520300764	.018918155080	.155	-.00897703122	.10401763275
	Granular	-.084260914284*	.019192452563	.001	-.14247940850	-.02604242007
Frontal	Agranular	.030215803408*	.010382118814	.042	.00058750325	.05984410357
	Parietal	-.046251254316*	.009925432749	<.001	-.07442092933	-.01808157930
	Polar	.077736104172*	.017574101781	.001	.02415386721	.13131834114
	Granular	-.054045110876	.017869042984	.061	-.10965396028	.00156373853
Parietal	Agranular	.076467057724*	.012147499886	<.001	.04193736307	.11099675238
	Frontal	.046251254316*	.009925432749	<.001	.01808157930	.07442092933
	Polar	.123987358488*	.018671433148	<.001	.06807016396	.17990455302
	Granular	-.007793856560	.018949302351	1.000	-.06548221715	.04989450403
Polar	Agranular	-.047520300764	.018918155080	.155	-.10401763275	.00897703122
	Frontal	-.077736104172*	.017574101781	.001	-.13131834114	-.02415386721
	Parietal	-.123987358488*	.018671433148	<.001	-.17990455302	-.06807016396

	Granular	-.131781215048*	.023862332194	<.001	-.20253196245	-.06103046765
Granular	Agranular	.084260914284*	.019192452563	.001	.02604242007	.14247940850
	Frontal	.054045110876	.017869042984	.061	-.00156373853	.10965396028
	Parietal	.007793856560	.018949302351	1.000	-.04989450403	.06548221715
	Polar	.131781215048*	.023862332194	<.001	.06103046765	.20253196245

*. The mean difference is significant at the 0.05 level.

Supplemental Table 6: Mean differences in structure-function coupling across the 5 von-Economo/Koskinas-inspired cyto-architectonic classes. One-way ANOVA table with post-hoc correction for multiple comparisons (Tamhane's T2 – equal variances not assumed): Dependent variable: Structure-function coupling; factor: Cyto-architectonic classes. Data derived from the 100 unrelated HCP subjects, analyzed using the HCP multi-modal atlas.

Supplemental Table 7

Multiple Comparisons

Dependent Variable: Temporal Structure-Function Coupling Variance

Test: Tamhane

(I) Resting-state functional networks	(J) Resting-state functional networks	Mean Difference (I-J)	Std. Error	Sig.	95% Confidence Interval Lower Bound	95% Confidence Interval Upper Bound
Visual	Somatomotor	-.000181527307	.000285953066	1.000	-.00106999004	.00070693543
	Dorsal Attention	-.000194390164	.000267296838	1.000	-.00102654309	.00063776276
	Ventral Attention	-.000669260306	.000269344466	.266	-.00150664247	.00016812185
	Limbic	-.003945006637*	.000627551576	<.001	-.00601663082	-.00187338245
	Fronto-parietal	-.001152118296*	.000257475436	<.001	-.00195283400	-.00035140259
	Default Mode	-.001857336619*	.000307249163	<.001	-.00280591357	-.00090875966
Somatomotor	Visual	.000181527307	.000285953066	1.000	-.00070693543	.00106999004
	Dorsal Attention	-.000012862857	.000295738165	1.000	-.00093380899	.00090808327
	Ventral Attention	-.000487732999	.000297590161	.901	-.00141347742	.00043801142
	Limbic	-.003763479330*	.000640183133	<.001	-.00586350459	-.00166345406
	Fronto-parietal	-.000970590989*	.000286892075	.022	-.00186398885	-.00007719313
	Default Mode	-.001675809311*	.000332288294	<.001	-.00270249307	-.00064912555
Dorsal Attention	Visual	.000194390164	.000267296838	1.000	-.00063776276	.00102654309
	Somatomotor	.000012862857	.000295738165	1.000	-.00090808327	.00093380899
	Ventral Attention	-.000474870142	.000279711186	.871	-.00134724578	.00039750550
	Limbic	-.003750616473*	.000632070318	<.001	-.00583251891	-.00166871404
	Fronto-parietal	-.000957728132*	.000268301148	.012	-.00179550525	-.00011995102
	Default Mode	-.001662946455*	.000316376286	<.001	-.00264171672	-.00068417619
Ventral Attention	Visual	.000669260306	.000269344466	.266	-.00016812185	.00150664247
	Somatomotor	.000487732999	.000297590161	.901	-.00043801142	.00141347742

	Dorsal Attention	.000474870142	.000279711186	.871	-.00039750550	.00134724578
	Limbic	-.003275746331*	.000632938961	<.001	-.00535948880	-.00119200386
	Fronto-parietal	-.000482857990	.000270341170	.816	-.00132578593	.00036006995
	Default Mode	-.001188076313*	.000318108151	.006	-.00217148662	-.00020466601
Limbic	Visual	.003945006637*	.000627551576	<.001	.00187338245	.00601663082
	Somatomotor	.003763479330*	.000640183133	<.001	.00166345406	.00586350459
	Dorsal Attention	.003750616473*	.000632070318	<.001	.00166871404	.00583251891
	Ventral Attention	.003275746331*	.000632938961	<.001	.00119200386	.00535948880
	Fronto-parietal	.002792888341*	.000627980006	.002	.00072010438	.00486567230
	Default Mode	.002087670019	.000649974873	.057	-.00003425157	.00420959160
Fronto-parietal	Visual	.001152118296*	.000257475436	<.001	.00035140259	.00195283400
	Somatomotor	.000970590989*	.000286892075	.022	.00007719313	.00186398885
	Dorsal Attention	.000957728132*	.000268301148	.012	.00011995102	.00179550525
	Ventral Attention	.000482857990	.000270341170	.816	-.00036006995	.00132578593
	Limbic	-.002792888341*	.000627980006	.002	-.00486567230	-.00072010438
	Default Mode	-.000705218322	.000308123279	.396	-.00165813552	.00024769887
Default Mode	Visual	.001857336619*	.000307249163	<.001	.00090875966	.00280591357
	Somatomotor	.001675809311*	.000332288294	<.001	.00064912555	.00270249307
	Dorsal Attention	.001662946455*	.000316376286	<.001	.00068417619	.00264171672
	Ventral Attention	.001188076313*	.000318108151	.006	.00020466601	.00217148662
	Limbic	-.002087670019	.000649974873	.057	-.00420959160	.00003425157
	Fronto-parietal	.000705218322	.000308123279	.396	-.00024769887	.00165813552

*. The mean difference is significant at the 0.05 level.

Supplemental Table 7: Mean differences in temporal structure-function coupling variance across the 7 resting-state functional networks. One-way ANOVA table with post-hoc correction for multiple comparisons (Tamhane's T2 – equal variances not assumed): Dependent variable: Temporal structure-function coupling variance; factor: Resting-state functional networks. Data derived from the 100 unrelated HCP subjects, analyzed using the HCP multi-modal atlas.

Supplemental Table 8

Multiple Comparisons

Dependent Variable: Temporal Structure-Function Coupling Variance

Test: Tamhane

(I) Cyto-architectonic classes	(J) Cyto-architectonic classes	Mean Difference (I-J)	Std. Error	Sig.	95% Confidence Interval Lower Bound	95% Confidence Interval Upper Bound
Agranular	Frontal	.000165235911	.000267496654	1.000	-.00059854874	.00092902057
	Parietal	.000676981514	.000276581826	.148	-.00011204710	.00146601012

	Polar	-.004328708186*	.000551685657	<.001	-.00598795923	-.00266945715
	Granular	.000469761938	.000515137830	.990	-.00109691157	.00203643544
Frontal	Agranular	-.000165235911	.000267496654	1.000	-.00092902057	.00059854874
	Parietal	.000511745602	.000205363784	.127	-.00006928745	.00109277865
	Polar	-.004493944097*	.000519647805	<.001	-.00608644297	-.00290144522
	Granular	.000304526026	.000480670116	1.000	-.00119546442	.00180451647
Parietal	Agranular	-.000676981514	.000276581826	.148	-.00146601012	.00011204710
	Frontal	-.000511745602	.000205363784	.127	-.00109277865	.00006928745
	Polar	-.005005689700*	.000524382387	<.001	-.00660755525	-.00340382415
	Granular	-.000207219576	.000485784734	1.000	-.00171635160	.00130191245
Polar	Agranular	.004328708186*	.000551685657	<.001	.00266945715	.00598795923
	Frontal	.004493944097*	.000519647805	<.001	.00290144522	.00608644297
	Parietal	.005005689700*	.000524382387	<.001	.00340382415	.00660755525
	Granular	.004798470124*	.000681062673	<.001	.00278077369	.00681616656
Granular	Agranular	-.000469761938	.000515137830	.990	-.00203643544	.00109691157
	Frontal	-.000304526026	.000480670116	1.000	-.00180451647	.00119546442
	Parietal	.000207219576	.000485784734	1.000	-.00130191245	.00171635160
	Polar	-.004798470124*	.000681062673	<.001	-.00681616656	-.00278077369

*. The mean difference is significant at the 0.05 level.

Supplemental Table 8: Mean differences in temporal structure-function coupling variance across the 5 von-Economo/Koskinas-inspired cyto-architectonic classes. One-way ANOVA table with post-hoc correction for multiple comparisons (Tamhane's T2 – equal variances not assumed): Dependent variable: Temporal structure-function coupling variance; factor: Cyto-architectonic classes. Data derived from the 100 unrelated HCP subjects, analyzed using the HCP multi-modal atlas.

Supplemental Table 9

**A. Structure-Function Coupling**							
	**Intracortical Myelin**			**Hurst Exponent**			
**Cortical Type**	β_{stand}	95% *BCI*	Bootstrapped *p*-value (FDR)	β_{stand}	95% *BCI*	Bootstrapped *p*-value (FDR)	*VIF*
Granular	0.349	[0.344, 0.352]	0.075	0.502	[0.516, 0.525]	0.013	1.01
Polar	-0.395	[-0.402, -0.397]	0.017	0.225	[0.206, 0.213]	0.239	1.04
Parietal	0.411	[0.417, 0.421]	$<10^{-4}$	0.385	[0.377, 0.380]	0.004	1.01

Frontal	0.404	[0.403, 0.407]	<10^{-4}	0.273	[0.269, 0.273]	0.006	1.08
Agranular	0.484	[0.472, 0.477]	0.003	0.017	[0.006, 0.011]	0.922	1.33
**B. Temporal Structure-Function Coupling Variance**							
	**Intracortical Myelin**			**Hurst Exponent**			
**Cortical Type**	β_{stand}	95% *BCI*	Bootstrapped *p*-value (FDR)	β_{stand}	95% *BCI*	Bootstrapped *p*-value (FDR)	*VIF*
Granular	-0.111	[-0.111, -0.106]	0.625	-0.866	[-0.870, -0.865]	<10^{-4}	1.01
Polar	0.000	[-0.007, 0.002]	0.942	-0.488	[-0.504, -0.495]	0.032	1.04
Parietal	-0.321	[-0.318, -0.315]	0.001	-0.690	[-0.694, -0.691]	<10^{-4}	1.01
Frontal	-0.168	[-0.169, -0.166]	0.035	-0.405	[-0.405, -0.401]	<10^{-4}	1.08
Agranular	0.015	[0.017, 0.021]	0.942	-0.698	[-0.703, -0.698]	<10^{-4}	1.33

Supplemental Table 9: Atlas-based multiple linear regression analyses – Results corresponding to the atlas-based (HCP multi-modal cortical parcellation) analyses discussed in section: 'Biological Correlates of Structure-Function Coupling: Regional perspective' of our **Supplementary Analysis 1** section above. β_{stand}: standardized β coefficient; 95% *BCI*: 95% bootstrapped standardized β coefficient confidence interval; Bootstrapped *p*-value (FDR): bootstrapped *p*-value adjusted for multiple comparisons (two-tailed test; false discovery rate: Benjamini-Hochberg method); *VIF*: Variance Inflation Factor.

Supplemental Table 10

Multiple Comparisons

Dependent Variable: Structure-Function Coupling

Test: Tamhane

(I) Resting-state functional networks	(J) Resting-state functional networks	Mean Difference (I-J)	Std. Error	Sig.	95% Confidence Interval Lower Bound	95% Confidence Interval Upper Bound
Visual	Somatomotor	.011576259845075	.011012956877614	.999	-.022444447855747	.045596967545897
	Dorsal Attention	.078762503502432*	.013690348546591	<.001	.035968531348661	.121556475656204
	Ventral Attention	.118368833753839*	.012161435860379	<.001	.080508179813655	.156229487694023
	Limbic	.128119853216785*	.017555886632451	<.001	.071077052959085	.185162653474485
	Fronto-parietal	.091376027389999*	.012321795324671	<.001	.053080595024469	.129671459755530
	Default Mode	.065777050011911*	.009769426719259	<.001	.035544696323115	.096009403700707

Somatomotor	Visual	-.011576259845075	.011012956877614	.999	-.045596967545897	.022444447855747
	Dorsal Attention	.067186243657357*	.013738242238796	<.001	.024294299424314	.110078187890401
	Ventral Attention	.106792573908764*	.012215325565485	<.001	.068828955779320	.144756192038208
	Limbic	.116543593371710*	.017593260412332	<.001	.059430877784780	.173656308958640
	Fronto-parietal	.079799767544924*	.012374986727580	<.001	.041400518785368	.118199016304480
	Default Mode	.054200790166836*	.009836429987940	<.001	.023866709994600	.084534870339071
Dorsal Attention	Visual	-.078762503502432*	.013690348546591	<.001	-.121556475656204	-.035968531348661
	Somatomotor	-.067186243657357*	.013738242238796	<.001	-.110078187890401	-.024294299424314
	Ventral Attention	.039606330251407	.014674965172352	.161	-.006196449615785	.085409110118598
	Limbic	.049357349714353	.019382033847744	.256	-.012524703142798	.111239402571503
	Fronto-parietal	.012613523887567	.014808130221157	1.000	-.033549539962456	.058776587737590
	Default Mode	-.012985453490521	.012763063074615	1.000	-.053105658048820	.027134751067777
Ventral Attention	Visual	-.118368833753839*	.012161435860379	<.001	-.156229487694023	-.080508179813655
	Somatomotor	-.106792573908764*	.012215325565485	<.001	-.144756192038208	-.068828955779320
	Dorsal Attention	-.039606330251407	.014674965172352	.161	-.085409110118598	.006196449615785
	Limbic	.009751019462946	.018334069786440	1.000	-.049298806097523	.068800845023415
	Fronto-parietal	-.026992806363840	.013407296502490	.635	-.068720703328100	.014735090600420
	Default Mode	-.052591783741928*	.011107234485036	<.001	-.087309135730714	-.017874431753142
Limbic	Visual	-.128119853216785*	.017555886632451	<.001	-.185162653474485	-.071077052959085
	Somatomotor	-.116543593371710*	.017593260412332	<.001	-.173656308958640	-.059430877784780
	Dorsal Attention	-.049357349714353	.019382033847744	.256	-.111239402571503	.012524703142798
	Ventral Attention	-.009751019462946	.018334069786440	1.000	-.068800845023415	.049298806097523
	Fronto-parietal	-.036743825826786	.018440830587884	.678	-.096049043524106	.022561391870535
	Default Mode	-.062342803204874*	.016842781574637	.016	-.117697126604428	-.006988479805320
Fronto-parietal	Visual	-.091376027389999*	.012321795324671	<.001	-.129671459755530	-.053080595024469
	Somatomotor	-.079799767544924*	.012374986727580	<.001	-.118199016304480	-.041400518785368
	Dorsal Attention	-.012613523887567	.014808130221157	1.000	-.058776587737590	.033549539962456
	Ventral Attention	.026992806363840	.013407296502490	.635	-.014735090600420	.068720703328100
	Limbic	.036743825826786	.018440830587884	.678	-.022561391870535	.096049043524106
	Default Mode	-.025598977378088	.011282587280520	.421	-.060782735449272	.009584780693095
Default Mode	Visual	-.065777050011911*	.009769426719259	<.001	-.096009403700707	-.035544696323115
	Somatomotor	-.054200790166836*	.009836429987940	<.001	-.084534870339071	-.023866709994600
	Dorsal Attention	.012985453490521	.012763063074615	1.000	-.027134751067777	.053105658048820
	Ventral Attention	.052591783741928*	.011107234485036	<.001	.017874431753142	.087309135730714
	Limbic	.062342803204874*	.016842781574637	.016	.006988479805320	.117697126604428
	Fronto-parietal	.025598977378088	.011282587280520	.421	-.009584780693095	.060782735449272

*. The mean difference is significant at the 0.05 level.

Supplemental Table 10: Mean differences in structure-function coupling across the 7 resting-state functional networks. One-way ANOVA table with post-hoc correction for multiple comparisons (Tamhane's T2 – equal variances not assumed): Dependent variable: Structure-function coupling; factor: Resting-state functional networks. Data derived from the 100 unrelated HCP subjects, analyzed using the Schaefer 400 atlas.

Supplemental Table 11

Multiple Comparisons

Dependent Variable: Structure-Function Coupling

Test: Tamhane

(I) Cyto-architectonic classes	(J) Cyto-architectonic classes	Mean Difference (I-J)	Std. Error	Sig.	95% Confidence Interval	
					Lower Bound	Upper Bound
Agranular	Frontal	-.040761010523845*	.009823451359097	<.001	-.068695243443140	-.012826777604551
	Parietal	-.069817062841319*	.011092311905753	<.001	-.101304900166407	-.038329225516231
	Polar	.016041661242572	.020855710069885	.997	-.046843672459354	.078926994944498
	Granular	-.118734629145401*	.014489106713137	<.001	-.161682301873357	-.075786956417445
Frontal	Agranular	.040761010523845*	.009823451359097	<.001	.012826777604551	.068695243443140
	Parietal	-.029056052317473*	.009071485027428	.016	-.054766289625380	-.003345815009567
	Polar	.056802671766417	.019854800419733	.080	-.004021710894420	.117627054427254
	Granular	-.077973618621556*	.013007177654396	<.001	-.117592641564863	-.038354595678248
Parietal	Agranular	.069817062841319*	.011092311905753	<.001	.038329225516231	.101304900166407
	Frontal	.029056052317473*	.009071485027428	.016	.003345815009567	.054766289625380
	Polar	.085858724083890*	.020512247232261	.002	.023705782176360	.148011665991421
	Granular	-.048917566304082*	.013990205764855	.013	-.090683100456517	-.007152032151647
Polar	Agranular	-.016041661242572	.020855710069885	.997	-.078926994944498	.046843672459355
	Frontal	-.056802671766417	.019854800419733	.080	-.117627054427254	.004021710894420
	Parietal	-.085858724083890*	.020512247232261	.002	-.148011665991421	-.023705782176360
	Granular	-.134776290387972*	.022531025641270	<.001	-.201952385565034	-.067600195210911
Granular	Agranular	.118734629145401*	.014489106713137	<.001	.075786956417445	.161682301873357
	Frontal	.077973618621556*	.013007177654396	<.001	.038354595678248	.117592641564863
	Parietal	.048917566304082*	.013990205764855	.013	.007152032151647	.090683100456517
	Polar	.134776290387972*	.022531025641270	<.001	.067600195210911	.201952385565034

*. The mean difference is significant at the 0.05 level.

Supplemental Table 11: Mean differences in structure-function coupling across the 5 von-Economo/Koskinas-inspired cyto-architectonic classes. One-way ANOVA table with post-hoc correction for multiple comparisons (Tamhane's T2 – equal variances not assumed): Dependent variable: Structure-function coupling; factor: Cyto-architectonic classes. Data derived from the 100 unrelated HCP subjects, analyzed using the Schaefer 400 atlas.

Supplemental Table 12

Multiple Comparisons

Dependent Variable: Temporal Structure-Function Coupling Variance

Test: Tamhane

(I) Resting-state functional networks	(J) Resting-state functional networks	Mean Difference (I-J)	Std. Error	Sig.	95% Confidence Interval	
					Lower Bound	Upper Bound
Visual	Somatomotor	-.000417954355607	.000154774488292	.158	-.000899728694584	.000063819983371
	Dorsal Attention	.000041389264154	.000177189191989	1.000	-.000508946651812	.000591725180121
	Ventral Attention	-.000097920589750	.000224385624386	1.000	-.000797604509343	.000601763329843
	Limbic	-.005542890908206*	.001032068903494	<.001	-.009008512290002	-.002077269526411
	Fronto-parietal	-.000411572760877	.000206183589545	.648	-.001051869024890	.000228723503135
	Default Mode	-.000794805647197*	.000192588821376	.001	-.001388905098760	-.000200706195633
Somatomotor	Visual	.000417954355607	.000154774488292	.158	-.000063819983371	.000899728694584
	Dorsal Attention	.000459343619761*	.000137748156739	.026	.000028762127004	.000889925112518
	Ventral Attention	.000320033765856	.000194749719790	.903	-.000295069975991	.000935137507704
	Limbic	-.005124936552600*	.001026033511386	<.001	-.008578990223859	-.001670882881341
	Fronto-parietal	.000006381594729	.000173465320814	1.000	-.000537712075290	.000550475264749
	Default Mode	-.000376851291590	.000157063678316	.314	-.000861799723140	.000108097139960
Dorsal Attention	Visual	-.000041389264154	.000177189191989	1.000	-.000591725180121	.000508946651812
	Somatomotor	-.000459343619761*	.000137748156739	.026	-.000889925112518	-.000028762127004
	Ventral Attention	-.000139309853905	.000212998405839	1.000	-.000806550437732	.000527930729923
	Limbic	-.005584280172361*	.001029653162001	<.001	-.009045251909679	-.002123308435042
	Fronto-parietal	-.000452962025032	.000193729411956	.367	-.001056963321183	.000151039271119
	Default Mode	-.000836194911351*	.000179192261490	<.001	-.001389993849054	-.000282395973648
Ventral Attention	Visual	.000097920589750	.000224385624386	1.000	-.000601763329843	.000797604509343
	Somatomotor	-.000320033765856	.000194749719790	.903	-.000935137507704	.000295069975991
	Dorsal Attention	.000139309853905	.000212998405839	1.000	-.000527930729923	.000806550437732
	Limbic	-.005444970318456*	.001038815928205	<.001	-.008923837663188	-.001966102973724
	Fronto-parietal	-.000313652171127	.000237663593615	.988	-.001054042778591	.000426738436337
	Default Mode	-.000696885057446	.000225970717685	.054	-.001399864524700	.000006094409807
Limbic	Visual	.005542890908206*	.001032068903494	<.001	.002077269526411	.009008512290002
	Somatomotor	.005124936552600*	.001026033511386	<.001	.001670882881341	.008578990223859
	Dorsal Attention	.005584280172361*	.001029653162001	<.001	.002123308435042	.009045251909679
	Ventral Attention	.005444970318456*	.001038815928205	<.001	.001966102973724	.008923837663188
	Fronto-parietal	.005131318147329*	.001035036857730	<.001	.001659909172183	.008602727122475
	Default Mode	.004748085261010*	.001032414683342	.002	.001281810846315	.008214359675705

Fronto-parietal	Visual	.000411572760877	.000206183589545	.648	-.000228723503135	.001051869024890
	Somatomotor	-.000006381594729	.000173465320814	1.000	-.000550475264749	.000537712075290
	Dorsal Attention	.000452962025032	.000193729411956	.367	-.000151039271119	.001056963321183
	Ventral Attention	.000313652171127	.000237663593615	.988	-.000426738436337	.001054042778591
	Limbic	-.005131318147329*	.001035036857730	<.001	-.008602727122475	-.001659909172183
	Default Mode	-.000383232886319	.000207907502072	.771	-.001026981642974	.000260515870336
Default Mode	Visual	.000794805647197*	.000192588821376	.001	.000200706195633	.001388905098760
	Somatomotor	.000376851291590	.000157063678316	.314	-.000108097139960	.000861799723140
	Dorsal Attention	.000836194911351*	.000179192261490	<.001	.000282395973648	.001389993849054
	Ventral Attention	.000696885057446	.000225970717685	.054	-.000006094409807	.001399864524700
	Limbic	-.004748085261010*	.001032414683342	.002	-.008214359675705	-.001281810846315
	Fronto-parietal	.000383232886319	.000207907502072	.771	-.000260515870336	.001026981642974

*. The mean difference is significant at the 0.05 level.

Supplemental Table 12: Mean differences in temporal structure-function coupling variance across the 7 resting-state functional networks. One-way ANOVA table with post-hoc correction for multiple comparisons (Tamhane's T2 – equal variances not assumed): Dependent variable: Temporal structure-function coupling variance; factor: Resting-state functional networks. Data derived from the 100 unrelated HCP subjects, analyzed using the Schaefer 400 atlas.

Supplemental Table 13

Multiple Comparisons

Dependent Variable: Temporal Structure-Function Coupling Variance

Test: Tamhane

(I) Cyto-architectonic classes	(J) Cyto-architectonic classes	Mean Difference (I-J)	Std. Error	Sig.	95% Confidence Interval	
					Lower Bound	Upper Bound
Agranular	Frontal	-.000144835313691	.000232862287540	1.000	-.000811675014589	.000522004387207
	Parietal	.000319984782868	.000246927548193	.889	-.000384513851014	.001024483416749
	Polar	-.004950262754159*	.001117304602086	.002	-.008395865122671	-.001504660385646
	Granular	-.000355775530766	.000297647369246	.932	-.001217500451292	.000505949389759
Frontal	Agranular	.000144835313691	.000232862287540	1.000	-.000522004387207	.000811675014589
	Parietal	.000464820096559*	.000140493767067	.011	.000066536816101	.000863103377017
	Polar	-.004805427440467*	.001098696891035	.002	-.008217285007333	-.001393569873602
	Granular	-.000210940217075	.000217621784174	.985	-.000877624317248	.000455743883097
Parietal	Agranular	-.000319984782868	.000246927548193	.889	-.001024483416749	.000384513851014
	Frontal	-.000464820096559*	.000140493767067	.011	-.000863103377017	-.000066536816101
	Polar	-.005270247537026*	.001101763689485	<.001	-.008687546127957	-.001852948946095
	Granular	-.000675760313634	.000232610425487	.064	-.001374068126620	.000022547499352

Polar	Agranular	.004950262754159*	.001117304602086	.002	.001504660385646	.008395865122671
	Frontal	.004805427440467*	.001098696891035	.002	.001393569873602	.008217285007333
	Parietal	.005270247537026*	.001101763689485	<.001	.001852948946095	.008687546127957
	Granular	.004594487223392*	.001114227970314	.004	.001154228370499	.008034746076285
Granular	Agranular	.000355775530766	.000297647369246	.932	-.000505949389759	.001217500451292
	Frontal	.000210940217075	.000217621784174	.985	-.000455743883097	.000877624317248
	Parietal	.000675760313634	.000232610425487	.064	-.000022547499352	.001374068126620
	Polar	-.004594487223392*	.001114227970314	.004	-.008034746076285	-.001154228370499

*. The mean difference is significant at the 0.05 level.

Supplemental Table 13: Mean differences in temporal structure-function coupling variance across the 5 von-Economo/Koskinas-inspired cyto-architectonic classes. One-way ANOVA table with post-hoc correction for multiple comparisons (Tamhane's T2 – equal variances not assumed): Dependent variable: Temporal structure-function coupling variance; factor: Cyto-architectonic classes. Data derived from the 100 unrelated HCP subjects, analyzed using the Schaefer 400 atlas.

Supplemental Table 14

**A. Structure-Function Coupling**							
	**Intracortical Myelin**			**Hurst Exponent**			
**Cortical Type**	β_{stand}	95% *BCI*	Bootstrapped *p*-value (FDR)	β_{stand}	95% *BCI*	Bootstrapped *p*-value (FDR)	*VIF*
Granular	0.178	[0.131, 0.140]	0.305	0.704	[0.717, 0.723]	$6.7x10^{-4}$	1.01
Polar	0.303	[0.233, 0.244]	0.305	-0.275	[-0.264, -0.253]	0.422	1.04
Parietal	0.416	[0.412, 0.415]	$<10^{-4}$	0.538	[0.533, 0.536]	$<10^{-4}$	1.01
Frontal	0.437	[0.437, 0.439]	$<10^{-4}$	0.275	[0.273, 0.276]	$<10^{-4}$	1.08
Agranular	0.456	[0.456, 0.460]	$<10^{-4}$	0.050	[0.055, 0.061]	0.712	1.32
**B. Temporal Structure-Function Coupling Variance**							
	**Intracortical Myelin**			**Hurst Exponent**			
**Cortical Type**	β_{stand}	95% *BCI*	Bootstrapped *p*-value (FDR)	β_{stand}	95% *BCI*	Bootstrapped *p*-value (FDR)	*VIF*

Granular	0.326	[0.285, 0.294]	0.209	-0.462	[-0.447, -0.440]	0.020	1.01
Polar	-0.171	[-0.177, -0.173]	0.209	-0.759	[-0.752, -0.745]	$<10^{-4}$	1.04
Parietal	-0.002	[-0.001, 0.004]	0.999	-0.433	[-0.428, -0.423]	0.006	1.01
Frontal	0.024	[0.024, 0.027]	0.846	-0.428	[-0.425, -0.422]	$<10^{-4}$	1.08
Agranular	-0.106	[-0.102, -0.099]	0.209	-0.627	[-0.612, -0.605]	$<10^{-4}$	1.32

Supplemental Table 14: Atlas-based multiple linear regression analyses – Results corresponding to the atlas-based analyses discussed in section: 'Biological Correlates of Structure-Function Coupling: Regional perspective' of our **Supplementary Analysis 2A** section above. β_{stand}: standardized β coefficient; 95% *BCI*: 95% bootstrapped standardized β coefficient confidence interval; Bootstrapped *p*-value (FDR): bootstrapped *p*-value adjusted for multiple comparisons (two-tailed test; false discovery rate [FDR]: Benjamini-Hochberg method); *VIF*: Variance Inflation Factor.

Supplemental Table 15

Multiple Comparisons

Dependent Variable: Structure-Function Coupling

Test: Tamhane

(I) Resting-state functional networks	(J) Resting-state functional networks	Mean Difference (I-J)	Std. Error	Sig.	95% Confidence Interval Lower Bound	95% Confidence Interval Upper Bound
Visual	Somatomotor	.006595690357668	.011152932253749	1.000	-.028005313256099	.041196693971435
	Dorsal Attention	.089199981977586*	.013443616348244	<.001	.047206436932407	.131193527022764
	Ventral Attention	.115358404655630*	.010886647680366	.000	.081545531250935	.149171278060325
	Limbic	.173452816714172*	.016575717428391	<.001	.120068621897863	.226837011530481
	Fronto-parietal	.098206698052394*	.013127895630619	<.001	.057252070748308	.139161325356481
	Default Mode	.083421262434394*	.010651487279391	<.001	.050477657593982	.116364867274805
Somatomotor	Visual	-.006595690357668	.011152932253749	1.000	-.041196693971435	.028005313256099
	Dorsal Attention	.082604291619917*	.013192885667825	<.001	.041321656829333	.123886926410502
	Ventral Attention	.108762714297962*	.010575467339176	.000	.075867006617745	.141658421978178
	Limbic	.166857126356504*	.016373021101538	<.001	.113974314455770	.219739938257238
	Fronto-parietal	.091611007694726*	.012871016098738	<.001	.051389358434193	.131832656955260
	Default Mode	.076825572076725*	.010333227617186	<.001	.044835858878752	.108815285274699
Dorsal Attention	Visual	-.089199981977585*	.013443616348244	<.001	-.131193527022764	-.047206436932407
	Somatomotor	-.082604291619917*	.013192885667825	<.001	-.123886926410502	-.041321656829333
	Ventral Attention	.026158422678044	.012968555513396	.638	-.014489849006625	.066806694362713

	Limbic	.084252834736587*	.018011627984540	<.001	.026912628460240	.141593041012933
	Fronto-parietal	.009006716074809	.014899865035497	1.000	-.037512243136162	.055525675285780
	Default Mode	-.005778719543192	.012771785922518	1.000	-.045770053655846	.034212614569463
Ventral Attention	Visual	-.115358404655630*	.010886647680366	.000	-.149171278060325	-.081545531250935
	Somatomotor	-.108762714297962*	.010575467339176	.000	-.141658421978178	-.075867006617745
	Dorsal Attention	-.026158422678044	.012968555513396	.638	-.066806694362713	.014489849006625
	Limbic	.058094412058542*	.016192807658066	.019	.005652832643577	.110535991473508
	Fronto-parietal	-.017151706603235	.012640975250102	.984	-.056718410714937	.022414997508466
	Default Mode	-.031937142221236*	.010045237321651	.039	-.063060640844704	-.000813643597768
Limbic	Visual	-.173452816714172*	.016575717428391	<.001	-.226837011530481	-.120068621897863
	Somatomotor	-.166857126356504*	.016373021101538	<.001	-.219739938257238	-.113974314455770
	Dorsal Attention	-.084252834736587*	.018011627984540	<.001	-.141593041012933	-.026912628460240
	Ventral Attention	-.058094412058542*	.016192807658066	.019	-.110535991473508	-.005652832643577
	Fronto-parietal	-.075246118661778*	.017777220418927	.002	-.131918808260778	-.018573429062778
	Default Mode	-.090031554279778*	.016035651012595	<.001	-.142037313087382	-.038025795472175
Fronto-parietal	Visual	-.098206698052394*	.013127895630619	<.001	-.139161325356481	-.057252070748308
	Somatomotor	-.091611007694726*	.012871016098738	<.001	-.131832656955260	-.051389358434193
	Dorsal Attention	-.009006716074809	.014899865035497	1.000	-.055525675285780	.037512243136162
	Ventral Attention	.017151706603235	.012640975250102	.984	-.022414997508466	.056718410714937
	Limbic	.075246118661778*	.017777220418927	.002	.018573429062778	.131918808260778
	Default Mode	-.014785435618001	.012439024833977	.997	-.053668368054729	.024097496818728
Default Mode	Visual	-.083421262434394*	.010651487279391	<.001	-.116364867274805	-.050477657593982
	Somatomotor	-.076825572076725*	.010333227617186	<.001	-.108815285274699	-.044835858878752
	Dorsal Attention	.005778719543192	.012771785922518	1.000	-.034212614569463	.045770053655846
	Ventral Attention	.031937142221236*	.010045237321651	.039	.000813643597768	.063060640844704
	Limbic	.090031554279778*	.016035651012595	<.001	.038025795472175	.142037313087382
	Fronto-parietal	.014785435618001	.012439024833977	.997	-.024097496818728	.053668368054729

*. The mean difference is significant at the 0.05 level.

Supplemental Table 15: Mean differences in structure-function coupling across the 7 resting-state functional networks. One-way ANOVA table with post-hoc correction for multiple comparisons (Tamhane's T2 – equal variances not assumed): Dependent variable: Structure-function coupling; factor: Resting-state functional networks. Data derived from the 100 unrelated HCP subjects, analyzed using the HCP multi-modal atlas.

Supplemental Table 16

Multiple Comparisons

Dependent Variable: Structure-Function Coupling

Test: Tamhane

(I) Cyto-architectonic classes	(J) Cyto-architectonic classes	Mean Difference (I-J)	Std. Error	Sig.	95% Confidence Interval Lower Bound	Upper Bound
Agranular	Frontal	-.031393611993111*	.010588691139072	.036	-.061616598590347	-.001170625395876
	Parietal	-.078536163634367*	.012220454391461	<.001	-.113281772319706	-.043790554949027
	Polar	.042970489686404	.019237787376667	.277	-.014459914419736	.100400893792543
	Granular	-.091632473294457*	.019347038147953	<.001	-.150250601727033	-.033014344861882
Frontal	Agranular	.031393611993111*	.010588691139072	.036	.001170625395876	.061616598590347
	Parietal	-.047142551641255*	.009870464212241	<.001	-.075148010242777	-.019137093039734
	Polar	.074364101679515*	.017837573304158	.003	.019973494345965	.128754709013065
	Granular	-.060238861301346*	.017955345814818	.028	-.116097650215567	-.004380072387125
Parietal	Agranular	.078536163634367*	.012220454391461	<.001	.043790554949028	.113281772319706
	Frontal	.047142551641256*	.009870464212241	<.001	.019137093039734	.075148010242777
	Polar	.121506653320771*	.018852006441658	<.001	.064967695753079	.178045610888462
	Granular	-.013096309660090	.018963479871185	.999	-.070890528238412	.044697908918232
Polar	Agranular	-.042970489686404	.019237787376667	.277	-.100400893792543	.014459914419736
	Frontal	-.074364101679515*	.017837573304158	.003	-.128754709013065	-.019973494345965
	Parietal	-.121506653320771*	.018852006441658	<.001	-.178045610888462	-.064967695753079
	Granular	-.134602962980861*	.024090797546800	<.001	-.206020639278371	-.063185286683350
Granular	Agranular	.091632473294457*	.019347038147953	<.001	.033014344861882	.150250601727033
	Frontal	.060238861301346*	.017955345814818	.028	.004380072387125	.116097650215567
	Parietal	.013096309660090	.018963479871185	.999	-.044697908918232	.070890528238412
	Polar	.134602962980861*	.024090797546800	<.001	.063185286683350	.206020639278371

*. The mean difference is significant at the 0.05 level.

Supplemental Table 16: Mean differences in structure-function coupling across the 5 von-Economo/Koskinas-inspired cyto-architectonic classes. One-way ANOVA table with post-hoc correction for multiple comparisons (Tamhane's T2 – equal variances not assumed): Dependent variable: Structure-function coupling; factor: Cyto-architectonic classes. Data derived from the 100 unrelated HCP subjects, analyzed using the HCP multi-modal atlas.

Supplemental Table 17

Multiple Comparisons

Dependent Variable: Temporal Structure-Function Coupling Variance

Test: Tamhane

Resting-state functional networks	Resting-state functional networks	Mean Difference (I-J)	Std. Error	Sig.	95% Confidence Interval Lower Bound	Upper Bound
Visual	Somatomotor	-.000644763768222*	.000196551583272	.029	-.001255195532850	-.000034332003595
	Dorsal Attention	.000035061888054	.000191377616998	1.000	-.000560977943323	.000631101719430
	Ventral Attention	-.000504000165758	.000231117768225	.495	-.001226258148620	.000218257817105

	Limbic	-.004866947966887*	.000698430782721	<.001	-.007194908386460	-.002538987547314
	Fronto-parietal	-.000704377184006*	.000224930645473	.049	-.001407950665982	-.000000803702030
	Default Mode	-.001498539856996*	.000253648956782	<.001	-.002282887795359	-.000714191918634
Somatomotor	Visual	.000644763768222*	.000196551583272	.029	.000034332003595	.001255195532850
	Dorsal Attention	.000679825656276*	.000206632886918	.029	.000036327730329	.001323323582223
	Ventral Attention	.000140763602465	.000243899939437	1.000	-.000619694384840	.000901221589770
	Limbic	-.004222184198665*	.000702764054232	<.001	-.006559083122257	-.001885285275072
	Fronto-parietal	-.000059613415783	.000238045274982	1.000	-.000802435621620	.000683208790053
	Default Mode	-.000853776088774*	.000265347980857	.034	-.001673715965942	-.000033836211606
Dorsal Attention	Visual	-.000035061888054	.000191377616998	1.000	-.000631101719430	.000560977943323
	Somatomotor	-.000679825656276*	.000206632886918	.029	-.001323323582223	-.000036327730329
	Ventral Attention	-.000539062053811	.000239749969466	.439	-.001288118038365	.000209993930743
	Limbic	-.004902009854941*	.000701334573026	<.001	-.007235991093424	-.002568028616457
	Fronto-parietal	-.000739439072059*	.000233791403483	.045	-.001470613577557	-.000008264566562
	Default Mode	-.001533601745050*	.000261538559961	<.001	-.002342719593896	-.000724483896204
Ventral Attention	Visual	.000504000165758	.000231117768225	.495	-.000218257817105	.001226258148620
	Somatomotor	-.000140763602465	.000243899939437	1.000	-.000901221589770	.000619694384840
	Dorsal Attention	.000539062053811	.000239749969466	.439	-.000209993930743	.001288118038365
	Limbic	-.004362947801129*	.000713204188030	<.001	-.006722053205082	-.002003842397176
	Fronto-parietal	-.000200377018248	.000267298430305	1.000	-.001033752580219	.000632998543722
	Default Mode	-.000994539691239*	.000291877455189	.018	-.001897217734338	-.000091861648140
Limbic	Visual	.004866947966887*	.000698430782721	<.001	.002538987547314	.007194908386460
	Somatomotor	.004222184198665*	.000702764054232	<.001	.001885285275072	.006559083122257
	Dorsal Attention	.004902009854941*	.000701334573026	<.001	.002568028616457	.007235991093424
	Ventral Attention	.004362947801129*	.000713204188030	<.001	.002003842397176	.006722053205082
	Fronto-parietal	.004162570782881*	.000711223302702	<.001	.001807698415285	.006517443150477
	Default Mode	.003368408109890*	.000720820771281	.001	.000993092952972	.005743723266809
Fronto-parietal	Visual	.000704377184006*	.000224930645473	.049	.000000803702030	.001407950665982
	Somatomotor	.000059613415783	.000238045274982	1.000	-.000683208790053	.000802435621620
	Dorsal Attention	.000739439072059*	.000233791403483	.045	.000008264566562	.001470613577557
	Ventral Attention	.000200377018248	.000267298430305	1.000	-.000632998543722	.001033752580219
	Limbic	-.004162570782881*	.000711223302702	<.001	-.006517443150477	-.001807698415285
	Default Mode	-.000794162672991	.000287003173033	.128	-.001682153433412	.000093828087431
Default Mode	Visual	.001498539856996*	.000253648956782	<.001	.000714191918634	.002282887795359
	Somatomotor	.000853776088774*	.000265347980857	.034	.000033836211606	.001673715965942
	Dorsal Attention	.001533601745050*	.000261538559961	<.001	.000724483896204	.002342719593896
	Ventral Attention	.000994539691239*	.000291877455189	.018	.000091861648140	.001897217734338
	Limbic	-.003368408109890*	.000720820771281	.001	-.005743723266809	-.000993092952972

	Fronto-parietal	.000794162672991	.000287003173033	.128	-.000093828087431	.001682153433412

*. The mean difference is significant at the 0.05 level.

Supplemental Table 17: Mean differences in temporal structure-function coupling variance across the 7 resting-state functional networks. One-way ANOVA table with post-hoc correction for multiple comparisons (Tamhane's T2 – equal variances not assumed): Dependent variable: Temporal structure-function coupling variance; factor: Resting-state functional networks. Data derived from the 100 unrelated HCP subjects, analyzed using the HCP multi-modal atlas.

Supplemental Table 18

Multiple Comparisons

Dependent Variable: Temporal Structure-Function Coupling Variance

Test: Tamhane

(I) Cyto-architectonic classes	(J) Cyto-architectonic classes	Mean Difference (I-J)	Std. Error	Sig.	95% Confidence Interval Lower Bound	95% Confidence Interval Upper Bound
Agranular	Frontal	.000029007357304	.000251970747020	1.000	-.000691082944608	.000749097659216
	Parietal	.000466081754380	.000254762283098	.516	-.000262004358687	.001194167867446
	Polar	-.004784469640829*	.000650135247800	<.001	-.006760127105449	-.002808812176209
	Granular	-.000534238374398	.000472671555764	.956	-.001967237622433	.000898760873638
Frontal	Agranular	-.000029007357304	.000251970747020	1.000	-.000749097659216	.000691082944608
	Parietal	.000437074397075	.000178737328713	.142	-.000068353307819	.000942502101970
	Polar	-.004813476998133*	.000624274821067	<.001	-.006738227390162	-.002888726606104
	Granular	-.000563245731702	.000436419077739	.906	-.001925284191606	.000798792728202
Parietal	Agranular	-.000466081754380	.000254762283098	.516	-.001194167867446	.000262004358687
	Frontal	-.000437074397075	.000178737328713	.142	-.000942502101970	.000068353307819
	Polar	-.005250551395209*	.000625406760240	<.001	-.007177448746103	-.003323654044314
	Granular	-.001000320128777	.000438036727855	.282	-.002365286835226	.000364646577671
Polar	Agranular	.004784469640829*	.000650135247800	<.001	.002808812176209	.006760127105449
	Frontal	.004813476998133*	.000624274821067	<.001	.002888726606104	.006738227390162
	Parietal	.005250551395209*	.000625406760240	<.001	.003323654044314	.007177448746103
	Granular	.004250231266431*	.000741382623544	<.001	.002044982752900	.006455479779963
Granular	Agranular	.000534238374398	.000472671555764	.956	-.000898760873638	.001967237622433
	Frontal	.000563245731702	.000436419077739	.906	-.000798792728202	.001925284191606
	Parietal	.001000320128777	.000438036727855	.282	-.000364646577671	.002365286835226
	Polar	-.004250231266431*	.000741382623544	<.001	-.006455479779963	-.002044982752900

*. The mean difference is significant at the 0.05 level.

Supplemental Table 18: Mean differences in temporal structure-function coupling variance across the 5 von-Economo/Koskinas-inspired cyto-architectonic classes. One-way ANOVA table with post-hoc correction for

multiple comparisons (Tamhane's T2 – equal variances not assumed): Dependent variable: Temporal structure-function coupling variance; factor: Cyto-architectonic classes. Data derived from the 100 unrelated HCP subjects, analyzed using the HCP multi-modal atlas.

Supplemental Table 19

**A. Structure-Function Coupling**							
	**Intracortical Myelin**			**Hurst Exponent**			
**Cortical Type**	β_{stand}	95% *BCI*	Bootstrapped *p*-value (FDR)	β_{stand}	95% *BCI*	Bootstrapped *p*-value (FDR)	*VIF*
Granular	0.384	[0.379, 0.387]	0.055	0.451	[0.463, 0.472]	0.040	1.01
Polar	-0.387	[-0.394, -0.388]	0.030	0.128	[0.105, 0.112]	0.614	1.04
Parietal	0.424	[0.430, 0.435]	$<10^{-4}$	0.342	[0.334, 0.338]	0.016	1.01
Frontal	0.420	[0.419, 0.422]	$<10^{-4}$	0.227	[0.222, 0.225]	0.040	1.08
Agranular	0.488	[0.477, 0.482]	0.005	-0.026	[-0.038, -0.033]	0.800	1.32
**B. Temporal Structure-Function Coupling Variance**							
	**Intracortical Myelin**			**Hurst Exponent**			
**Cortical Type**	β_{stand}	95% *BCI*	Bootstrapped *p*-value (FDR)	β_{stand}	95% *BCI*	Bootstrapped *p*-value (FDR)	*VIF*
Granular	-0.183	[-0.183, -0.176]	0.773	-0.741	[-0.746, -0.739]	$<10^{-4}$	1.01
Polar	0.018	[0.005, 0.009]	0.867	-0.847	[-0.859, -0.854]	$<10^{-4}$	1.04
Parietal	-0.162	[-0.159, -0.157]	0.063	-0.757	[-0.760, -0.756]	$<10^{-4}$	1.01
Frontal	-0.041	[-0.042, -0.040]	0.819	-0.540	[-0.540, -0.536]	$<10^{-4}$	1.08
Agranular	0.046	[0.038, 0.042]	0.819	-0.756	[-0.757, -0.752]	$<10^{-4}$	1.32

Supplemental Table 19: Atlas-based multiple linear regression analyses – Results corresponding to the atlas-based (HCP multi-modal cortical parcellation) analyses discussed in section: 'Biological Correlates of Structure-Function Coupling: Regional perspective' of our **Supplementary Analysis 2B** section above. β_{stand}: standardized β coefficient; 95% *BCI*: 95% bootstrapped standardized β coefficient confidence interval; Bootstrapped *p*-value (FDR):

bootstrapped *p*-value adjusted for multiple comparisons (two-tailed test; false discovery rate: Benjamini-Hochberg method); *VIF*: Variance Inflation Factor.

Supplemental Table 20

Multiple Comparisons

Dependent Variable: Structure-Function Coupling

Test: Tamhane

(I) Resting-state functional networks	(J) Resting-state functional networks	Mean Difference (I-J)	Std. Error	Sig.	95% Confidence Interval Lower Bound	95% Confidence Interval Upper Bound
Visual	Somatomotor	.046571860093*	.012085556725	.004	.00924424789	.08389947230
	Dorsal Attention	.102452385791*	.013670633892	<.001	.05973252997	.14517224161
	Ventral Attention	.074065390806*	.020485675619	.013	.00931098240	.13881979921
	Limbic	.072603990842	.024628215535	.125	-.00934961429	.15455759597
	Fronto-parietal	.116884042448*	.023993919714	<.001	.04098685964	.19278122526
	Default Mode	.095750893648*	.019238360260	<.001	.03613178235	.15537000494
Somatomotor	Visual	-.046571860093*	.012085556725	.004	-.08389947230	-.00924424789
	Dorsal Attention	.055880525698*	.014531189922	.004	.01068604928	.10107500212
	Ventral Attention	.027493530713	.021069697556	.990	-.03883480452	.09382186595
	Limbic	.026032130749	.025116095437	1.000	-.05695669605	.10902095755
	Fronto-parietal	.070312182356	.024494436773	.110	-.00692075104	.14754511575
	Default Mode	.049179033555	.019859097538	.265	-.01223354095	.11059160806
Dorsal Attention	Visual	-.102452385791*	.013670633892	<.001	-.14517224161	-.05973252997
	Somatomotor	-.055880525698*	.014531189922	.004	-.10107500212	-.01068604928
	Ventral Attention	-.028386994985	.022017213829	.991	-.09746692193	.04069293196
	Limbic	-.029848394949	.025916091519	.998	-.11471777540	.05502098550
	Fronto-parietal	.014431656657	.025314086641	1.000	-.06512206101	.09398537432
	Default Mode	-.006701492143	.020861670707	1.000	-.07120701796	.05780403367
Ventral Attention	Visual	-.074065390806*	.020485675619	.013	-.13881979921	-.00931098240
	Somatomotor	-.027493530713	.021069697556	.990	-.09382186595	.03883480452
	Dorsal Attention	.028386994985	.022017213829	.991	-.04069293196	.09746692193
	Limbic	-.001461399965	.030073584326	1.000	-.09728164658	.09435884665
	Fronto-parietal	.042818651642	.029556380986	.968	-.04927491838	.13491222166
	Default Mode	.021685502842	.025845424726	1.000	-.05843550184	.10180650752
Limbic	Visual	-.072603990842	.024628215535	.125	-.15455759597	.00934961429
	Somatomotor	-.026032130749	.025116095437	1.000	-.10902095755	.05695669605
	Dorsal Attention	.029848394949	.025916091519	.998	-.05502098550	.11471777540
	Ventral Attention	.001461399965	.030073584326	1.000	-.09435884665	.09728164658

	Fronto-parietal	.044280051607	.032564793134	.984	-.05867814848	.14723825169
	Default Mode	.023146902806	.029238195466	1.000	-.07001315299	.11630695860
Fronto-parietal	Visual	-.116884042448*	.023993919714	<.001	-.19278122526	-.04098685964
	Somatomotor	-.070312182356	.024494436773	.110	-.14754511575	.00692075104
	Dorsal Attention	-.014431656657	.025314086641	1.000	-.09398537432	.06512206101
	Ventral Attention	-.042818651642	.029556380986	.968	-.13491222166	.04927491838
	Limbic	-.044280051607	.032564793134	.984	-.14723825169	.05867814848
	Default Mode	-.021133148800	.028705944627	1.000	-.11031381899	.06804752139
Default Mode	Visual	-.095750893648*	.019238360260	<.001	-.15537000494	-.03613178235
	Somatomotor	-.049179033555	.019859097538	.265	-.11059160806	.01223354095
	Dorsal Attention	.006701492143	.020861670707	1.000	-.05780403367	.07120701796
	Ventral Attention	-.021685502842	.025845424726	1.000	-.10180650752	.05843550184
	Limbic	-.023146902806	.029238195466	1.000	-.11630695860	.07001315299
	Fronto-parietal	.021133148800	.028705944627	1.000	-.06804752139	.11031381899

*. The mean difference is significant at the 0.05 level.

Supplemental Table 20: Mean differences in structure-function coupling across the 7 resting-state functional networks. One-way ANOVA table with post-hoc correction for multiple comparisons (Tamhane's T2 – equal variances not assumed): Dependent variable: Structure-function coupling; factor: Resting-state functional networks. Data derived from the 14 Penn subjects, analyzed using the Schaefer 400 atlas.

Supplemental Table 21

Multiple Comparisons

Dependent Variable: Structure-Function Coupling

Test: Tamhane

(I) Cyto-architectonic classes	(J) Cyto-architectonic classes	Mean Difference (I-J)	Std. Error	Sig.	95% Confidence Interval Lower Bound	Upper Bound
Agranular	Frontal	-.015546811608	.019274271698	.996	-.07041831243	.03932468922
	Parietal	-.063639772380*	.018726047030	.009	-.11707375860	-.01020578616
	Polar	-.025422840145	.030967215421	.995	-.11747328708	.06662760679
	Granular	-.077815060676	.026796084602	.055	-.15662981791	.00099969656
Frontal	Agranular	.015546811608	.019274271698	.996	-.03932468922	.07041831243
	Parietal	-.048092960772*	.013061719163	.003	-.08498008565	-.01120583589
	Polar	-.009876028537	.027908996776	1.000	-.09522582511	.07547376804
	Granular	-.062268249068	.023194262655	.114	-.13272992081	.00819342267
Parietal	Agranular	.063639772380*	.018726047030	.009	.01020578616	.11707375860
	Frontal	.048092960772*	.013061719163	.003	.01120583589	.08498008565
	Polar	.038216932235	.027533241526	.858	-.04641967292	.12285353739

	Granular	-.014175288296	.022740736751	1.000	-.08374971571	.05539913911
Polar	Agranular	.025422840145	.030967215421	.995	-.06662760679	.11747328708
	Frontal	.009876028537	.027908996776	1.000	-.07547376804	.09522582511
	Parietal	-.038216932235	.027533241526	.858	-.12285353739	.04641967292
	Granular	-.052392220531	.033547648227	.742	-.15213637083	.04735192977
Granular	Agranular	.077815060676	.026796084602	.055	-.00099969656	.15662981791
	Frontal	.062268249068	.023194262655	.114	-.00819342267	.13272992081
	Parietal	.014175288296	.022740736751	1.000	-.05539913911	.08374971571
	Polar	.052392220531	.033547648227	.742	-.04735192977	.15213637083

*. The mean difference is significant at the 0.05 level.

Supplemental Table 21: Mean differences in structure-function coupling across the 5 von-Economo/Koskinas-inspired cyto-architectonic classes. One-way ANOVA table with post-hoc correction for multiple comparisons (Tamhane's T2 – equal variances not assumed): Dependent variable: Structure-function coupling; factor: Cyto-architectonic classes. Data derived from the 14 Penn subjects, analyzed using the Schaefer 400 atlas.

Supplemental Table 22

Multiple Comparisons

Dependent Variable: Temporal Structure-Function Coupling Variance

Test: Tamhane

(I) Resting-state functional networks	(J) Resting-state functional networks	Mean Difference (I-J)	Std. Error	Sig.	95% Confidence Interval Lower Bound	95% Confidence Interval Upper Bound
Visual	Somatomotor	-.019355395635*	.004450437805	<.001	-.03323580953	-.00547498174
	Dorsal Attention	-.006240959101	.003365738827	.775	-.01688583165	.00440391345
	Ventral Attention	-.032452431217*	.008241598832	.006	-.05881187431	-.00609298812
	Limbic	-.044655912932*	.008553793779	<.001	-.07360632101	-.01570550485
	Fronto-parietal	-.044847718017*	.011568246138	.006	-.08177013595	-.00792530009
	Default Mode	-.039087689790*	.007146225690	<.001	-.06136953406	-.01680584552
Somatomotor	Visual	.019355395635*	.004450437805	<.001	.00547498174	.03323580953
	Dorsal Attention	.013114436534	.005267744251	.259	-.00319419845	.02942307152
	Ventral Attention	-.013097035582	.009183947025	.973	-.04195761841	.01576354725
	Limbic	-.025300517297	.009465110652	.212	-.05619063183	.00558959723
	Fronto-parietal	-.025492322382	.012257456929	.591	-.06421075753	.01322611277
	Default Mode	-.019732294155	.008215197701	.312	-.04509762238	.00563303407
Dorsal Attention	Visual	.006240959101	.003365738827	.775	-.00440391345	.01688583165
	Somatomotor	-.013114436534	.005267744251	.259	-.02942307152	.00319419845
	Ventral Attention	-.026211472116	.008710148342	.078	-.05379386338	.00137091915
	Limbic	-.038414953832*	.009006115747	.004	-.06827923828	-.00855066938

	Fronto-parietal	-.038606758917*	.011906596976	.041	-.07640153922	-.00081197861
	Default Mode	-.032846730689*	.007681879617	<.001	-.05666289517	-.00903056621
Ventral Attention	Visual	.032452431217*	.008241598832	.006	.00609298812	.05881187431
	Somatomotor	.013097035582	.009183947025	.973	-.01576354725	.04195761841
	Dorsal Attention	.026211472116	.008710148342	.078	-.00137091915	.05379386338
	Limbic	-.012203481715	.011734814626	.999	-.04934599515	.02493903172
	Fronto-parietal	-.012395286800	.014084133094	1.000	-.05636245651	.03157188291
	Default Mode	-.006635258573	.010752070866	1.000	-.04000910419	.02673858704
Limbic	Visual	.044655912932*	.008553793779	<.001	.01570550485	.07360632101
	Somatomotor	.025300517297	.009465110652	.212	-.00558959723	.05619063183
	Dorsal Attention	.038414953832*	.009006115747	.004	.00855066938	.06827923828
	Ventral Attention	.012203481715	.011734814626	.999	-.02493903172	.04934599515
	Fronto-parietal	-.000191805085	.014269065902	1.000	-.04502549228	.04464188211
	Default Mode	.005568223142	.010993200836	1.000	-.02926650112	.04040294740
Fronto-parietal	Visual	.044847718017*	.011568246138	.006	.00792530009	.08177013595
	Somatomotor	.025492322382	.012257456929	.591	-.01322611277	.06421075753
	Dorsal Attention	.038606758917*	.011906596976	.041	.00081197861	.07640153922
	Ventral Attention	.012395286800	.014084133094	1.000	-.03157188291	.05636245651
	Limbic	.000191805085	.014269065902	1.000	-.04464188211	.04502549228
	Default Mode	.005760028227	.013472468048	1.000	-.03632039553	.04784045198
Default Mode	Visual	.039087689790*	.007146225690	<.001	.01680584552	.06136953406
	Somatomotor	.019732294155	.008215197701	.312	-.00563303407	.04509762238
	Dorsal Attention	.032846730689*	.007681879617	<.001	.00903056621	.05666289517
	Ventral Attention	.006635258573	.010752070866	1.000	-.02673858704	.04000910419
	Limbic	-.005568223142	.010993200836	1.000	-.04040294740	.02926650112
	Fronto-parietal	-.005760028227	.013472468048	1.000	-.04784045198	.03632039553

*. The mean difference is significant at the 0.05 level.

Supplemental Table 22: Mean differences in temporal structure-function coupling variance across the 7 resting-state functional networks. One-way ANOVA table with post-hoc correction for multiple comparisons (Tamhane's T2 – equal variances not assumed): Dependent variable: Temporal structure-function coupling variance; factor: Resting-state functional networks. Data derived from the 14 Penn subjects, analyzed using the Schaefer 400 atlas.

Supplemental Table 23

Multiple Comparisons

Dependent Variable: Temporal Structure-Function Coupling Variance

Test: Tamhane

(I) Cyto-architectonic classes	(J) Cyto-architectonic classes	Mean Difference (I-J)	Std. Error	Sig.	95% Confidence Interval Lower Bound	Upper Bound
Agranular	Frontal	.009639618584	.007829158548	.917	-.01259897913	.03187821630
	Parietal	.024060176447*	.007240128943	.012	.00339749917	.04472285372
	Polar	.012948393733	.011367563118	.952	-.02068152742	.04657831488
	Granular	.020581815956	.012723846972	.706	-.01755193020	.05871556211
Frontal	Agranular	-.009639618584	.007829158548	.917	-.03187821630	.01259897913
	Parietal	.014420557863	.005549221440	.094	-.00124381476	.03008493049
	Polar	.003308775148	.010372843521	1.000	-.02803310449	.03465065478
	Granular	.010942197372	.011843592076	.989	-.02530821289	.04719260763
Parietal	Agranular	-.024060176447*	.007240128943	.012	-.04472285372	-.00339749917
	Frontal	-.014420557863	.005549221440	.094	-.03008493049	.00124381476
	Polar	-.011111782715	.009935775070	.959	-.04157680686	.01935324143
	Granular	-.003478360491	.011462740370	1.000	-.03901723240	.03206051142
Polar	Agranular	-.012948393733	.011367563118	.952	-.04657831488	.02068152742
	Frontal	-.003308775148	.010372843521	1.000	-.03465065478	.02803310449
	Parietal	.011111782715	.009935775070	.959	-.01935324143	.04157680686
	Granular	.007633422224	.014429013858	1.000	-.03526148801	.05052833246
Granular	Agranular	-.020581815956	.012723846972	.706	-.05871556211	.01755193020
	Frontal	-.010942197372	.011843592076	.989	-.04719260763	.02530821289
	Parietal	.003478360491	.011462740370	1.000	-.03206051142	.03901723240
	Polar	-.007633422224	.014429013858	1.000	-.05052833246	.03526148801

*. The mean difference is significant at the 0.05 level.

Supplemental Table 23: Mean differences in temporal structure-function coupling variance across the 5 von-Economo/Koskinas-inspired cyto-architectonic classes. One-way ANOVA table with post-hoc correction for multiple comparisons (Tamhane's T2 – equal variances not assumed): Dependent variable: Temporal structure-function coupling; factor: Cyto-architectonic classes. Data derived from the 14 Penn subjects, analyzed using the Schaefer 400 atlas.

Supplemental Table 24

**A. Structure-Function Coupling**									
	**Intracortical Myelin**				**Hurst Exponent**				
**Cortical Type**	β_{stand}	95% *BCI*	Bootstrapped *p*-value	Bootstrapped *p*-value (FDR)	β_{stand}	95% *BCI*	Bootstrapped *p*-value	Bootstrapped *p*-value (FDR)	*VIF*
Granular	0.128	[0.101, 0.112]	0.653	0.653	0.498	[0.483, 0.494]	0.091	0.152	1.37
Polar	0.536	[0.483, 0.497]	0.107	0.134	-0.540	[-0.545, -0.535]	0.016	0.040	1.18
Parietal	0.410	[0.410, 0.415]	0.001	0.005	0.255	[0.251, 0.255]	0.010	0.040	1.11

Cortical Type	β_{stand}	95% *BCI*	Bootstrapped *p*-value	Bootstrapped *p*-value (FDR)	β_{stand}	95% *BCI*	Bootstrapped *p*-value	Bootstrapped *p*-value (FDR)	*VIF*
Frontal	0.144	[0.144, 0.147]	0.102	0.134	0.019	[0.018, 0.021]	0.731	0.731	1.00
Agranular	0.269	[0.265, 0.271]	0.044	0.110	-0.040	[-0.042, -0.038]	0.721	0.731	1.00
**B. Temporal Structure-Function Coupling Variance**									
	**Intracortical Myelin**				**Hurst Exponent**				
**Cortical Type**	β_{stand}	95% *BCI*	Bootstrapped *p*-value	Bootstrapped *p*-value (FDR)	β_{stand}	95% *BCI*	Bootstrapped *p*-value	Bootstrapped *p*-value (FDR)	*VIF*
Granular	-0.873	[-0.876, -0.865]	4x10^{-4}	5x10^{-4}	-0.311	[-0.303, -0.297]	0.030	0.050	1.37
Polar	-0.211	[-0.024, -0.001]	0.651	0.651	0.116	[0.114, 0.129]	0.811	0.855	1.18
Parietal	-0.401	[-0.406, -0.403]	<10^{-4}	<10^{-4}	-0.465	[-0.472, -0.468]	<10^{-4}	<10^{-4}	1.11
Frontal	-0.362	[-0.365, -0.362]	<10^{-4}	<10^{-4}	-0.159	[-0.161, -0.159]	0.003	0.007	1.00
Agranular	-0.394	[-0.394, -0.390]	2x10^{-4}	3.3x10^{-4}	0.022	[0.020, 0.025]	0.855	0.855	1.00

Supplemental Table 24: Atlas-based multiple linear regression analyses – Results corresponding to the atlas-based (Schaefer cortical parcellation) analyses discussed in section: 'Biological Correlates of Structure-Function Coupling: Regional perspective' of our **Supplementary Analysis 3** section above. β_{stand}: standardized β coefficient; 95% *BCI*: 95% bootstrapped standardized β coefficient confidence interval; Bootstrapped *p*-value: bootstrapped *p*-value (two-tailed test); Bootstrapped *p*-value (FDR): bootstrapped *p*-value adjusted for multiple comparisons (two-tailed test; false discovery rate: Benjamini-Hochberg method); *VIF*: Variance Inflation Factor.

Supplemental Table 25

Multiple Comparisons

Dependent Variable: Structure-Function Coupling

Test: Tamhane

(I) Resting-state functional networks	(J) Resting-state functional networks	Mean Difference (I-J)	Std. Error	Sig.	95% Confidence Interval Lower Bound	95% Confidence Interval Upper Bound
Visual	Somatomotor	-0.106235409*	0.001233326	<.001	-0.109973804	-0.102497014
	Dorsal Attention	-0.044014034*	0.001368841	<.001	-0.048163435	-0.039864633
	Ventral Attention	-0.032940312*	0.001563191	<.001	-0.037678993	-0.028201631
	Limbic	0.013946968*	0.001398494	<.001	0.009707501	0.018186436
	Fronto-parietal	-0.015712364*	0.001358959	<.001	-0.019831683	-0.011593045
	Default Mode	-0.020298196*	0.001163993	<.001	-0.023826305	-0.016770087
Somatomotor	Visual	0.106235409*	0.001233326	<.001	0.102497014	0.109973804
	Dorsal Attention	0.062221375*	0.001501823	<.001	0.05766897	0.066773781
	Ventral Attention	0.073295097*	0.001672613	<.001	0.068224886	0.078365308
	Limbic	0.120182377*	0.001524345	<.001	0.115561576	0.124803179
	Fronto-parietal	0.090523045*	0.001490896	<.001	0.08600386	0.09504223
	Default Mode	0.085937213*	0.001310131	<.001	0.081966095	0.089908331

Dorsal Attention	Visual	0.044014034*	0.001368841	<.001	0.039864633	0.048163435
	Somatomotor	-0.062221375*	0.001501823	<.001	-0.066773781	-0.05766897
	Ventral Attention	0.011073722*	0.001781448	<.001	0.005673606	0.016473837
	Limbic	0.057961002*	0.001641708	<.001	0.052984396	0.062937608
	Fronto-parietal	0.02830167*	0.001604262	<.001	0.023438775	0.033164564
	Default Mode	0.023715838*	0.001443879	<.001	0.019339094	0.028092581
Ventral Attention	Visual	0.032940312*	0.001563191	<.001	0.028201631	0.037678993
	Somatomotor	-0.073295097*	0.001672613	<.001	-0.078365308	-0.068224886
	Dorsal Attention	-0.011073722*	0.001781448	<.001	-0.016473837	-0.005673606
	Limbic	0.046887281*	0.001803335	<.001	0.041420738	0.052353823
	Fronto-parietal	0.017227948*	0.001771489	<.001	0.011858075	0.022597822
	Default Mode	0.012642116*	0.001622321	<.001	0.007724333	0.017559899
Limbic	Visual	-0.013946968*	0.001398494	<.001	-0.018186436	-0.009707501
	Somatomotor	-0.120182377*	0.001524345	<.001	-0.124803179	-0.115561576
	Dorsal Attention	-0.057961002*	0.001641708	<.001	-0.062937608	-0.052984396
	Ventral Attention	-0.046887281*	0.001803335	<.001	-0.052353823	-0.041420738
	Fronto-parietal	-0.029659332*	0.001633931	<.001	-0.034612298	-0.024706366
	Default Mode	-0.034245165*	0.001470567	<.001	-0.038702963	-0.029787366
Fronto-parietal	Visual	0.015712364*	0.001358959	<.001	0.011593045	0.019831683
	Somatomotor	-0.090523045*	0.001490896	<.001	-0.09504223	-0.08600386
	Dorsal Attention	-0.02830167*	0.001604262	<.001	-0.033164564	-0.023438775
	Ventral Attention	-0.017227948*	0.001771489	<.001	-0.022597822	-0.011858075
	Limbic	0.029659332*	0.001633931	<.001	0.024706366	0.034612298
	Default Mode	-0.004585832*	0.0014309	<.001	-0.008923119	-0.000248545
Default Mode	Visual	0.020298196*	0.001163993	<.001	0.016770087	0.023826305
	Somatomotor	-0.085937213*	0.001310131	<.001	-0.089908331	-0.081966095
	Dorsal Attention	-0.023715838*	0.001443879	<.001	-0.028092581	-0.019339094
	Ventral Attention	-0.012642116*	0.001622321	<.001	-0.017559899	-0.007724333
	Limbic	0.034245165*	0.001470567	<.001	0.029787366	0.038702963
	Fronto-parietal	0.004585832*	0.0014309	<.001	0.000248545	0.008923119

*. The mean difference is significant at the 0.05 level.

Supplemental Table 25: Mean differences in structure-function coupling across the 7 resting-state functional networks. One-way ANOVA table with post-hoc correction for multiple comparisons (Tamhane's T2 – equal variances not assumed): Dependent variable: Structure-function coupling; factor: Resting-state functional networks. Data reported in each table column were averaged across the 9 subjects scanned at Penn that passed quality-control and analyzed using our voxel-based analysis approach; the *p*-values reported under the 'Sig.' column represent the combined p_{fisher} value across the 9 subjects.

Supplemental Table 26

Multiple Comparisons

Dependent Variable: Structure-Function Coupling

Test: Tamhane

(I) Cyto-architectonic classes	(J) Cyto-architectonic classes	Mean Difference (I-J)	Std. Error	Sig.	95% Confidence Interval	
					Lower Bound	Upper Bound
Agranular	Frontal	-1.10E-03*	1.27E-03	<.001	-4.65E-03	2.45E-03
	Parietal	-1.35E-02*	1.32E-03	<.001	-1.72E-02	-9.81E-03
	Polar	1.59E-02*	1.68E-03	<.001	1.12E-02	2.06E-02
	Granular	-2.74E-02*	1.78E-03	<.001	-3.23E-02	-2.24E-02
Frontal	Agranular	1.10E-03*	1.27E-03	<.001	-2.45E-03	4.65E-03
	Parietal	-1.24E-02*	1.02E-03	<.001	-1.53E-02	-9.55E-03
	Polar	1.70E-02*	1.45E-03	<.001	1.29E-02	2.10E-02
	Granular	-2.63E-02*	1.56E-03	<.001	-3.06E-02	-2.19E-02
Parietal	Agranular	1.35E-02*	1.32E-03	<.001	9.81E-03	1.72E-02
	Frontal	1.24E-02*	1.02E-03	<.001	9.55E-03	1.53E-02
	Polar	2.94E-02*	1.50E-03	<.001	2.52E-02	3.36E-02
	Granular	-1.39E-02*	1.61E-03	<.001	-1.83E-02	-9.35E-03
Polar	Agranular	-1.59E-02*	1.68E-03	<.001	-2.06E-02	-1.12E-02
	Frontal	-1.70E-02*	1.45E-03	<.001	-2.10E-02	-1.29E-02
	Parietal	-2.94E-02*	1.50E-03	<.001	-3.36E-02	-2.52E-02
	Granular	-4.32E-02*	1.91E-03	<.001	-4.86E-02	-3.79E-02
Granular	Agranular	2.74E-02*	1.78E-03	<.001	2.24E-02	3.23E-02
	Frontal	2.63E-02*	1.56E-03	<.001	2.19E-02	3.06E-02
	Parietal	1.39E-02*	1.61E-03	<.001	9.35E-03	1.83E-02
	Polar	4.32E-02*	1.91E-03	<.001	3.79E-02	4.86E-02

*. The mean difference is significant at the 0.05 level.

Supplemental Table 26: Mean differences in structure-function coupling across the 5 von-Economo/Koskinas-inspired cyto-architectonic classes. One-way ANOVA table with post-hoc correction for multiple comparisons (Tamhane's T2 – equal variances not assumed): Dependent variable: Structure-function coupling; factor: Cyto-architectonic classes. Data reported in each table column were averaged across the 9 subjects scanned at Penn that passed quality-control and analyzed using our voxel-based analysis approach; the *p*-values reported under the 'Sig.' column represent the combined p_{fisher} value across the 9 subjects.

Supplemental Table 27

Multiple Comparisons

Dependent Variable: Temporal Structure-Function Coupling Variance

Test: Tamhane

Resting-state functional networks	Resting-state functional networks	Mean Difference (I-J)	Std. Error	Sig.	95% Confidence Interval	
					Lower Bound	Upper Bound
Visual	Somatomotor	-0.000152648*	4.7316E-05	<.001	-0.0002961	-9.225E-06
	Dorsal Attention	0.000929401*	4.3957E-05	<.001	0.00079616	0.00106264
	Ventral Attention	0.000527939*	4.9728E-05	<.001	0.0003772	0.00067868
	Limbic	-0.000866467*	7.4769E-05	<.001	-0.0010931	-0.0006398
	Fronto-parietal	-0.000511196*	5.8235E-05	<.001	-0.0006877	-0.0003347
	Default Mode	-0.002924993*	6.4663E-05	<.001	-0.003121	-0.002729
Somatomotor	Visual	0.000152648*	4.7316E-05	<.001	9.2247E-06	0.00029607
	Dorsal Attention	0.001082049*	4.488E-05	<.001	0.00094601	0.00121809
	Ventral Attention	0.000680587*	5.0361E-05	<.001	0.00052793	0.00083324
	Limbic	-0.000713819*	7.4603E-05	<.001	-0.00094	-0.0004877
	Fronto-parietal	-0.000358549*	5.8634E-05	<.001	-0.0005363	-0.0001808
	Default Mode	-0.002772345*	6.4748E-05	<.001	-0.0029686	-0.0025761
Dorsal Attention	Visual	-0.000929401*	4.3957E-05	<.001	-0.0010626	-0.0007962
	Somatomotor	-0.001082049*	4.488E-05	<.001	-0.0012181	-0.000946
	Ventral Attention	-0.000401462*	4.7281E-05	<.001	-0.0005448	-0.0002581
	Limbic	-0.001795868*	7.2694E-05	<.001	-0.0020163	-0.0015755
	Fronto-parietal	-0.001440597*	5.5968E-05	<.001	-0.0016103	-0.0012709
	Default Mode	-0.003854394*	6.2442E-05	<.001	-0.0040437	-0.0036651
Ventral Attention	Visual	-0.000527939*	4.9728E-05	<.001	-0.0006787	-0.0003772
	Somatomotor	-0.000680587*	5.0361E-05	<.001	-0.0008332	-0.0005279
	Dorsal Attention	0.000401462*	4.7281E-05	<.001	0.00025814	0.00054479
	Limbic	-0.001394406*	7.5947E-05	<.001	-0.0016246	-0.0011642
	Fronto-parietal	-0.001039135*	6.0139E-05	<.001	-0.0012214	-0.0008568
	Default Mode	-0.003452932*	6.605E-05	<.001	-0.0036531	-0.0032527
Limbic	Visual	0.000866467*	7.4769E-05	<.001	0.00063979	0.00109314
	Somatomotor	0.000713819*	7.4603E-05	<.001	0.00048765	0.00093999
	Dorsal Attention	0.001795868*	7.2694E-05	<.001	0.00157548	0.00201625
	Ventral Attention	0.001394406*	7.5947E-05	<.001	0.00116417	0.00162464
	Fronto-parietal	0.000355271*	8.1684E-05	<.001	0.00010765	0.00060289
	Default Mode	-0.002058526*	8.5938E-05	<.001	-0.002319	-0.001798
Fronto-parietal	Visual	0.000511196*	5.8235E-05	<.001	0.00033467	0.00068772
	Somatomotor	0.000358549*	5.8634E-05	<.001	0.00018081	0.00053628
	Dorsal Attention	0.001440597*	5.5968E-05	<.001	0.00127094	0.00161025
	Ventral Attention	0.001039135*	6.0139E-05	<.001	0.00085684	0.00122143
	Limbic	-0.000355271*	8.1684E-05	<.001	-0.0006029	-0.0001077

	Default Mode	-0.002413797*	7.2447E-05	<.001	-0.0026334	-0.0021942
Default Mode	Visual	0.002924993*	6.4663E-05	<.001	0.00272899	0.00312099
	Somatomotor	0.002772345*	6.4748E-05	<.001	0.00257609	0.0029686
	Dorsal Attention	0.003854394*	6.2442E-05	<.001	0.00366513	0.00404366
	Ventral Attention	0.003452932*	6.605E-05	<.001	0.00325273	0.00365314
	Limbic	0.002058526*	8.5938E-05	<.001	0.00179802	0.00231903
	Fronto-parietal	0.002413797*	7.2447E-05	<.001	0.0021942	0.00263339

*. The mean difference is significant at the 0.05 level.

Supplemental Table 27: Mean differences in temporal structure-function coupling variance across the 7 resting-state functional networks. One-way ANOVA table with post-hoc correction for multiple comparisons (Tamhane's T2 – equal variances not assumed): Dependent variable: Temporal structure-function coupling variance; factor: Resting-state functional networks. Data reported in each table column were averaged across the 9 subjects scanned at Penn that passed quality-control and analyzed using our voxel-based analysis approach; the *p*-values reported under the 'Sig.' column represent the combined p_{fisher} value across the 9 subjects.

Supplemental Table 28

Multiple Comparisons

Dependent Variable: Temporal Structure-Function Coupling Variance

Test: Tamhane

(I) Cyto-architectonic classes	(J) Cyto-architectonic classes	Mean Difference (I-J)	Std. Error	Sig.	95% Confidence Interval Lower Bound	95% Confidence Interval Upper Bound
Agranular	Frontal	-0.001311816*	5.1232E-05	<.001	-0.0014553	-0.0011684
	Parietal	-0.000154774*	4.8352E-05	<.001	-0.0002902	-1.939E-05
	Polar	-0.000709449*	7.4163E-05	<.001	-0.0009171	-0.0005018
	Granular	-0.00055056*	6.0604E-05	<.001	-0.0007203	-0.0003809
Frontal	Agranular	0.001311816*	5.1232E-05	<.001	0.00116838	0.00145526
	Parietal	0.001157042*	4.7188E-05	<.001	0.00102493	0.00128916
	Polar	0.000602367*	7.3586E-05	<.001	0.0003963	0.00080844
	Granular	0.000761256*	6.0206E-05	<.001	0.00059267	0.00092984
Parietal	Agranular	0.000154774*	4.8352E-05	<.001	1.9395E-05	0.00029015
	Frontal	-0.001157042*	4.7188E-05	<.001	-0.0012892	-0.0010249
	Polar	-0.000554676*	7.1277E-05	<.001	-0.0007543	-0.0003551
	Granular	-0.000395786*	5.7478E-05	<.001	-0.0005567	-0.0002348
Polar	Agranular	0.000709449*	7.4163E-05	<.001	0.00050176	0.00091714
	Frontal	-0.000602367*	7.3586E-05	<.001	-0.0008084	-0.0003963
	Parietal	0.000554676*	7.1277E-05	<.001	0.00035507	0.00075429
	Granular	0.000158889*	8.0076E-05	<.001	-6.535E-05	0.00038313

Granular	Agranular	0.00055056*	6.0604E-05	<.001	0.00038085	0.00072027
	Frontal	-0.000761256*	6.0206E-05	<.001	-0.0009298	-0.0005927
	Parietal	0.000395786*	5.7478E-05	<.001	0.00023483	0.00055674
	Polar	-0.000158889*	8.0076E-05	<.001	-0.0003831	6.5352E-05

*. The mean difference is significant at the 0.05 level.

Supplemental Table 28: Mean differences in temporal structure-function coupling variance across the 5 von-Economo/Koskinas-inspired cyto-architectonic classes. One-way ANOVA table with post-hoc correction for multiple comparisons (Tamhane's T2 – equal variances not assumed): Dependent variable: Temporal structure-function coupling variance; factor: Cyto-architectonic classes. Data reported in each table column were averaged across the 9 subjects scanned at Penn that passed quality-control and analyzed using our voxel-based analysis approach; the *p*-values reported under the 'Sig.' column represent the combined p_{fisher} value across the 9 subjects.

FIGURES

Supplemental Figure 1

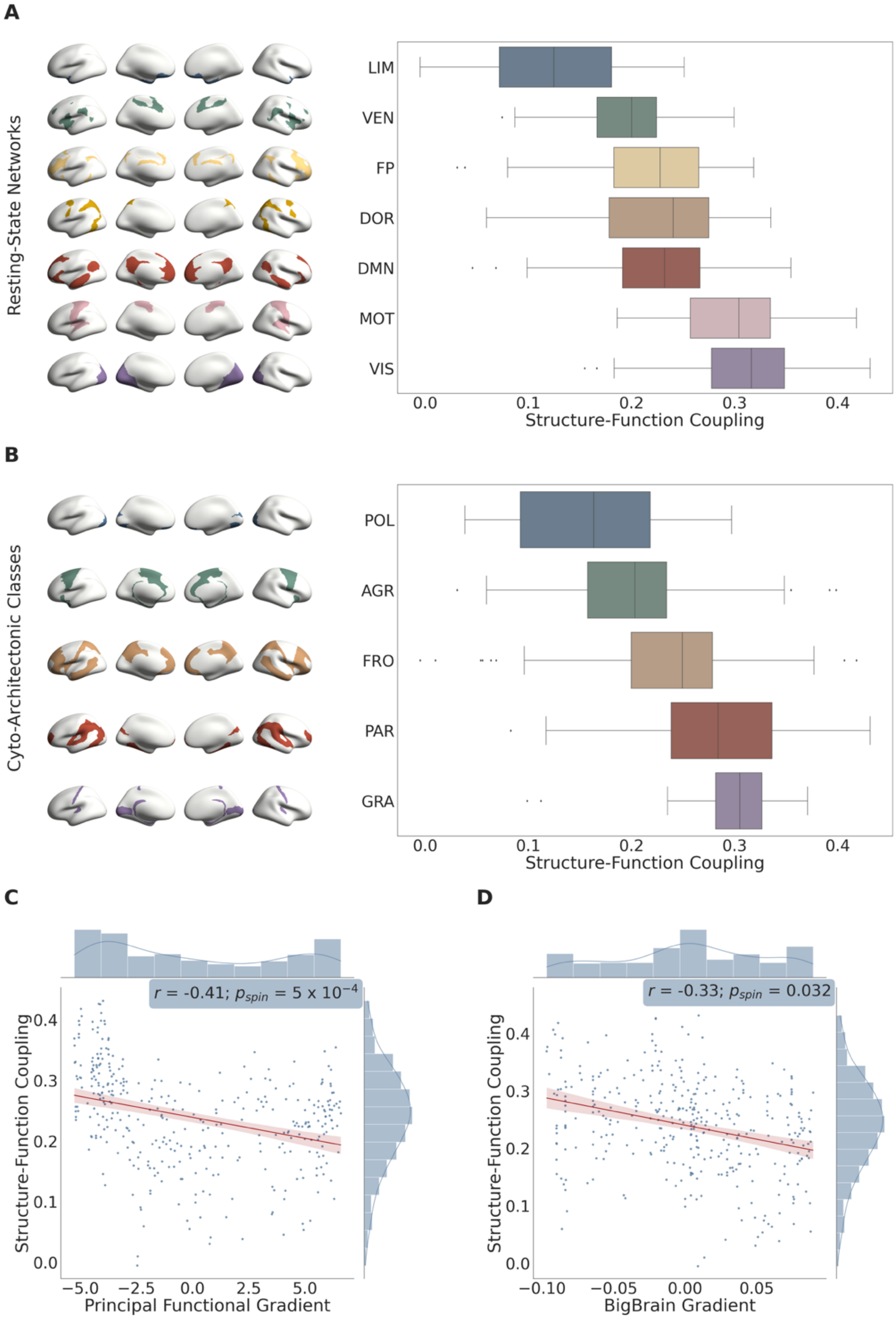

Supplemental Figure 1 – Regional variations in structure-function coupling: atlas-based analysis.
A: Mean differences in structure-function coupling across the 7 resting-state functional systems (generated using the 100 unrelated HCP subjects and HCP multi-modal atlas; *n*=360 brain regions/datapoints). Data are presented as boxplots (median value at center line, lower quartile at left bound, upper quartile at right bound) with whiskers extending towards the minimum and maximum non-outlier values of the data; single datapoints denote outliers. The brain regions in each functional system are overlayed on the standardized *fsaverage* brain's surface and illustrated on the left side. LIM: Limbic, VEN: Ventral Attention, FP: Fronto-Parietal, DMN: Default Mode Network, DOR: Dorsal Attention, MOT: Somatomotor, VIS: Visual. **B**: Mean differences in structure-function coupling across the 5 cyto-architectonic classes (generated using the 100 unrelated HCP subjects and HCP multi-modal atlas; *n*=360 brain regions/datapoints). Data are presented as boxplots (median value at center line, lower quartile at left bound, upper quartile at right bound) with whiskers extending towards the minimum and maximum non-outlier values of the data; single datapoints denote outliers. The brain regions involved within each class are overlayed on the standardized *fsaverage* brain's surface and illustrated on the left side. POL: Polar, AGR: Agranular, FRO: Frontal, PAR: Parietal, GRA: Granular. **C**: Scatterplot between the principal functional gradient scalar of each brain region and its corresponding structure-function coupling (*n*=360 brain regions/datapoints). A linear regression was fit along with a 95% confidence interval (shown in red); the correlation coefficient (two-tailed Spearman's ρ: r), p-value corresponding to the spatial permutation test (p_{spin}), and histograms corresponding to each variable are reported. **D**: Scatterplot between the "BigBrain" gradient scalar of each brain region and its corresponding structure-function coupling (*n*=360 brain regions/datapoints). A linear regression was fit along with a 95% confidence interval (shown in red); the correlation coefficient (two-tailed Spearman's ρ: r), p-value corresponding to the spatial permutation test (p_{spin}), and histograms corresponding to each variable are reported. Source data are provided as a Source Data file.

Supplemental Figure 2

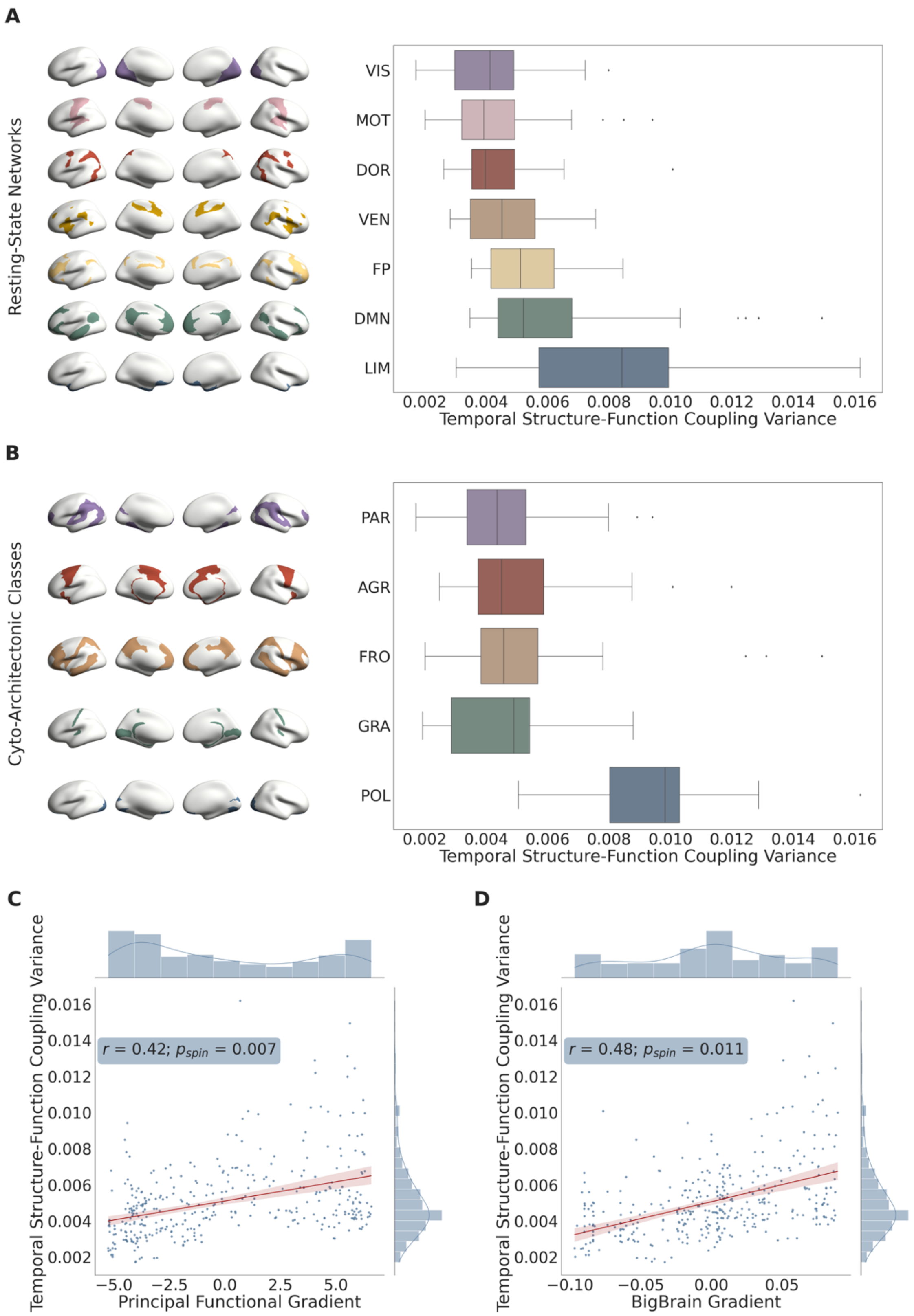

Supplemental Figure 2 – Regional variations in temporal structure-function coupling variance: atlas-based analysis.

A: Mean differences in temporal structure-function coupling variance across the 7 resting-state functional systems (generated using the 100 unrelated HCP subjects and HCP multi-modal atlas; *n*=360 brain regions/datapoints). Data are presented as boxplots (median value at center line, lower quartile at left bound, upper quartile at right bound) with whiskers extending towards the minimum and maximum non-outlier values of the data; single datapoints denote outliers. The brain regions involved within each functional system are overlayed on the standardized *fsaverage* brain's surface and illustrated on the left side. DOR: Dorsal Attention, VIS: Visual, MOT: Somatomotor, VEN: Ventral Attention, FP: Fronto-Parietal, DMN: Default Mode Network, LIM: Limbic. **B**: Mean differences in temporal structure-function coupling variance across the 5 cyto-architectonic classes (generated using the 100 unrelated HCP subjects and HCP multi-modal atlas; *n*=360 brain regions/datapoints). Data are presented as boxplots (median value at center line, lower quartile at left bound, upper quartile at right bound) with whiskers extending towards the minimum and maximum non-outlier values of the data; single datapoints denote outliers. The brain regions involved within each class are overlayed on the standardized *fsaverage* brain's surface and illustrated on the left side. PAR: Parietal, AGR: Agranular, FRO: Frontal, GRA: Granular, POL: Polar. **C**: Scatterplot between the principal functional gradient scalar of each brain region and its corresponding temporal structure-function coupling variance (*n*=360 brain regions/datapoints). A linear regression was fit along with a 95% confidence interval (shown in red); the correlation coefficient (two-tailed Spearman's ρ: r), p-value corresponding to the spatial permutation test (p_{spin}), and histograms corresponding to each variable are reported. **D**: Scatterplot between the "BigBrain" gradient scalar of each brain region and its corresponding temporal structure-function coupling variance (*n*=360 brain regions/datapoints). A linear regression was fit along with a 95% confidence interval (shown in red); the correlation coefficient (two-tailed Spearman's ρ: r), p-value corresponding to the spatial permutation test (p_{spin}), and histograms corresponding to each variable are reported. Source data are provided as a Source Data file.

Supplemental Figure 3

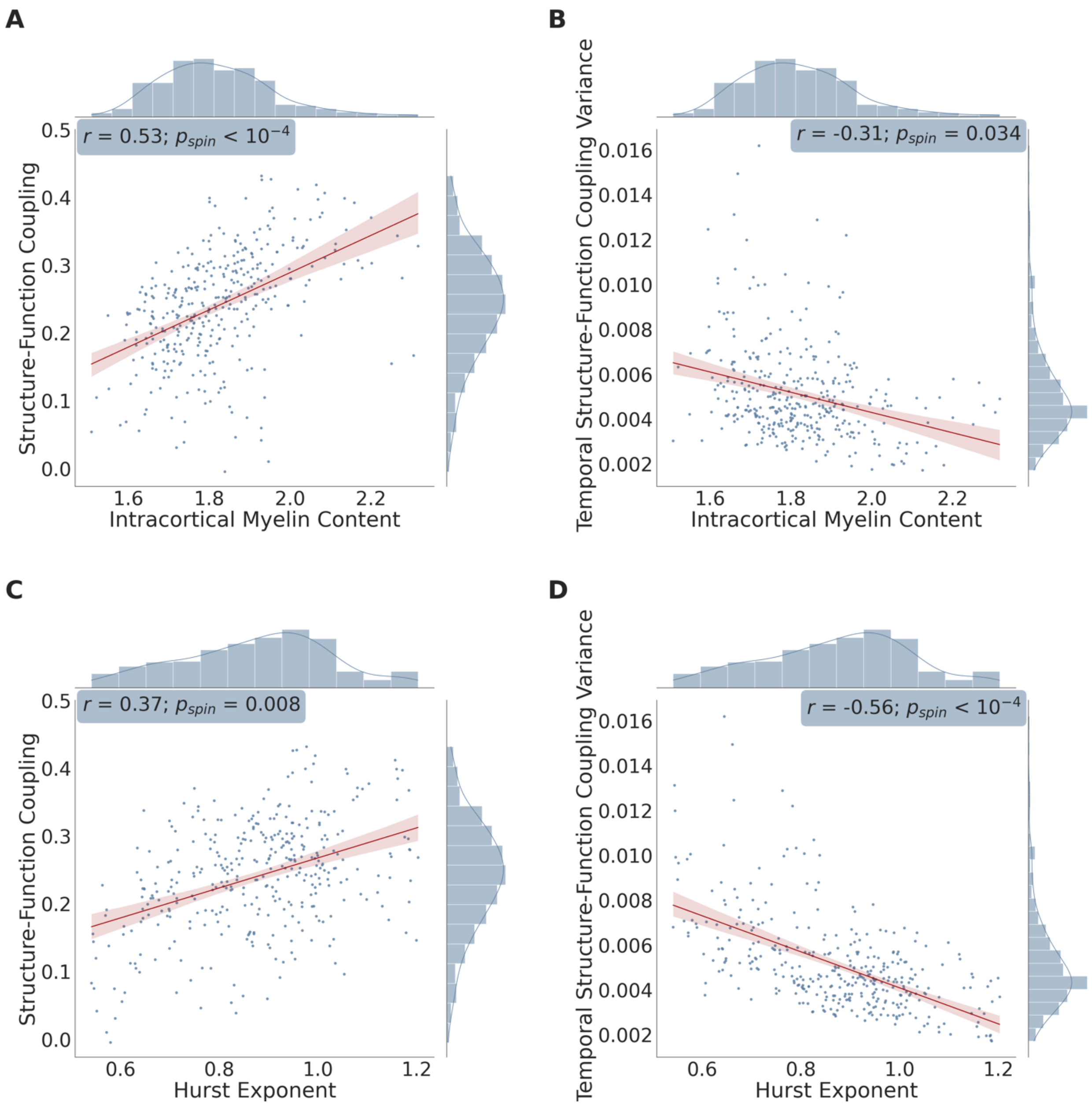

Supplemental Figure 3 – Scatterplots between the variables of interest: atlas-based analysis.
Scatterplot showing the association between each brain region's: mean structure-function coupling and intracortical myelin content as estimated by the T1-weighted/T2-weighted signal intensity ratio (**A**), mean temporal structure-function coupling variance and intracortical myelin content (**B**), mean structure-function coupling and the Hurst exponent of the functional signal time series (**C**), and mean temporal structure-function coupling variance and the Hurst exponent of the functional signal time series (**D**). For each scatterplot, a linear regression was fit along with a 95% confidence interval (shown in red); correlation coefficients (two-tailed Spearman's ρ: r), p-values corresponding to the spatial permutation test (p_{spin}), and histograms corresponding to each variable are displayed. Note: n=360 brain regions in all panels. Source data are provided as a Source Data file.

Supplemental Figure 4

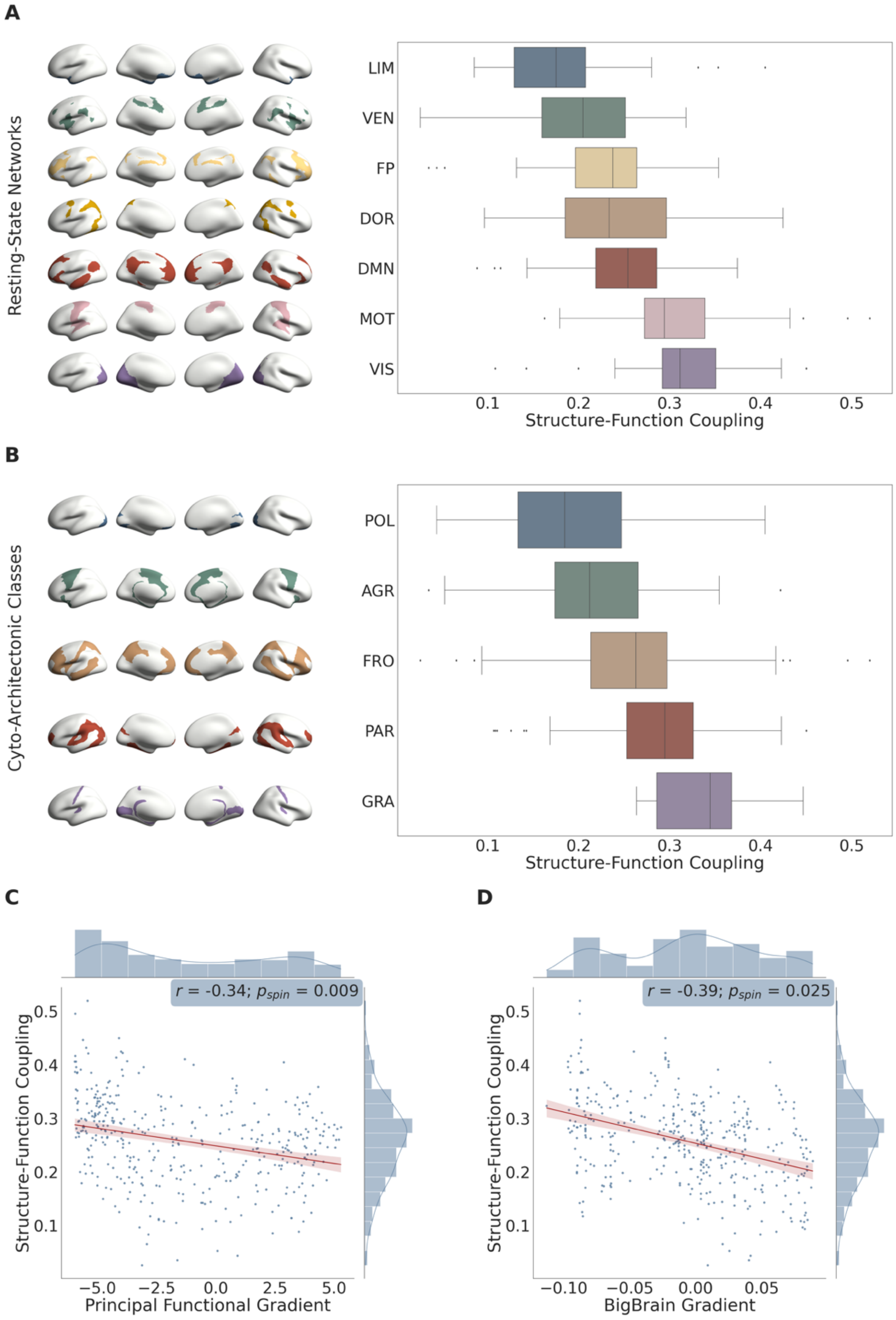

Supplemental Figure 4 – Regional variations in structure-function coupling: atlas-based analysis.
A: Mean differences in structure-function coupling across the 7 resting-state functional systems (generated using the 100 unrelated HCP subjects and Schaefer 400 atlas; n=400 brain regions/datapoints). Data are presented as boxplots (median value at center line, lower quartile at left bound, upper quartile at right bound) with whiskers extending towards the minimum and maximum non-outlier values of the data; single datapoints denote outliers. The brain regions in each functional system are overlayed on the standardized *fsaverage* brain's surface and illustrated on the left side. LIM: Limbic, VEN: Ventral Attention, FP: Fronto-Parietal, DMN: Default Mode Network, DOR: Dorsal Attention, MOT: Somatomotor, VIS: Visual. **B**: Mean differences in structure-function coupling across the 5 cyto-architectonic classes (generated using the 100 unrelated HCP subjects and Schaefer 400 atlas; n=400 brain regions/datapoints). Data are presented as boxplots (median value at center line, lower quartile at left bound, upper quartile at right bound) with whiskers extending towards the minimum and maximum non-outlier values of the data; single datapoints denote outliers. The brain regions involved within each class are overlayed on the standardized *fsaverage* brain's surface and illustrated on the left side. POL: Polar, AGR: Agranular, FRO: Frontal, PAR: Parietal, GRA: Granular. **C**: Scatterplot between the principal functional gradient scalar of each brain region and its corresponding structure-function coupling (n=400 brain regions/datapoints). A linear regression was fit along with a 95% confidence interval (shown in red); the correlation coefficient (two-tailed Spearman's ρ: r), p-value corresponding to the spatial permutation test (p_{spin}), and histograms corresponding to each variable are reported. **D**: Scatterplot between the "BigBrain" gradient scalar of each brain region and its corresponding structure-function coupling (n=400 brain regions/datapoints). A linear regression was fit along with a 95% confidence interval (shown in red); the correlation coefficient (two-tailed Spearman's ρ: r), p-value corresponding to the spatial permutation test (p_{spin}), and histograms corresponding to each variable are reported. Source data are provided as a Source Data file.

Supplemental Figure 5

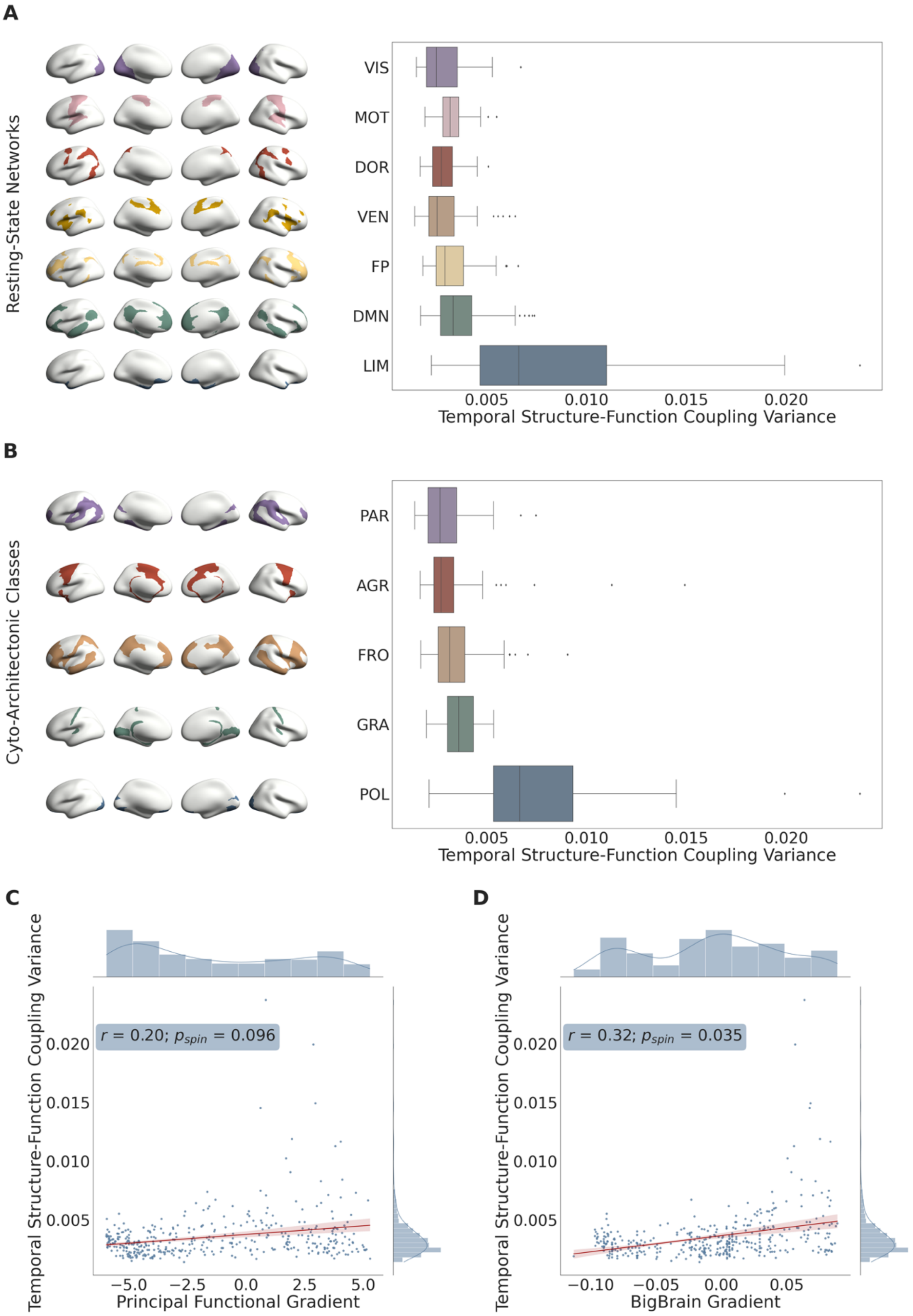

Supplemental Figure 5 – Regional variations in temporal structure-function coupling variance: atlas-based analysis.

A: Mean differences in temporal structure-function coupling variance across the 7 resting-state functional systems (generated using the 100 unrelated HCP subjects and Schaefer 400 atlas; n=400 brain regions/datapoints). Data are presented as boxplots (median value at center line, lower quartile at left bound, upper quartile at right bound) with whiskers extending towards the minimum and maximum non-outlier values of the data; single datapoints denote outliers. The brain regions involved within each functional system are overlayed on the standardized *fsaverage* brain's surface and illustrated on the left side. DOR: Dorsal Attention, VIS: Visual, MOT: Somatomotor, VEN: Ventral Attention, FP: Fronto-Parietal, DMN: Default Mode Network, LIM: Limbic. **B**: Mean differences in temporal structure-function coupling variance across the 5 cyto-architectonic classes (generated using the 100 unrelated HCP subjects and Schaefer 400 atlas; n=400 brain regions/datapoints). Data are presented as boxplots (median value at center line, lower quartile at left bound, upper quartile at right bound) with whiskers extending towards the minimum and maximum non-outlier values of the data; single datapoints denote outliers. The brain regions involved within each class are overlayed on the standardized *fsaverage* brain's surface and illustrated on the left side. PAR: Parietal, AGR: Agranular, FRO: Frontal, GRA: Granular, POL: Polar. **C**: Scatterplot between the principal functional gradient scalar of each brain region and its corresponding temporal structure-function coupling variance (n=400 brain regions/datapoints). A linear regression was fit along with a 95% confidence interval (shown in red); the correlation coefficient (two-tailed Spearman's ρ: r), p-value corresponding to the spatial permutation test (p_{spin}), and histograms corresponding to each variable are reported. **D**: Scatterplot between the "BigBrain" gradient scalar of each brain region and its corresponding temporal structure-function coupling variance (n=400 brain regions/datapoints). A linear regression was fit along with a 95% confidence interval (shown in red); the correlation coefficient (two-tailed Spearman's ρ: r), p-value corresponding to the spatial permutation test (p_{spin}), and histograms corresponding to each variable are reported. Source data are provided as a Source Data file.

Supplemental Figure 6

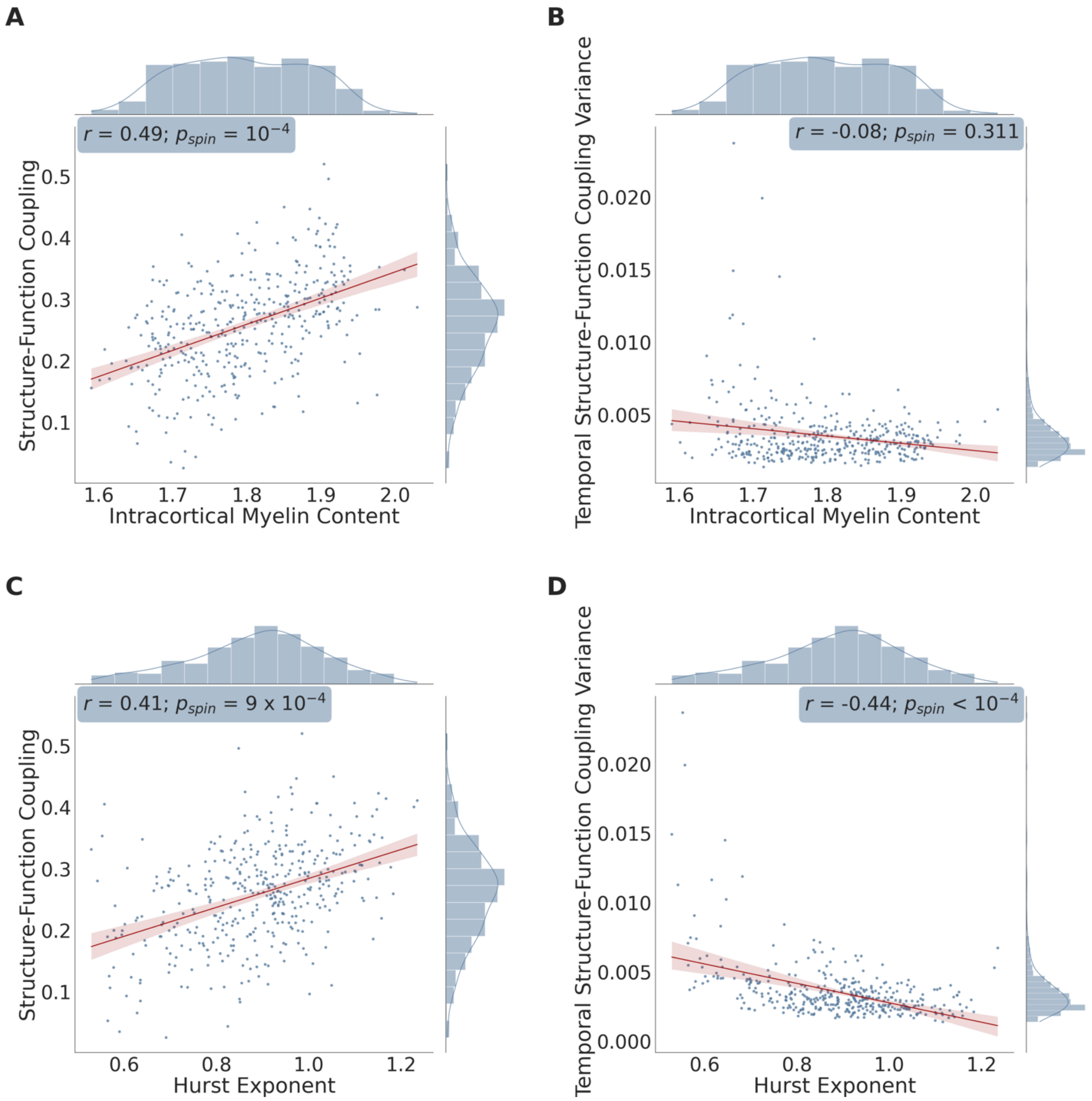

Supplemental Figure 6 – Scatterplots between the variables of interest: atlas-based analysis.
Scatterplot showing the association between each brain region's: mean structure-function coupling and intracortical myelin content as estimated by the T1-weighted/T2-weighted signal intensity ratio (**A**), mean temporal structure-function coupling variance and intracortical myelin content (**B**), mean structure-function coupling and the Hurst exponent of the functional signal time series (**C**), and mean temporal structure-function coupling variance and the Hurst exponent of the functional signal time series (**D**). For each scatterplot, a linear regression was fit along with a 95% confidence interval (shown in red); correlation coefficients (two-tailed Spearman's ρ: r), p-values corresponding to the spatial permutation test (p_{spin}), and histograms corresponding to each variable are displayed. Note: n=400 brain regions in all panels. Source data are provided as a Source Data file.

Supplemental Figure 7

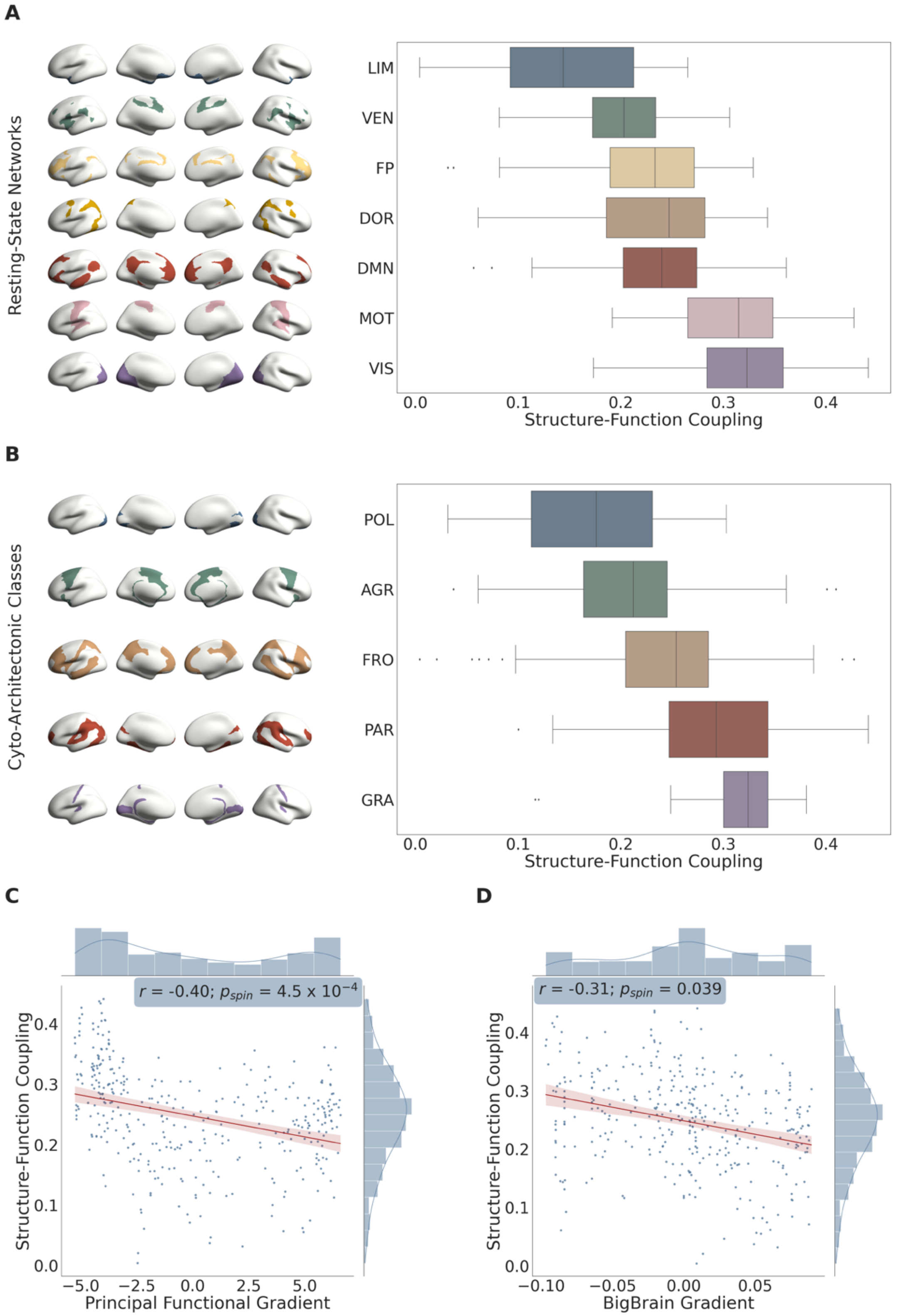

Supplemental Figure 7 – Regional variations in structure-function coupling: atlas-based analysis.
A: Mean differences in structure-function coupling across the 7 resting-state functional systems (generated using the 100 unrelated HCP subjects and HCP multi-modal atlas; n=360 brain regions/datapoints). Data are presented as boxplots (median value at center line, lower quartile at left bound, upper quartile at right bound) with whiskers extending towards the minimum and maximum non-outlier values of the data; single datapoints denote outliers. The brain regions in each functional system are overlayed on the standardized *fsaverage* brain's surface and illustrated on the left side. LIM: Limbic, VEN: Ventral Attention, FP: Fronto-Parietal, DMN: Default Mode Network, DOR: Dorsal Attention, MOT: Somatomotor, VIS: Visual. **B**: Mean differences in structure-function coupling across the 5 cyto-architectonic classes (generated using the 100 unrelated HCP subjects and HCP multi-modal atlas; n=360 brain regions/datapoints). Data are presented as boxplots (median value at center line, lower quartile at left bound, upper quartile at right bound) with whiskers extending towards the minimum and maximum non-outlier values of the data; single datapoints denote outliers. The brain regions involved within each class are overlayed on the standardized *fsaverage* brain's surface and illustrated on the left side. POL: Polar, AGR: Agranular, FRO: Frontal, PAR: Parietal, GRA: Granular. **C**: Scatterplot between the principal functional gradient scalar of each brain region and its corresponding structure-function coupling (n=360 brain regions/datapoints). A linear regression was fit along with a 95% confidence interval (shown in red); the correlation coefficient (two-tailed Spearman's ρ: r), p-value corresponding to the spatial permutation test (p_{spin}), and histograms corresponding to each variable are reported. **D**: Scatterplot between the "BigBrain" gradient scalar of each brain region and its corresponding structure-function coupling (n=360 brain regions/datapoints). A linear regression was fit along with a 95% confidence interval (shown in red); the correlation coefficient (two-tailed Spearman's ρ: r), p-value corresponding to the spatial permutation test (p_{spin}), and histograms corresponding to each variable are reported. Source data are provided as a Source Data file.

Supplemental Figure 8

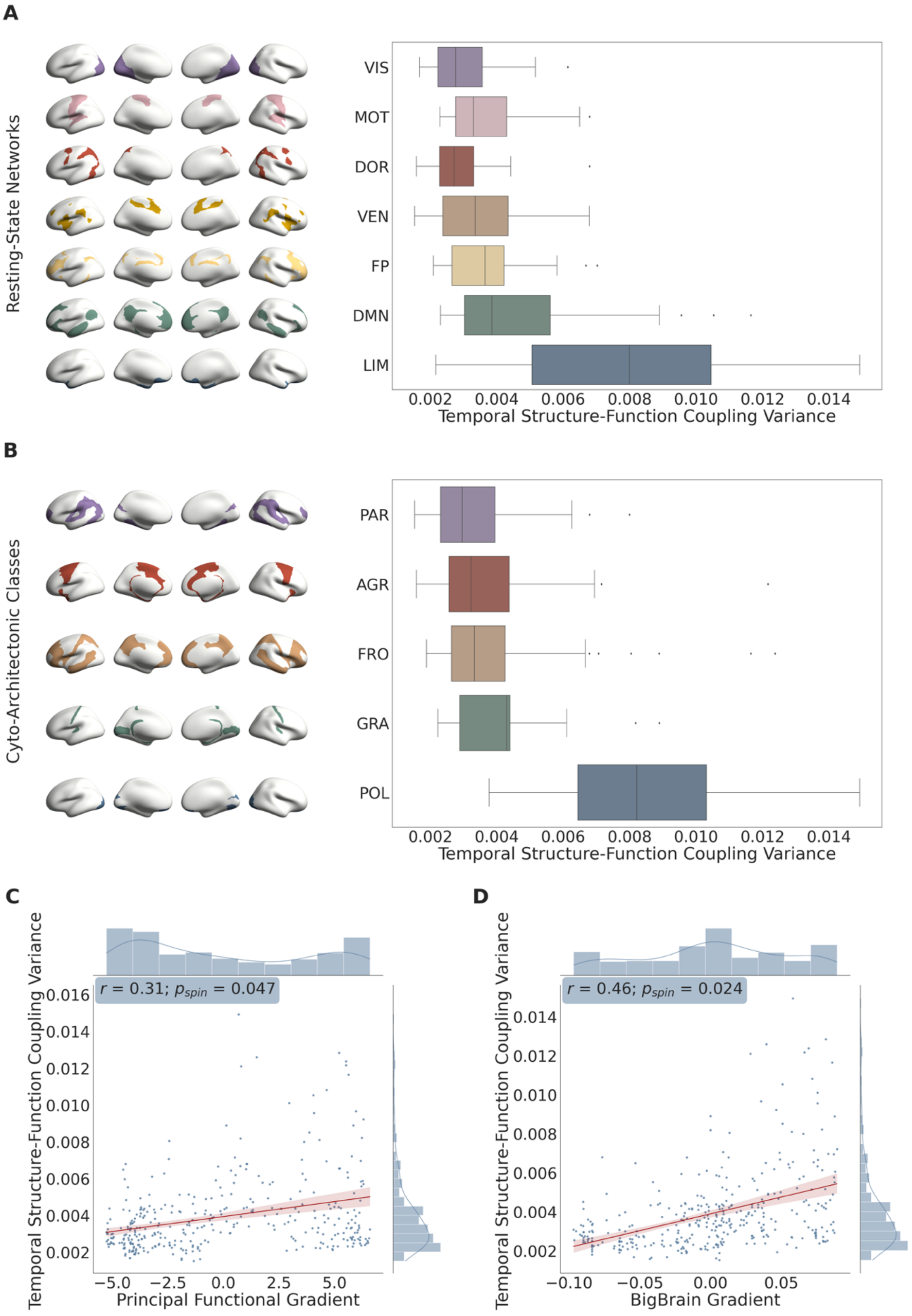

Supplemental Figure 8 – Regional variations in temporal structure-function coupling variance: atlas-based analysis.

A: Mean differences in temporal structure-function coupling variance across the 7 resting-state functional systems (generated using the 100 unrelated HCP subjects and HCP multi-modal atlas; *n*=360 brain regions/datapoints). Data are presented as boxplots (median value at center line, lower quartile at left bound, upper quartile at right bound) with whiskers extending towards the minimum and maximum non-outlier values of the data; single datapoints denote outliers. The brain regions involved within each functional system are overlayed on the standardized *fsaverage* brain's surface and illustrated on the left side. DOR: Dorsal Attention, VIS: Visual, MOT: Somatomotor, VEN: Ventral Attention, FP: Fronto-Parietal, DMN: Default Mode Network, LIM: Limbic. **B**: Mean differences in temporal structure-function coupling variance across the 5 cyto-architectonic classes (generated using the 100 unrelated HCP subjects and HCP multi-modal atlas; *n*=360 brain regions/datapoints). Data are presented as boxplots (median value at center line, lower quartile at left bound, upper quartile at right bound) with whiskers extending towards the minimum and maximum non-outlier values of the data; single datapoints denote outliers. The brain regions involved within each class are overlayed on the standardized *fsaverage* brain's surface and illustrated on the left side. PAR: Parietal, AGR: Agranular, FRO: Frontal, GRA: Granular, POL: Polar. **C**: Scatterplot between the principal functional gradient scalar of each brain region and its corresponding temporal structure-function coupling variance (*n*=360 brain regions/datapoints). A linear regression was fit along with a 95% confidence interval (shown in red); the correlation coefficient (two-tailed Spearman's ρ: r), p-value corresponding to the spatial permutation test (p_{spin}), and histograms corresponding to each variable are reported. **D**: Scatterplot between the "BigBrain" gradient scalar of each brain region and its corresponding temporal structure-function coupling variance (*n*=360 brain regions/datapoints). A linear regression was fit along with a 95% confidence interval (shown in red); the correlation coefficient (two-tailed Spearman's ρ: r), p-value corresponding to the spatial permutation test (p_{spin}), and histograms corresponding to each variable are reported. Source data are provided as a Source Data file.

Supplemental Figure 9

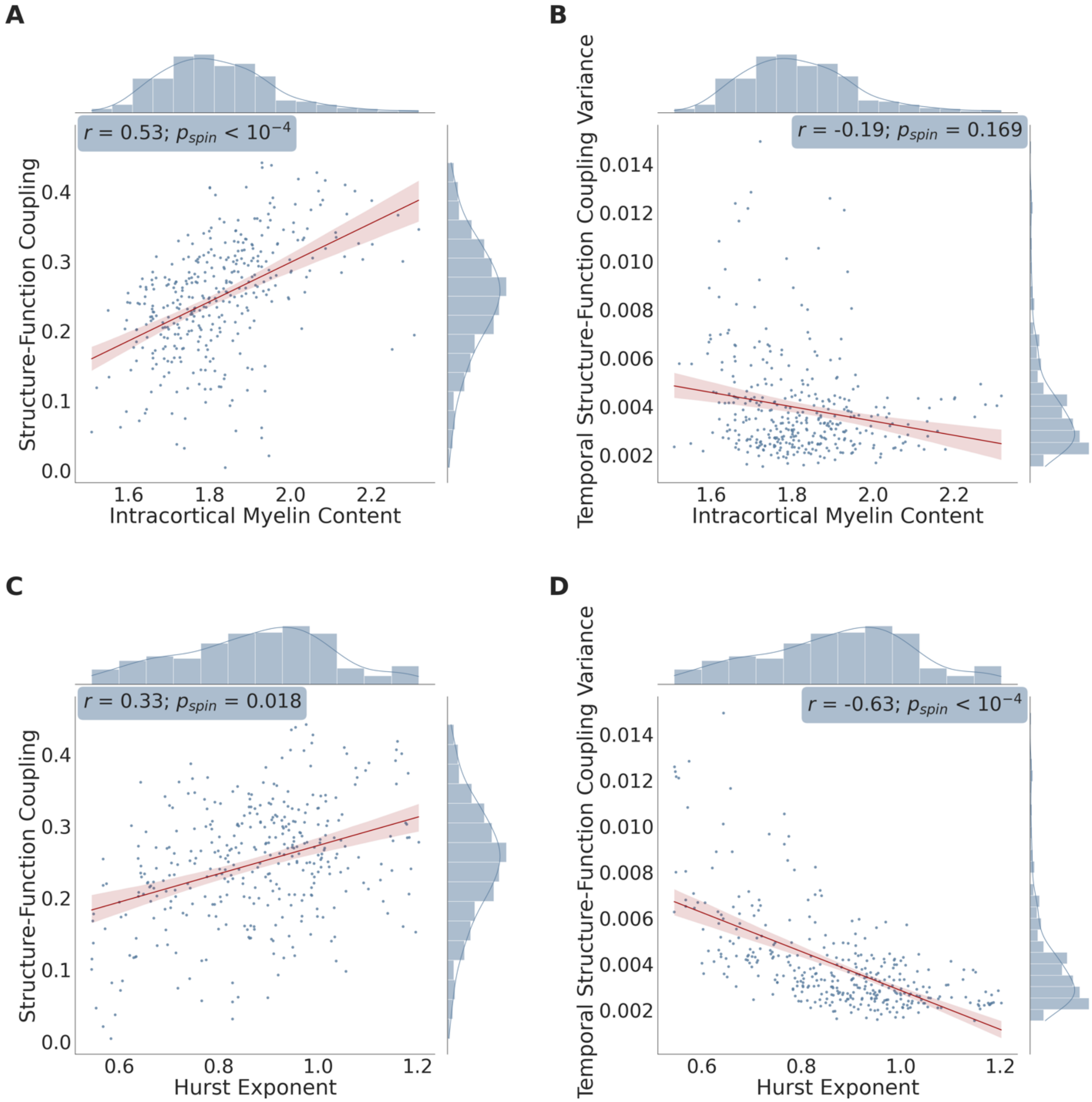

Supplemental Figure 9 – Scatterplots between the variables of interest: atlas-based analysis.

Scatterplot showing the association between each brain region's: mean structure-function coupling and intracortical myelin content as estimated by the T1-weighted/T2-weighted signal intensity ratio (**A**), mean temporal structure-function coupling variance and intracortical myelin content (**B**), mean structure-function coupling and the Hurst exponent of the functional signal time series (**C**), and mean temporal structure-function coupling variance and the Hurst exponent of the functional signal time series (**D**). For each scatterplot, a linear regression was fit along with a 95% confidence interval (shown in red); correlation coefficients (two-tailed Spearman's ρ: r), p-values corresponding to the spatial permutation test (p_{spin}), and histograms corresponding to each variable are displayed. Note: n=360 brain regions in all panels. Source data are provided as a Source Data file.

Supplemental Figure 10

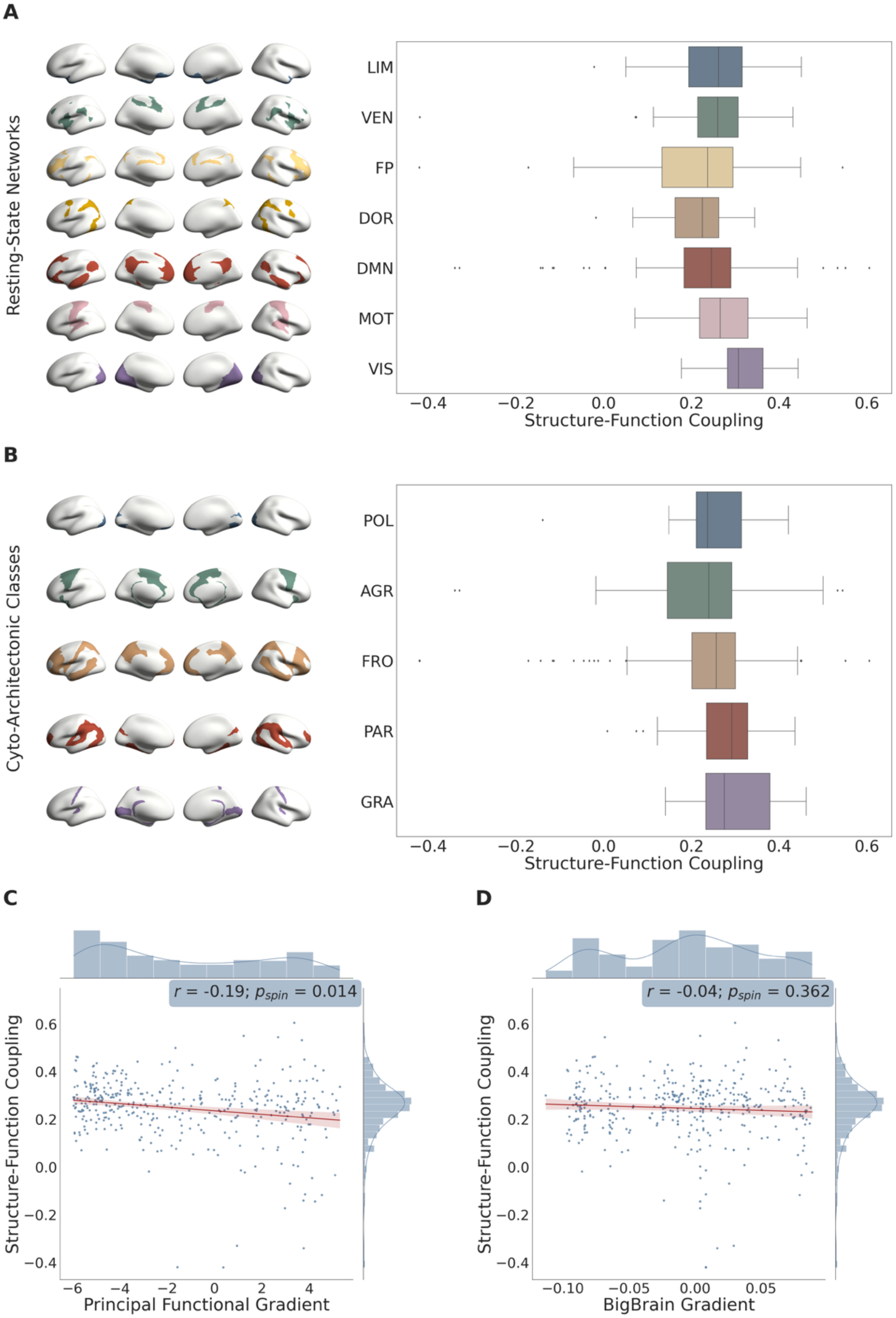

Supplemental Figure 10 – Regional variations in structure-function coupling: atlas-based analysis.
A: Mean differences in structure-function coupling across the 7 resting-state functional systems (generated using the 14 Penn subjects and Schaefer cortical atlas; n=400 brain regions/datapoints). Data are presented as boxplots (median value at center line, lower quartile at left bound, upper quartile at right bound) with whiskers extending towards the minimum and maximum non-outlier values of the data; single datapoints denote outliers. The brain regions involved within each functional system are overlayed on the standardized *fsaverage* brain's surface and illustrated on the left side. DOR: Dorsal Attention, VIS: Visual, MOT: Somatomotor, VEN: Ventral Attention, FP: Fronto-Parietal, DMN: Default Mode Network, LIM: Limbic. **B**: Mean differences in structure-function coupling across the 5 cyto-architectonic classes (generated using the 14 Penn subjects and Schaefer cortical atlas; n=400 brain regions/datapoints). Data are presented as boxplots (median value at center line, lower quartile at left bound, upper quartile at right bound) with whiskers extending towards the minimum and maximum non-outlier values of the data; single datapoints denote outliers. The brain regions involved within each class are overlayed on the standardized *fsaverage* brain's surface and illustrated on the left side. PAR: Parietal, AGR: Agranular, FRO: Frontal, GRA: Granular, POL: Polar. **C**: Scatterplot between the principal functional gradient scalar of each brain region and its corresponding structure-function coupling (n=400 brain regions/datapoints). A linear regression was fit along with a 95% confidence interval (shown in red); the correlation coefficient (two-tailed Spearman's ρ: r), p-value corresponding to the spatial permutation test (p_{spin}), and histograms corresponding to each variable are reported. **D**: Scatterplot between the "BigBrain" gradient scalar of each brain region and its corresponding structure-function coupling (n=400 brain regions/datapoints). A linear regression was fit along with a 95% confidence interval (shown in red); the correlation coefficient (two-tailed Spearman's ρ: r), p-value corresponding to the spatial permutation test (p_{spin}), and histograms corresponding to each variable are reported. Source data are provided as a Source Data file.

Supplemental Figure 11

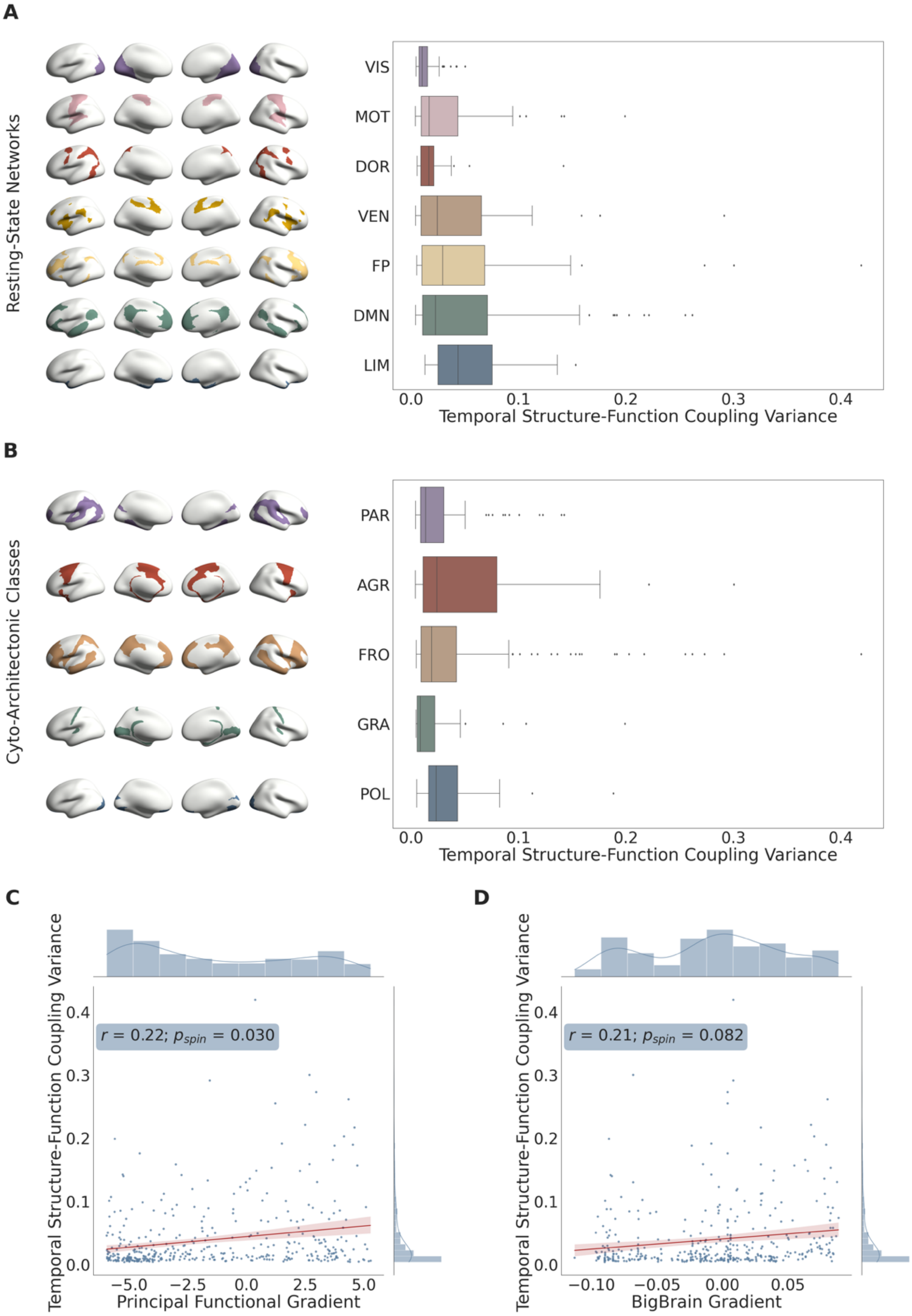

Supplemental Figure 11 – Regional variations in temporal structure-function coupling variance: atlas-based analysis.

A: Mean differences in temporal structure-function coupling variance across the 7 resting-state functional systems (generated using the 14 Penn subjects and Schaefer cortical atlas; n=400 brain regions/datapoints). Data are presented as boxplots (median value at center line, lower quartile at left bound, upper quartile at right bound) with whiskers extending towards the minimum and maximum non-outlier values of the data; single datapoints denote outliers. The brain regions involved within each functional system are overlayed on the standardized *fsaverage* brain's surface and illustrated on the left side. DOR: Dorsal Attention, VIS: Visual, MOT: Somatomotor, VEN: Ventral Attention, FP: Fronto-Parietal, DMN: Default Mode Network, LIM: Limbic. **B**: Mean differences in temporal structure-function coupling variance across the 5 cyto-architectonic classes (generated using the 14 Penn subjects and Schaefer cortical atlas; n=400 brain regions/datapoints). Data are presented as boxplots (median value at center line, lower quartile at left bound, upper quartile at right bound) with whiskers extending towards the minimum and maximum non-outlier values of the data; single datapoints denote outliers. The brain regions involved within each class are overlayed on the standardized *fsaverage* brain's surface and illustrated on the left side. PAR: Parietal, AGR: Agranular, FRO: Frontal, GRA: Granular, POL: Polar. **C**: Scatterplot between the principal functional gradient scalar of each brain region and its corresponding temporal structure-function coupling variance (n=400 brain regions/datapoints). A linear regression was fit along with a 95% confidence interval (shown in red); the correlation coefficient (two-tailed Spearman's ρ: r), p-value corresponding to the spatial permutation test (p_{spin}), and histograms corresponding to each variable are reported. **D**: Scatterplot between the "BigBrain" gradient scalar of each brain region and its corresponding temporal structure-function coupling variance (n=400 brain regions/datapoints). A linear regression was fit along with a 95% confidence interval (shown in red); the correlation coefficient (two-tailed Spearman's ρ: r), p-value corresponding to the spatial permutation test (p_{spin}), and histograms corresponding to each variable are reported. Source data are provided as a Source Data file.

Supplemental Figure 12

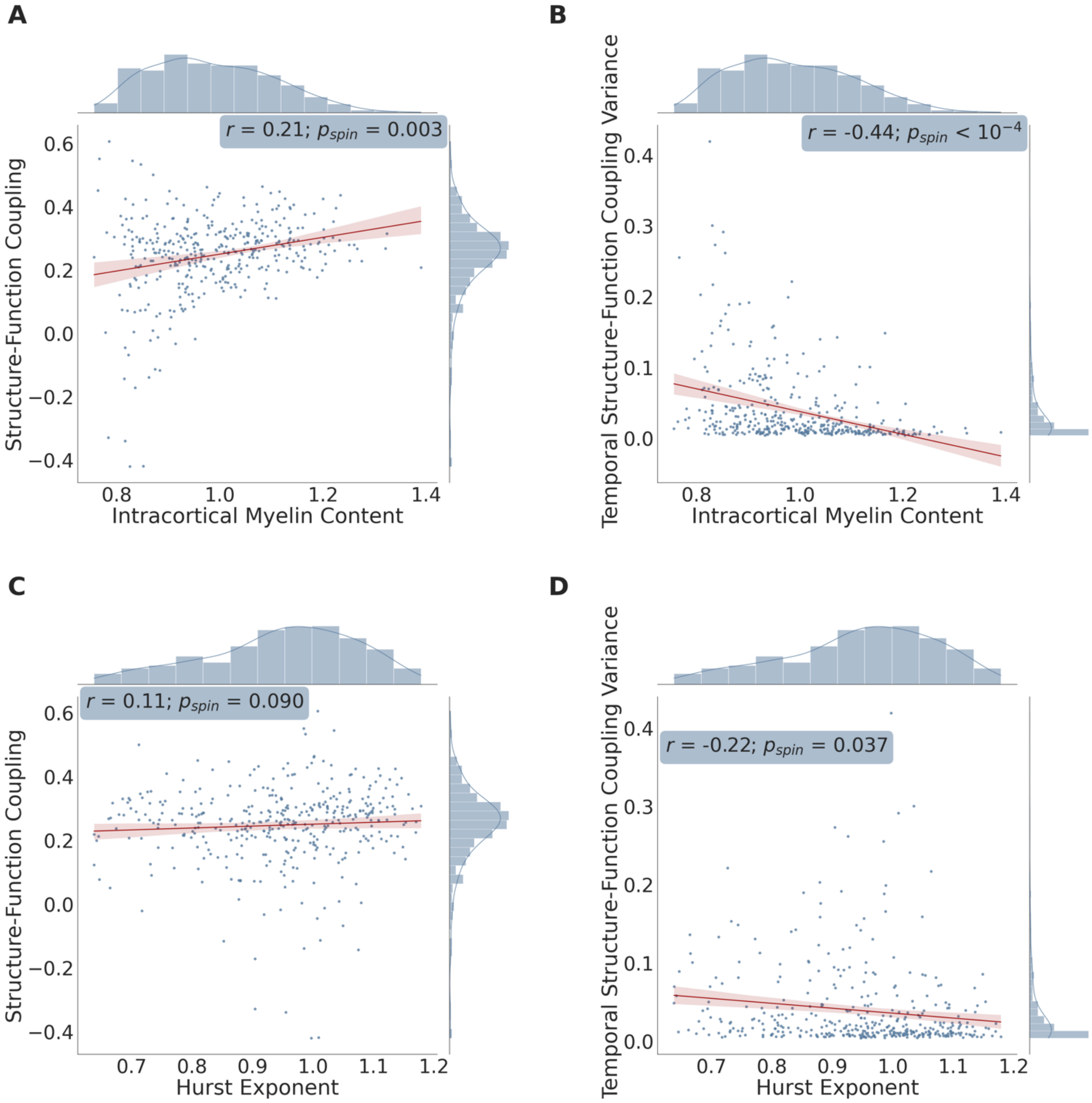

Supplemental Figure 12 – Scatterplots between the variables of interest: atlas-based analysis.
Scatterplot showing the association between each brain region's: mean structure-function coupling and intracortical myelin content as estimated by the T1-weighted/T2-weighted signal intensity ratio (**A**), mean temporal structure-function coupling variance and intracortical myelin content (**B**), mean structure-function coupling and the Hurst exponent of the functional signal time series (**C**), and mean temporal structure-function coupling variance and the Hurst exponent of the functional signal time series (**D**). For each scatterplot, a linear regression was fit along with a 95% confidence interval (shown in red); correlation coefficients (two-tailed Spearman's ρ: r), p-values corresponding to the spatial permutation test (p_{spin}), and histograms corresponding to each variable are displayed. Note: n=400 brain regions in all panels. Source data are provided as a Source Data file.

Supplemental Figure 13

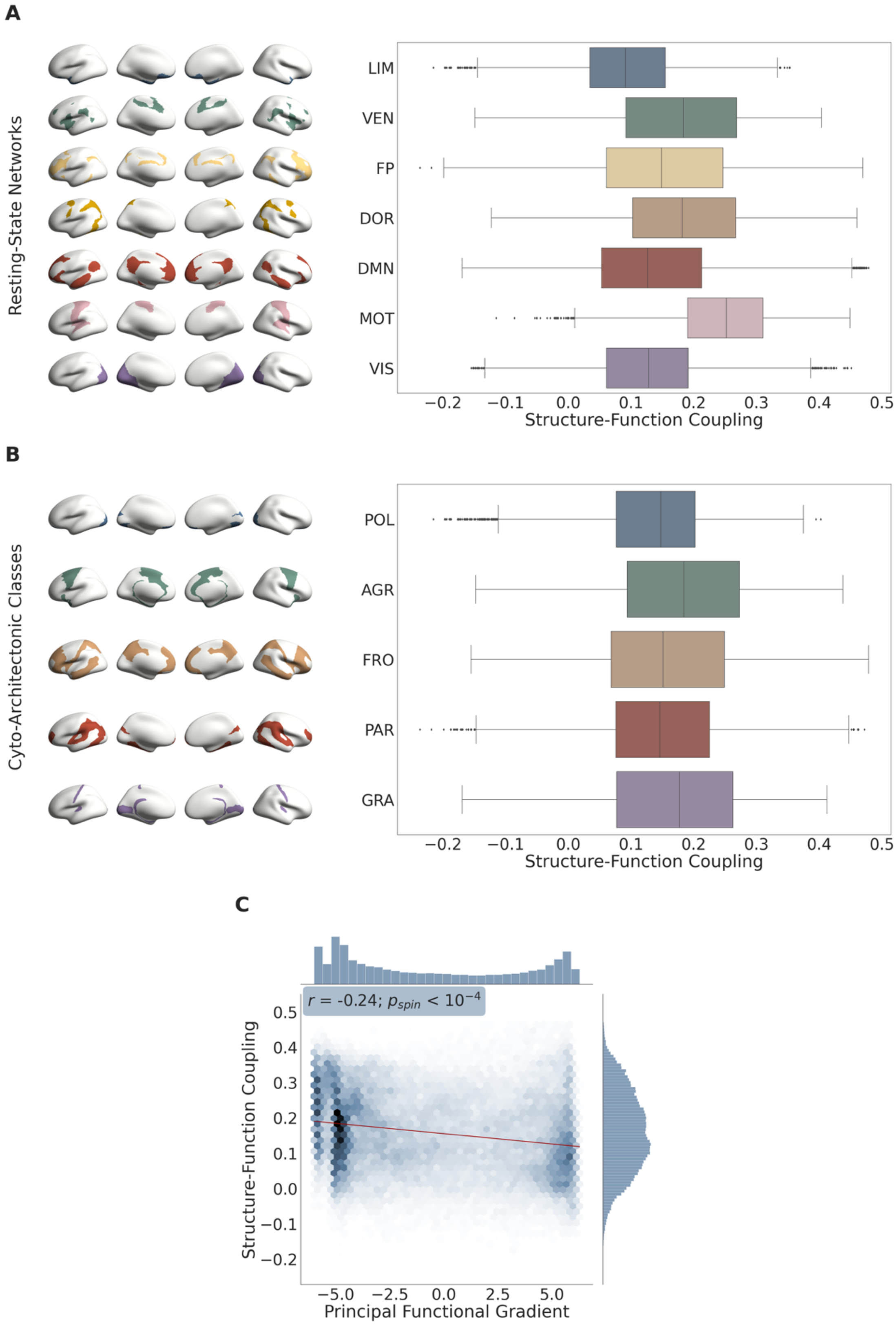

Supplemental Figure 13 – Regional variations in structure-function coupling: voxel-based analysis – representative subject shown.

A: Mean differences in structure-function coupling across the 7 resting-state functional networks (generated using one representative Penn subject analyzed with our voxel-based connectivity approach; *n*=71,561 brain regions/datapoints). Data are presented as boxplots (median value at center line, lower quartile at left bound, upper quartile at right bound) with whiskers extending towards the minimum and maximum non-outlier values of the data; single datapoints denote outliers. The brain regions involved within each functional network are overlayed on the standardized *fsaverage* brain's surface and illustrated on the left side. LIM: Limbic, VEN: Ventral Attention, FP: Fronto-Parietal, DMN: Default Mode Network, DOR: Dorsal Attention, MOT: Somatomotor, VIS: Visual. **B**: Mean differences in structure-function coupling across the 5 cyto-architectonic classes (generated using one representative Penn subject analyzed with our voxel-based connectivity approach; *n*=71,561 brain regions/datapoints). Data are presented as boxplots (median value at center line, lower quartile at left bound, upper quartile at right bound) with whiskers extending towards the minimum and maximum non-outlier values of the data; single datapoints denote outliers. The brain regions involved within each class are overlayed on the standardized *fsaverage* brain's surface and illustrated on the left side. POL: Polar, AGR: Agranular, FRO: Frontal, PAR: Parietal, GRA: Granular. **C**: High density plot between the principal functional gradient scalar of each brain region and its corresponding structure-function coupling (*n*=71,561 brain regions/datapoints). A linear regression was fit along with a 95% confidence interval (shown in red); the correlation coefficient (two-tailed Spearman's ρ: r), p-value corresponding to the spatial permutation test (p_{spin}), and histograms corresponding to each variable are displayed. Source data are provided as a Source Data file.

Supplemental Figure 14

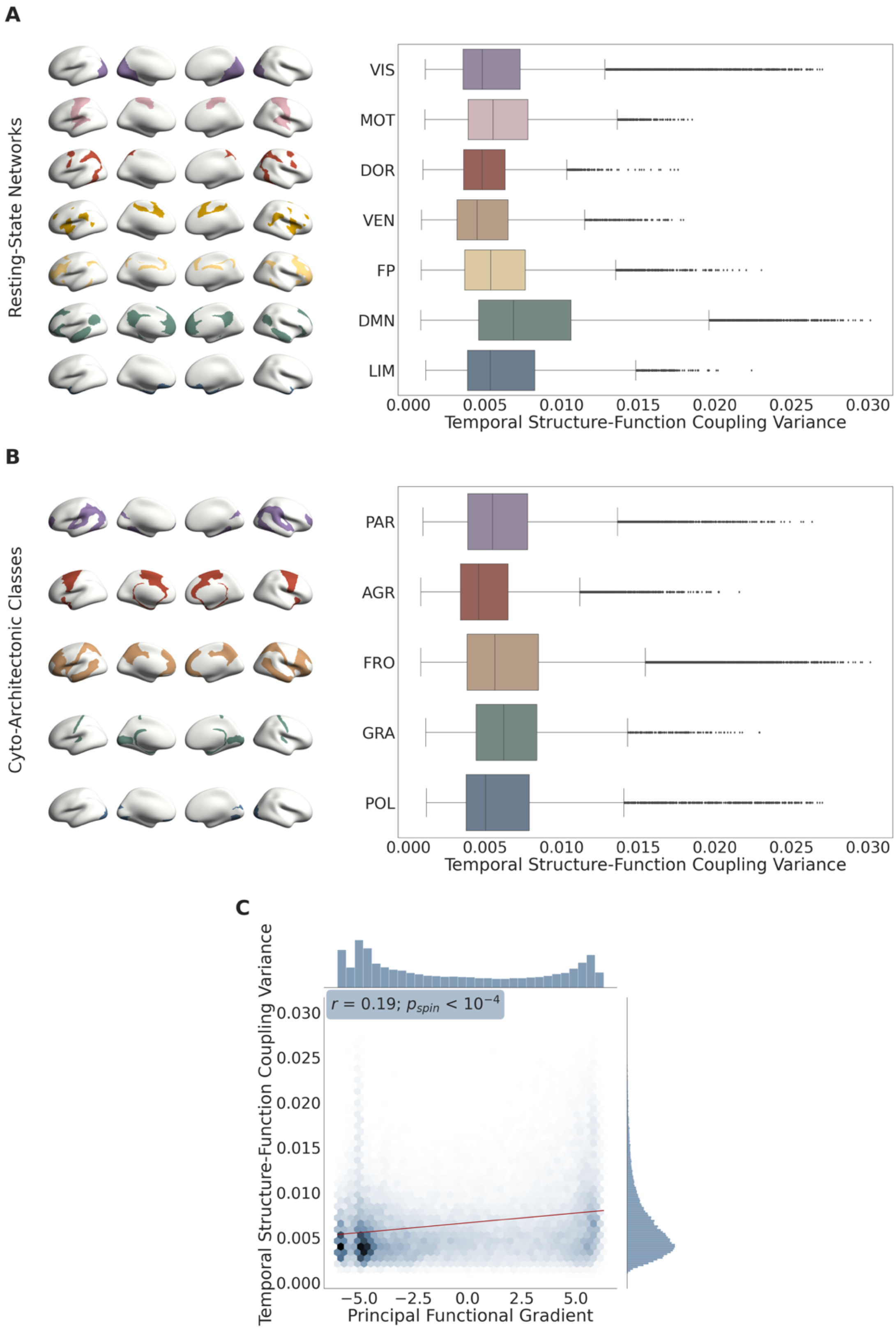

Supplemental Figure 14 – Regional variations in temporal structure-function coupling variance: voxel-based analysis – representative subject shown.

A: Mean differences in temporal structure-function coupling variance across the 7 resting-state functional networks (generated using one representative Penn subject analyzed with our voxel-based connectivity approach; *n*=71,561 brain regions/datapoints). Data are presented as boxplots (median value at center line, lower quartile at left bound, upper quartile at right bound) with whiskers extending towards the minimum and maximum non-outlier values of the data; single datapoints denote outliers. The brain regions involved within each functional network are overlayed on the standardized *fsaverage* brain's surface and illustrated on the left side. DOR: Dorsal Attention, VIS: Visual, MOT: Somatomotor, VEN: Ventral Attention, FP: Fronto-Parietal, DMN: Default Mode Network, LIM: Limbic. **B**: Mean differences in temporal structure-function coupling variance across the 5 cyto-architectonic classes (generated using one representative Penn subject analyzed with our voxel-based connectivity approach; *n*=71,561 brain regions/datapoints). Data are presented as boxplots (median value at center line, lower quartile at left bound, upper quartile at right bound) with whiskers extending towards the minimum and maximum non-outlier values of the data; single datapoints denote outliers. The brain regions involved within each class are overlayed on the standardized *fsaverage* brain's surface and illustrated on the left side. PAR: Parietal, AGR: Agranular, FRO: Frontal, GRA: Granular, POL: Polar. **C**: High density plot between the principal functional gradient scalar of each brain region and its corresponding temporal structure-function coupling variance (*n*=71,561 brain regions/datapoints). A linear regression was fit along with a 95% confidence interval (shown in red); the correlation coefficient (two-tailed Spearman's ρ: r), p-value corresponding to the spatial permutation test (p_{spin}), and histograms corresponding to each variable are displayed. Source data are provided as a Source Data file.

Supplemental Figure 15

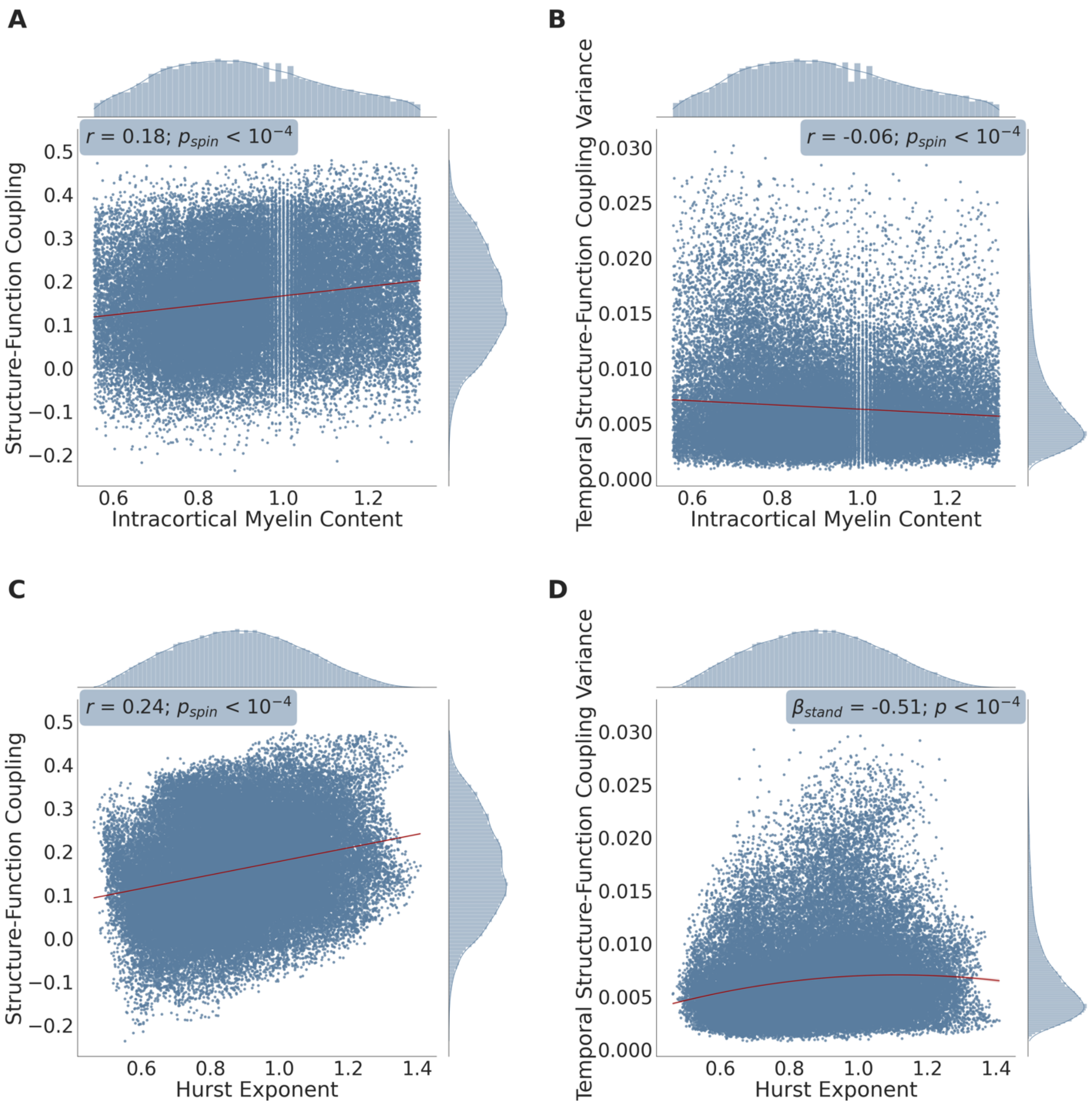

Supplemental Figure 15 – Scatterplots between the variables of interest: voxel-based analysis – representative subject shown.

Scatterplots showing the association between each cortical voxel's: mean structure-function coupling and intracortical myelin content estimated by the T1-weighted/T2-weighted signal intensity ratio (**A**), mean temporal structure-function coupling variance and intracortical myelin content (**B**), mean structure-function coupling and the Hurst exponent of the functional signal time series (**C**), and mean temporal structure-function coupling variance and the Hurst exponent of the functional signal time series (**D**). For plots (A), (B), and (C), a linear regression was fit along with a 95% confidence interval (shown in red); correlation coefficients (two-tailed Spearman's ρ: r), p-values corresponding to the spatial permutation test (p_{spin}), and histograms corresponding to each variable are displayed. In plot (D), a quadratic regression was fit along with a 95% confidence interval (shown in red); the standardized β coefficient and bootstrapped p-value corresponding to the quadratic regression mentioned in the voxel-based analysis component of our Results section: 'Biological Correlates of Structure-Function Coupling: Whole-brain perspective,' are also reported. Data shown in this figure were obtained from a representative subject that was analyzed using our voxel-based connectivity approach. Note: n=71,561 voxels in all panels. Source data are provided as a Source Data file.

SUPPLEMENTARY REFERENCES

1. Glasser, M. F. & Van Essen, D. C. Mapping Human Cortical Areas In Vivo Based on Myelin Content as Revealed by T1- and T2-Weighted MRI. *J. Neurosci.* **31**, 11597–11616 (2011).
2. Ganzetti, M., Wenderoth, N. & Mantini, D. Whole brain myelin mapping using T1- and T2-weighted MR imaging data. *Front. Hum. Neurosci.* **8**, 671 (2014).
3. Ganzetti, M., Wenderoth, N. & Mantini, D. Mapping pathological changes in brain structure by combining T1- and T2-weighted MR imaging data. *Neuroradiology* **57**, 917–928 (2015).
4. Nerland, S. *et al.* Multisite reproducibility and test-retest reliability of the T1w/T2w-ratio: A comparison of processing methods. *NeuroImage* **245**, 118709 (2021).
5. Glasser, M. F. *et al.* Empirical transmit field bias correction of T1w/T2w myelin maps. *NeuroImage* **258**, 119360 (2022).
6. Arshad, M., Stanley, J. A. & Raz, N. Test–retest reliability and concurrent validity of in vivo myelin content indices: Myelin water fraction and calibrated T1w/T2w image ratio. *Hum. Brain Mapp.* **38**, 1780–1790 (2016).
7. Shams, Z., Norris, D. G. & Marques, J. P. A comparison of in vivo MRI based cortical myelin mapping using T1w/T2w and R1 mapping at 3T. *PLOS ONE* **14**, e0218089 (2019).
8. Uddin, M. N., Figley, T. D., Marrie, R. A., Figley, C. R. & for the CCOMS Study Group. Can T1w/T2w ratio be used as a myelin-specific measure in subcortical structures? Comparisons between FSE-based T1w/ T2w ratios, GRASE-based T1w/T2w ratios and multi-echo GRASE-based myelin water fractions. *NMR Biomed.* **31**, e3868 (2018).
9. Hagiwara, A. *et al.* Myelin Measurement: Comparison Between Simultaneous Tissue Relaxometry, Magnetization Transfer Saturation Index, and T1w/T2w Ratio Methods. *Sci. Rep.* **8**, 10554 (2018).

10. Piredda, G. F., Hilbert, T., Thiran, J.-P. & Kober, T. Probing myelin content of the human brain with MRI: A review. *Magn. Reson. Med.* **85**, 627–652 (2021).

11. Trakoshis, S. *et al.* Intrinsic excitation-inhibition imbalance affects medial prefrontal cortex differently in autistic men versus women. *eLife* **9**, e55684 (2020).

12. Gao, R., Peterson, E. J. & Voytek, B. Inferring synaptic excitation/inhibition balance from field potentials. *NeuroImage* **158**, 70–78 (2017).

13. Larsen, B. *et al.* A developmental reduction of the excitation:inhibition ratio in association cortex during adolescence. *Sci. Adv.* **8**, eabj8750 (2022).
