# Supplementary material for: Myelination and excitation-inhibition balance synergistically shape structure-function coupling across the human cortex
Source: Nat Commun. 2023 Sep 30;14:6115. doi: 10.1038/s41467-023-41686-9 (PMC10542365; doi:10.1038/s41467-023-41686-9)
Supplement: Supplementary file 3 — Reporting Summary [file 41467_2023_41686_MOESM3_ESM.pdf]

nature portfolio

Corresponding author(s): Panagiotis Fotiadis & Dani S. Bassett

Last updated by author(s): Aug 22, 2023

Reporting Summary

Nature Portfolio wishes to improve the reproducibility of the work that we publish. This form provides structure for consistency and transparency in reporting. For further information on Nature Portfolio policies, see our Editorial Policies and the Editorial Policy Checklist.

Statistics

For all statistical analyses, confirm that the following items are present in the figure legend, table legend, main text, or Methods section.

n/a	Confirmed	
☐	☒	The exact sample size (n) for each experimental group/condition, given as a discrete number and unit of measurement
☐	☒	A statement on whether measurements were taken from distinct samples or whether the same sample was measured repeatedly
☐	☒	The statistical test(s) used AND whether they are one- or two-sided<br>*Only common tests should be described solely by name; describe more complex techniques in the Methods section.*
☐	☒	A description of all covariates tested
☐	☒	A description of any assumptions or corrections, such as tests of normality and adjustment for multiple comparisons
☐	☒	A full description of the statistical parameters including central tendency (e.g. means) or other basic estimates (e.g. regression coefficient) AND variation (e.g. standard deviation) or associated estimates of uncertainty (e.g. confidence intervals)
☐	☒	For null hypothesis testing, the test statistic (e.g. F, t, r) with confidence intervals, effect sizes, degrees of freedom and P value noted<br>*Give P values as exact values whenever suitable.*
☒	☐	For Bayesian analysis, information on the choice of priors and Markov chain Monte Carlo settings
☐	☒	For hierarchical and complex designs, identification of the appropriate level for tests and full reporting of outcomes
☐	☒	Estimates of effect sizes (e.g. Cohen's d, Pearson's r), indicating how they were calculated

Our web collection on statistics for biologists contains articles on many of the points above.

Software and code

Policy information about availability of computer code

Data collection	No software was used for data collection.
Data analysis	Custom scripts were written using Bash Shell Scripting (version 3.2.57), MATLAB (version R2021a: The MathWorks, Inc.), and Python (version 3.7) to analyze the available data; statistical analyses were performed using the SPSS statistical software (version 28: IBM Corp.). Software used to analyze diffusion weighted magnetic resonance imaging (MRI) data included MRtrix3, QSIPrep (https://qsiprep.readthedocs.io/en/latest/; version 0.8.0), and the MITTENS Python library (https://github.com/mattcieslak/MITTENS). Resting-state functional MRI data were analyzed using the CONN (https://web.conn-toolbox.org/home; version 20.b) toolbox.

For manuscripts utilizing custom algorithms or software that are central to the research but not yet described in published literature, software must be made available to editors and reviewers. We strongly encourage code deposition in a community repository (e.g. GitHub). See the Nature Portfolio guidelines for submitting code & software for further information.

Data

Policy information about availability of data

All manuscripts must include a data availability statement. This statement should provide the following information, where applicable:

- Accession codes, unique identifiers, or web links for publicly available datasets
- A description of any restrictions on data availability
- For clinical datasets or third party data, please ensure that the statement adheres to our policy

The Human Connectome Project dataset used in this study is publicly available at https://db.humanconnectome.org/. The University of Pennsylvania sample dataset

analyzed as well as the scripts generated for the purposes of this study are available from the corresponding authors upon reasonable request. Source data are provided with this paper.

Human research participants

Policy information about studies involving human research participants and Sex and Gender in Research.

Reporting on sex and gender	See below.
Population characteristics	Human Connectome Project:<br>A sample of 100 unrelated healthy subjects (54% female; mean age = 29.1±3.7 years; age range = 22-36 years) was drawn from the HCP dataset, as publicly provided by the HCP1200 subjects data release.<br><br>Penn Sample:<br>Healthy individuals (n = 14; 78.6% female; mean age = 22.8±3.2 years; age range = 18-28 years) were prospectively enrolled at the University of Pennsylvania between November 16, 2016 and May 19, 2018, and recruited from the local community.
Recruitment	Human Connectome Project:<br>A sample of 100 unrelated healthy subjects was drawn from the HCP dataset, as publicly provided by the HCP1200 subjects data release.<br><br>Penn Sample:<br>Healthy individuals were prospectively enrolled at the University of Pennsylvania between November 16, 2016 and May 19, 2018, and recruited from the local community.
Ethics oversight	Informed consent was obtained from all Human Connectome Project subjects, and the procedures were approved by the Washington University Institutional Review Board. Informed consent was obtained from all subjects scanned at the University of Pennsylvania, and the procedures were approved by the University of Pennsylvania Institutional Review Board.

Note that full information on the approval of the study protocol must also be provided in the manuscript.

Field-specific reporting

Please select the one below that is the best fit for your research. If you are not sure, read the appropriate sections before making your selection.

- [x] Life sciences
- [ ] Behavioural & social sciences
- [ ] Ecological, evolutionary & environmental sciences

For a reference copy of the document with all sections, see nature.com/documents/nr-reporting-summary-flat.pdf

Life sciences study design

All studies must disclose on these points even when the disclosure is negative.

Sample size	We first utilized the publicly available Human Connectome Project (HCP) dataset containing diffusion imaging and resting-state functional imaging data from 100 unrelated healthy individuals. Our second subject sample contained 14 healthy individuals scanned at the University of Pennsylvania. Despite its small sample size, this dataset contained subjects that had been scanned with particularly high-resolution diffusion imaging, allowing us to analyze their structural and functional organization properties in marked detail.
Data exclusions	After pre-processing the structural and functional scans of our Penn subject sample (n=14; 78.6% female; mean age = 22.8±3.2 years; age range = 18-28 years), we manually examined the generated Quality Control files, to assess the quality of the data. Given that structural and functional connectivity obtained from this sample was being assessed at the voxel-level, we chose to apply particularly conservative quality control criteria when deciding which subjects to include in our analyses: subjects with at least one “bad” slice found (i.e., slices that significantly differed in intensity patterns from the slices acquired before and after) in the pre-processed diffusion images (n = 2) or resting-state functional scans with mean framewise displacement exceeding 0.2 mm (n = 3), were excluded from the analysis. Using these criteria, we ended up including 9 (88.9% female; mean age = 22.8±2.7 years; age range = 19-27 years) out of the total 14 subjects scanned.
Replication	In order to verify the reproducibility of our experimental findings, we analyzed the magnetic resonance imaging (MRI) data acquired from two independent groups of healthy participants (HCP Dataset and Penn dataset) using six different—yet complementary—processing pipelines: atlas-based approaches capitalizing on two commonly used brain parcellation schemes (HCP multi-modal parcellation and Schaefer 400 parcellation), and a voxel-based approach of unprecedented resolution wherein each cortical voxel was treated as a stand-alone brain region; the latter approach allowed us to study brain structural and functional dynamics in marked detail, generating connectivity matrices with sizes ranging from 60,744 x 60,744 to 83,680 x 83,680. Our findings were largely replicated across all processing pipelines.
Randomization	Non-applicable: there was no randomization applied on this study, as all participants were healthy individuals.
Blinding	Non-applicable: all subjects examined were healthy individuals.

Reporting for specific materials, systems and methods

We require information from authors about some types of materials, experimental systems and methods used in many studies. Here, indicate whether each material, system or method listed is relevant to your study. If you are not sure if a list item applies to your research, read the appropriate section before selecting a response.

Materials & experimental systems

n/a	Involved in the study
☒	☐ Antibodies
☒	☐ Eukaryotic cell lines
☒	☐ Palaeontology and archaeology
☒	☐ Animals and other organisms
☒	☐ Clinical data
☒	☐ Dual use research of concern

Methods

n/a	Involved in the study
☒	☐ ChIP-seq
☒	☐ Flow cytometry
☐	☒ MRI-based neuroimaging

Magnetic resonance imaging

Experimental design

Design type: Resting-state functional magnetic resonance imaging (fMRI).

Design specifications: Human Connectome Project dataset:
Resting-state fMRI (gradient-echo echo-planar imaging [EPI] sequence; four runs; 1200 volumes/run, 14:33 min:sec each; voxel size: 2 mm isotropic; repetition time [TR]: 720 ms; echo time [TE]: 33.1 ms).

Penn dataset:
Resting-state fMRI (two runs; 1200 volumes/run, 20:07 min:sec each; voxel size: 3 mm isotropic; TR: 500 ms; TE: 25 ms).

Behavioral performance measures: No behavioral performance measures were considered in this study.

Acquisition

Imaging type(s): Functional, structural, and diffusion.

Field strength: 3T

Sequence & imaging parameters: Human Connectome Project dataset:
Subjects within this sample were scanned on a customized Siemens “Connectome” Skyra 3T scanner (32-channel Siemens head coil) and underwent high-resolution 3T MRI, including T1-weighted (3D Multi-echo Magnetization–Prepared Rapid Gradient Echo [MEMPRAGE] sequence; voxel size: 0.7 mm isotropic; TR: 2400 ms; TE: 2.14 ms), T2-weighted (3D sampling perfection with application-optimized contrasts by using flip angle evolution [SPACE] sequence; voxel size: 0.7 mm isotropic; TR: 3200 ms; TE: 565 ms), resting-state fMRI (gradient-echo echo-planar imaging [EPI] sequence; four runs; 1200 volumes/run, 14:33 min:sec each; voxel size: 2 mm isotropic; TR: 720 ms; TE: 33.1 ms), and high angular resolution diffusion imaging (spin-echo planar imaging sequence; voxel size: 1.25 mm isotropic; TR: 5520 ms; TE: 89.5 ms; max b-value: 3000 s/mm2; 270 non-colinear directions; 18 b0 acquisitions) sequences.

Penn dataset:
Subjects within this sample were scanned using a Siemens Magnetom Prisma 3T scanner (64-channel head/neck coil) and underwent high-resolution 3T MRI, including T1-weighted (3D MEMPRAGE sequence; voxel size: 0.9 mm isotropic; TR: 2500 ms; TE: 2.18 ms), T2-weighted (3D SPACE sequence; voxel size: 0.9 mm isotropic; TR: 3200 ms; TE: 565 ms), resting-state functional MRI (two runs; 1200 volumes/run, 20:07 min:sec each; voxel size: 3 mm isotropic; TR: 500 ms; TE: 25 ms), and diffusion spectrum imaging (voxel size: 1.8 mm isotropic; TR: 4300 ms; TE: 102 ms; max b-value: 5000 s/mm2; 731 directions; 22 b0 acquisitions) sequences.

Area of acquisition: Whole brain.

Diffusion MRI: ☒ Used ☐ Not used

Parameters: Human Connectome Project dataset:
High angular resolution diffusion imaging (spin-echo planar imaging sequence; voxel size: 1.25 mm isotropic; TR: 5520 ms; TE: 89.5 ms; max b-value: 3000 s/mm2; 270 non-colinear directions; 18 b0 acquisitions).

Penn dataset:
Diffusion spectrum imaging (voxel size: 1.8 mm isotropic; TR: 4300 ms; TE: 102 ms; max b-value: 5000 s/mm2; 731 directions; 22 b0 acquisitions).

Preprocessing

Preprocessing software	CONN toolbox (version 20.b).
Normalization	The structural scans are segmented into gray matter, white matter, and cerebrospinal fluid tissue classes using Statistical Parametric Mapping (SPM; version 12), and both structural and functional scans are subsequently normalized into MNI space (180 x 216 x 180 mm3 bounding box; functional scans set to 2 mm isotropic; structural scans set to 1 mm isotropic).
Normalization template	ICBM152.
Noise and artifact removal	A slice-timing correction procedure is followed, correcting for any potential temporal misalignment that may have occurred during the sequential acquisition of the fMRI data; acquisitions with a framewise displacement above 0.9 mm or global BOLD signal changes above 5 standard deviations are flagged as potential outlier scans. Potential confounding effects that are linearly regressed out of the BOLD signal time series include noise components from white matter and cerebrospinal areas, estimated subject motion parameters (i.e., 3 rotation and 3 translation parameters, and their 6 associated first-order derivatives), the aforementioned identified outlier scans, as well as session-related effects (such as constant and linear BOLD signal trends). Temporal frequencies below 0.008 Hz are also removed from the BOLD signal in order to mitigate the effects of low-frequency drifts. Denoising outputs are manually inspected to ensure approximately centered distributions of the resulting functional connectivity data.
Volume censoring	CONN toolbox (version 20.b); see above.

Statistical modeling & inference

Model type and settings	1. One-way analysis of variance (ANOVA) tests were used to statistically compare the overall differences in structure-function coupling (SFC) and temporal SFC variance across the 7 resting-state systems and 5 cyto-architectonic classes defined in the manuscript.<br>2. Comparisons between the four variables of interest: SFC, temporal SFC variance, intracortical myelin content, and the Hurst exponent, were carried out in the form of previously established spatial permutation tests (threshold for significance: p < 0.05). In contrast to bivariate correlations such as Spearman's or Pearson's, spatial permutation tests take into account the potential spatial autocorrelation that might exist between variables and neighboring brain regions as well as hemispheric symmetry, by generating a set of appropriate spatial autocorrelation-preserving null models for each hemisphere.<br>3. We applied a Breusch-Pagan test to test the presence/absence of homoscedasticity in our analyses.<br>4. Multiple linear regression models were used to examine the statistical relationship between two variables, after adjusting for the effects of other pertinent variables. SFC and temporal SFC variance were designated as the dependent variables, whereas intracortical myelin content and the Hurst exponent were designated as the independent variables. In the 'Biological correlates of Structure-Function Coupling: whole-brain perspective' section, we also included the principal functional gradient assignment as an independent variable, so that we could ensure that any potential relationships were not driven by a similar co-variation of the given variables across the same cortical hierarchy. Moreover, to address the non-linear relationship and significant heteroscedasticity between the temporal SFC variance and the Hurst exponent in our voxel-based analyses, we incorporated a non-linear term (square of the Hurst exponent) as an additional independent variable in the 'Biological correlates of Structure-Function Coupling: whole-brain perspective' and 'Biological correlates of Structure-Function Coupling: regional perspective' sections.<br>5. We applied a mediation model to assess potential mediation effects across our variables of interest, using the PROCESS (v3.4) statistical macro for SPSS. Intracortical myelin content was designated as the independent variable, the Hurst exponent of the functional signal time series as the mediator, and the temporal SFC variance as the dependent variable.
Effect(s) tested	Both ANOVA and factorial designs were employed in this study; see above.

Specify type of analysis: ☒ Whole brain ☐ ROI-based ☐ Both

Statistic type for inference (See Eklund et al. 2016)	Voxel-wise.
Correction	FDR.

Models & analysis

n/a	Involved in the study
☐	☒ Functional and/or effective connectivity
☐	☒ Graph analysis
☐	☒ Multivariate modeling or predictive analysis

Functional and/or effective connectivity	Functional connectivity matrices were computed by calculating the Pearson's correlation between the blood oxygen-level dependent signal time series of any two given brain regions.
Graph analysis	Structural and functional connectivity matrices were represented as weighted graphs.
Multivariate modeling and predictive analysis	See "Model type and settings" items 4 and 5 above.
